# Multisite SUMOylation restrains DNA polymerase η interactions with DNA damage sites

**DOI:** 10.1074/jbc.RA120.013780

**Published:** 2020-04-29

**Authors:** Claire Guérillon, Stine Smedegaard, Ivo A. Hendriks, Michael L. Nielsen, Niels Mailand

**Affiliations:** 1 Ubiquitin Signaling Group, Protein Signaling Program, Novo Nordisk Foundation Center for Protein Research, Faculty of Health and Medical Sciences, University of Copenhagen, Blegdamsvej 3B, Copenhagen, Denmark; 2 Proteomics Program, Novo Nordisk Foundation Center for Protein Research, Faculty of Health and Medical Sciences, University of Copenhagen, Blegdamsvej 3B, Copenhagen, Denmark

**Keywords:** sumoylation, DNA damage, DNA damage response, DNA polymerase, post-translational modification (PTM), proliferating cell nuclear antigen (PCNA), translesion DNA synthesis (TLS)

## Abstract

Translesion DNA synthesis (TLS) mediated by low-fidelity DNA polymerases is an essential cellular mechanism for bypassing DNA lesions that obstruct DNA replication progression. However, the access of TLS polymerases to the replication machinery must be kept tightly in check to avoid excessive mutagenesis. Recruitment of DNA polymerase η (Pol η) and other Y-family TLS polymerases to damaged DNA relies on proliferating cell nuclear antigen (PCNA) monoubiquitylation and is regulated at several levels. Using a microscopy-based RNAi screen, here we identified an important role of the SUMO modification pathway in limiting Pol η interactions with DNA damage sites in human cells. We found that Pol η undergoes DNA damage- and protein inhibitor of activated STAT 1 (PIAS1)-dependent polySUMOylation upon its association with monoubiquitylated PCNA, rendering it susceptible to extraction from DNA damage sites by SUMO-targeted ubiquitin ligase (STUbL) activity. Using proteomic profiling, we demonstrate that Pol η is targeted for multisite SUMOylation, and that collectively these SUMO modifications are essential for PIAS1- and STUbL-mediated displacement of Pol η from DNA damage sites. These findings suggest that a SUMO-driven feedback inhibition mechanism is an intrinsic feature of TLS-mediated lesion bypass functioning to curtail the interaction of Pol η with PCNA at damaged DNA to prevent harmful mutagenesis.

## Introduction

Numerous DNA damaging insults are inflicted daily upon the genomes of all living cells (1). If left unrepaired, these lesions can modify the functionality of the genetic material and adversely affect organismal fitness (2). Cellular genomes are particularly vulnerable to DNA damage encountered during chromosomal DNA replication. Genome duplication generally proceeds in a highly processive and accurate manner, due to the extremely high fidelity and proofreading activity of normal, replicative DNA polymerases (3). However, because these polymerases are unable to accommodate most damaged or modified DNA structures in their active sites, the occurrence of DNA lesions in S phase may obstruct the progression of the advancing replication machinery. This presents a serious threat to genome stability as the stalled replication fork may collapse, potentially giving rise to highly cytotoxic DNA double-strand breaks and gross chromosomal instability (4). To mitigate such danger, cells in all domains of life have evolved DNA damage bypass or tolerance strategies enabling the genome duplication machinery to replicate past fork-blocking lesions (5).

The predominant DNA damage bypass mechanism in higher eukaryotes involves specialized translesion DNA synthesis (TLS) polymerases, which unlike replicative DNA polymerases are able to incorporate nucleotides opposite lesions and thus replicate damaged DNA without repairing it (5, 6). However, because TLS polymerases have flexible active sites and lack proofreading activity, their fidelities are orders of magnitude lower than those of replicative DNA polymerases, hence TLS is much more error-prone than normal DNA replication (5). Due to these inherent properties, TLS plays a double-edged role in genome stability maintenance: on the one hand, the bypass of DNA lesions during replication by damage-tolerant polymerases provides cells with the flexibility needed to avoid the potentially much more severe consequences of collapsed replication forks. On the other hand, TLS is a mutagenic process, whose dysregulation has a well-established role in the development of cancer and other severe disorders (7–9). Accordingly, TLS must be kept tightly in check at all times, and multiple regulatory mechanisms have evolved to enable a carefully balanced level of TLS that allows DNA lesion bypass while suppressing excessive mutagenesis.

Mammalian cells encode a range of TLS polymerases, of which several of the most important ones belong to the Y-family of DNA polymerases (6). Among these, DNA polymerase η (Pol η) bypasses cyclobutane pyrimidine dimers (CPDs), the major DNA lesion induced by UV light, with high accuracy and collaborates with Pol ζ in replication past cisplatin-induced DNA intra-strand cross-links (6, 10–16). Reflecting its critical physiological importance, Pol η dysfunctionality leads to a variant form of xeroderma pigmentosum (XPV), a disorder characterized by a high incidence of pigmentation changes, sun sensitivity, and skin cancer, likely caused by the compensatory action of more mutagenic back-up TLS polymerases (17–19).

Activation of TLS entails the replacement of a replicative DNA polymerase bound to the proliferating cell nuclear antigen (PCNA) clamp with a TLS polymerase. Multilayered regulatory mechanisms, in which signaling by ubiquitin and ubiquitin-like modifiers (UBLs) has a central role, exert tight control over TLS polymerase recruitment to damaged DNA (20, 21). In particular, DNA damage-dependent PCNA monoubiquitylation at Lys-164 by the RAD18-RAD6 E3 ubiquitin ligase complex has a key function in stimulating TLS by generating a binding platform for Y-family TLS polymerases, which harbor both PCNA-interacting protein (PIP) boxes and ubiquitin-binding motifs that enable them to preferentially interact with monoubiquitylated PCNA and displace replicative polymerases (22–26). High affinity of Pol η for monoubiquitylated PCNA, visible in cells as the formation of discrete nuclear Pol η foci upon DNA damage, is mediated by a C-terminal ubiquitin-binding zinc finger (UBZ) domain, three PIP boxes and a nuclear localization signal (NLS) (25, 27–32). Due to the error-prone nature and lack of processivity of TLS polymerases, they must be replaced with a replicative DNA polymerase following damage bypass in a second polymerase exchange that is also subject to elaborate regulatory control. Several mechanisms, including PCNA deubiquitylation by USP1 and the action of a number of factors including CRL4^CDT2^ and PAF15, and post-translational modifications (PTMs) by phosphorylation, ubiquitylation, and *O-*GlcNAcylation, directly or indirectly contribute to limiting Pol η interactions with DNA damage sites (33–39), but the relative contributions and precise mechanistic underpinnings of these processes are not yet fully understood.

Given the critical importance of limiting interactions of Pol η and other TLS polymerases with PCNA at damaged DNA to avoid unwanted genetic changes, we used a high-throughput imaging-based screen as an unbiased approach to identify signaling processes controlling the access of Pol η to DNA damage sites in human cells. We found that SUMOylation impinging directly on Pol η bound to PCNA at DNA damage sites plays an important role in limiting this interaction. We established that this regulatory circuit involves DNA damage-dependent multisite SUMOylation of Pol η that renders it susceptible to extraction from DNA damage sites by the action of SUMO-targeted ubiquitin ligases (STUbLs). These findings suggest the existence of a SUMO-driven negative-feedback loop operating to limit the association of Pol η with PCNA at damaged DNA and prevent unscheduled mutagenesis.

## Results

### A microscopy-based RNAi screen reveals SUMO-dependent regulation of Pol η association with DNA damage sites

**Figure 1.**
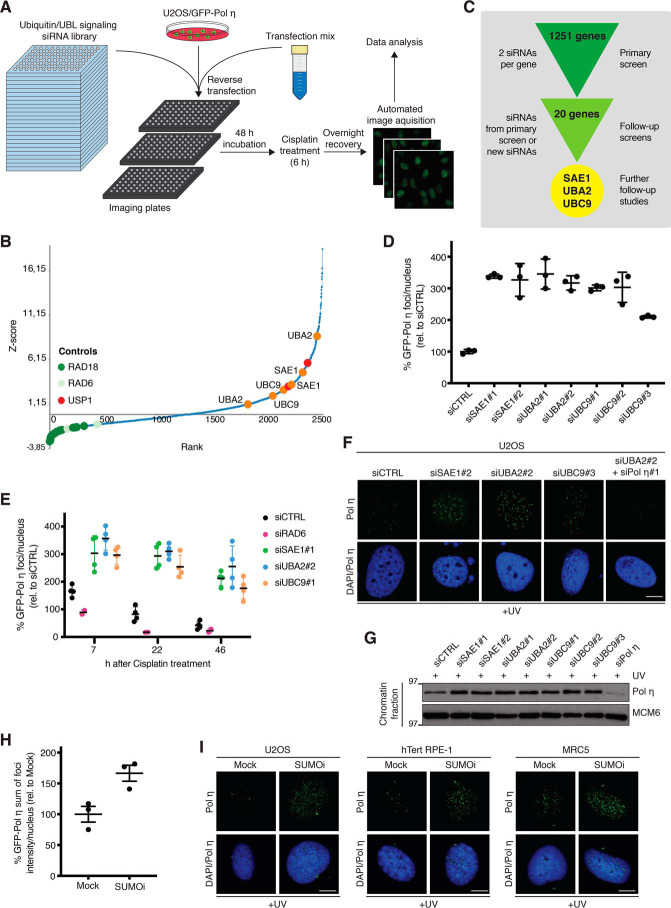
**A microscopy-based screen reveals SUMO-dependent regulation of Pol η association with DNA damage sites.** *A,* experimental set-up of high-throughput microscopy-based screen for ubiquitin and UBL signaling network components regulating Pol η interaction with sites of cisplatin-induced DNA damage. See text for details. *B,* results of the screen outlined in *A*. Scatter plot shows ranked Z-scores of cisplatin-induced GFP-Pol η foci counts of all siRNAs in the library (2 siRNAs per gene), determined by QIBC analysis. Controls (RAD18-RAD6 and USP1) are indicated in *green* and *red*, respectively. SUMO E1 (SAE1 and UBA2) and E2 (UBC9) enzymes are indicated in *orange*. See also Table S1. *C,* workflow of primary and validation screens, and hit selection. See also Table S2. *D,* results of the validation screen analyzing GFP-Pol η foci count in U2OS/GFP-Pol η cells transfected with the indicated siRNAs, exposed to cisplatin for 6 h, and fixed 16 h later and quantified using QIBC analysis (mean ± S.D.; *n* = 3 independent experiments; ≥294 cells quantified per condition). *E,* results of validation screen analyzing kinetics of GFP-Pol η foci formation in cells treated as in *D* (mean ± S.D.; *n* = 3 independent experiments; ≥3,000 cells quantified per condition). *F,* representative images of endogenous Pol η foci formation in U2OS cells transfected with the indicated siRNAs and exposed to UV. *Scale bar*, 10 μm. *G,* immunoblot analysis of chromatin-enriched fractions of U2OS cells treated as in *F. H,* U2OS/GFP-Pol η cells were preincubated or not with SUMOi for 30 min, exposed to UV (20 J/m^2^), and collected 6 h later. The sum of GFP-Pol η foci intensity per nucleus was quantified by QIBC (mean ± S.E.M.; *n* = 3 independent experiments; ≥7,482 cells quantified per condition). *I,* representative images of endogenous Pol η foci formation in U2OS, hTert RPE-1, and MRC5 cells treated as in *H. Scale bar*, 10 μm.

To gain better insights into how signaling by ubiquitin and UBLs control TLS, we performed a high-throughput microscopy-based screen for regulators of Pol η association with damaged DNA, using a human U2OS osteosarcoma cell line engineered to stably express GFP-tagged Pol η at a low level (40) and an siRNA library targeting 1251 genes involved in ubiquitin- and UBL-mediated signaling (41) (Fig. 1*A*). To this aim, siRNA-transfected cells were treated with cisplatin to induce DNA damage, and acquisition and quantification of the resulting GFP-Pol η foci were performed using quantitative image-based cytometry (QIBC) (42), allowing for the ranking of all siRNAs according to their impact on GFP-Pol η foci counts (Fig. 1, *A* and *B*; Table S1). As expected, siRNAs targeting RAD18 and RAD6, which catalyze PCNA monoubiquitylation and are required for Pol η recruitment to DNA damage sites (22, 24), were among the strongest suppressors of cisplatin-induced GFP-Pol η foci, whereas knockdown of the PCNA deubiquitinase USP1 led to increased GFP-Pol η foci formation (Fig. 1*B*; Table S1), validating our experimental screen setup. Consistent with previous work by others and us (40, 43), knockdown of the SPRTN protease also enhanced Pol η DNA damage foci counts (Table S1). In addition to these positive controls, the siRNA screen revealed a range of additional Ub/UBL signaling factors, whose depletion markedly enhanced GFP-Pol η recruitment to cisplatin-induced DNA damage, suggesting potential functions of these proteins in restricting Pol η interaction with damaged DNA (Fig. 1*B*; Table S1). Validation screens for the strongest hits using siRNAs from the primary screen and independent siRNAs subsequently revealed a range of high-confidence hits whose depletion enhanced GFP-Pol η association with sites of cisplatin-induced DNA damage (Fig. 1*C*; Table S2). Notably, these included both the SUMO E1 (SAE1 and UBA2) and E2 (UBC9) enzymes that are essential for SUMOylation and whose individually reduced expression strongly increased both Pol η foci counts and intensity (Fig. 1, *B–D*; Fig. S1, *A* and *B*), suggesting a potential function of the SUMO modification pathway in limiting Pol η interactions with DNA damage sites. In agreement with this notion, a time course analysis showed that depletion of SAE1, UBA2, or UBC9 led to a markedly elevated proportion and intensity of cisplatin-induced GFP-Pol η foci that persisted long after these structures returned to baseline levels in control cells (Fig. 1*E*; Fig. S1*C*). This suggested that SUMOylation has a role in promoting Pol η clearance from sites of cisplatin-induced DNA damage. In addition to bypassing cisplatin-induced DNA intra-strand cross-links, Pol η also performs highly accurate insertion across CPDs induced by UV radiation (44). We therefore asked if down-regulation of SAE1, UBA2, or UBC9 would also enhance Pol η accumulation at sites of UV-induced DNA damage. Indeed, depleting either of these factors increased endogenous Pol η foci number and intensity, as well as overall Pol η chromatin association, upon exposure to UV (Fig. 1, *F* and *G*). Importantly, acute suppression of SUMOylation by the small-molecule SUMO E1 enzyme inhibitor ML-792 (SUMOi) (45) phenocopied the effect of depleting SAE1, UBA2, or UBC9, by enhancing UV-induced Pol η foci formation in U2OS cells, MRC5 normal lung fibroblasts, and nontransformed RPE-1 retinal pigment epithelial cells (Fig. 1, *H* and *I*; Fig. S1*D*). Together, these data support a role for SUMO-mediated signaling in antagonizing Pol η interactions with DNA damage sites that is operational in both normal and cancer cells.

### Pol η is polySUMOylated in response to DNA damage

**Figure 2.**
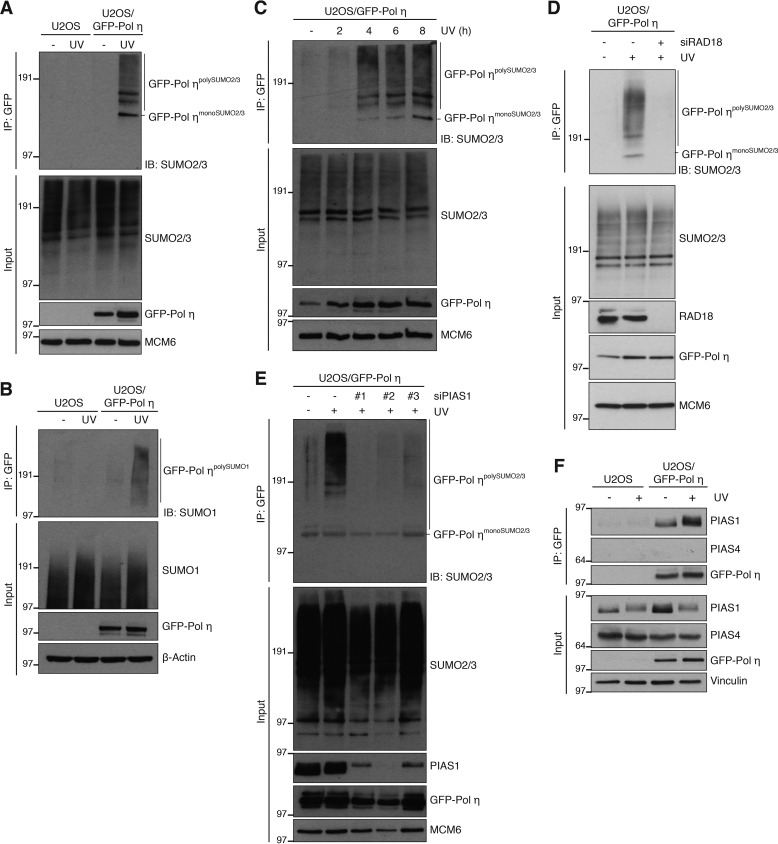
**DNA damage-dependent polySUMOylation of Pol η.** *A,* U2OS or U2OS/GFP-Pol η cells were left untreated or exposed to UV, lysed, and subjected to GFP immunoprecipitation (*IP*) under denaturing conditions followed by immunoblotting (*IB*) with the indicated antibodies. *B,* as in *A*, but using SUMO1 antibody for immunoblotting. *C,* Pol η polySUMOylation at different time points after UV exposure was analyzed as in *A. D,* U2OS/GFP-Pol η cells treated or not with RAD18 siRNA and UV as indicated were processed for analysis of Pol η polySUMOylation as in *A. E,* as in *A*, using the indicated PIAS1 siRNAs. *F,* U2OS or U2OS/GFP-Pol η cells left untreated or exposed to UV were lysed and subjected to GFP IP under native conditions and immunoblotted with the indicated antibodies.

We next set out to address how SUMOylation regulates Pol η interactions with DNA damage sites. First, we established that siRNA-mediated depletion of UBA2, SAE1, or UBC9 had no discernible effect on cell cycle profiles and PCNA monoubiquitylation levels (Fig. S2, *A* and *B*), indicating that the enhanced association of Pol η with sites of DNA damage in these cells was not an indirect consequence of altered cell cycle progression or DNA damage levels. We therefore considered the possibility that the impact of SUMOylation on Pol η association with damaged DNA might involve direct SUMO modification of Pol η itself. Indeed, GFP-Pol η pulldowns under denaturing conditions revealed robust UV-induced modification of Pol η by both SUMO1 and SUMO2/3 (Fig. 2, *A* and *B*). Pol η has previously been reported to undergo SUMOylation on Lys-163 in unperturbed cells overexpressing both Pol η and SUMO isoforms (46). Under our experimental conditions, involving endogenous SUMO and ectopic Pol η expressed at low levels, we found that Pol η was predominantly modified by polySUMOylation upon DNA damage (Fig. 2, *A–C*). Although a point mutation preventing SUMOylation of Lys-163 (K163R) abolished Pol η monoSUMOylation, consistent with previous findings (46), it had no detectable impact on UV-induced GFP-Pol η polySUMOylation (Fig. S2*C*), implying that this modification is not specifically targeted to Lys-163. The onset of UV-induced Pol η polySUMOylation correlated with the appearance of UV-induced Pol η foci (Fig. S2*D*) and the time needed for Pol η to encounter and bypass UV lesions (6, 47). This suggests that Pol η becomes susceptible to SUMOylation upon its recruitment to PCNA at damaged DNA. Consistent with this notion, preventing Pol η recruitment to monoubiquitylated PCNA by knockdown of RAD18 abrogated UV-induced GFP-Pol η polySUMOylation (Fig. 2*D*). We next screened for SUMO E3 ligases involved in DNA damage-dependent Pol η polySUMOylation. Individual depletion of known SUMO E3s suggested PIAS1 as the prospective ligase (Fig. S2*E*). We substantiated this observation using several independent PIAS1 siRNAs that abolished UV-induced Pol η polySUMOylation but did not noticeably affect cell cycle status and PCNA monoubiquitylation (Fig. 2*E*; Fig. S2, *B* and *F*). Consistent with a causal role of PIAS1 in promoting DNA damage-induced Pol η SUMOylation, PIAS1 but not PIAS4, another DDR-associated SUMO E3 ligase (48), co-immunoprecipitated with GFP-Pol η in a manner that was stimulated by UV radiation (Fig. 2*F*). We conclude that Pol η undergoes PIAS1-dependent polySUMOylation upon its DNA damage-induced association with PCNA.

### PIAS1 and SUMO-targeted ubiquitin ligases regulate Pol η interactions with DNA damage sites

**Figure 3.**
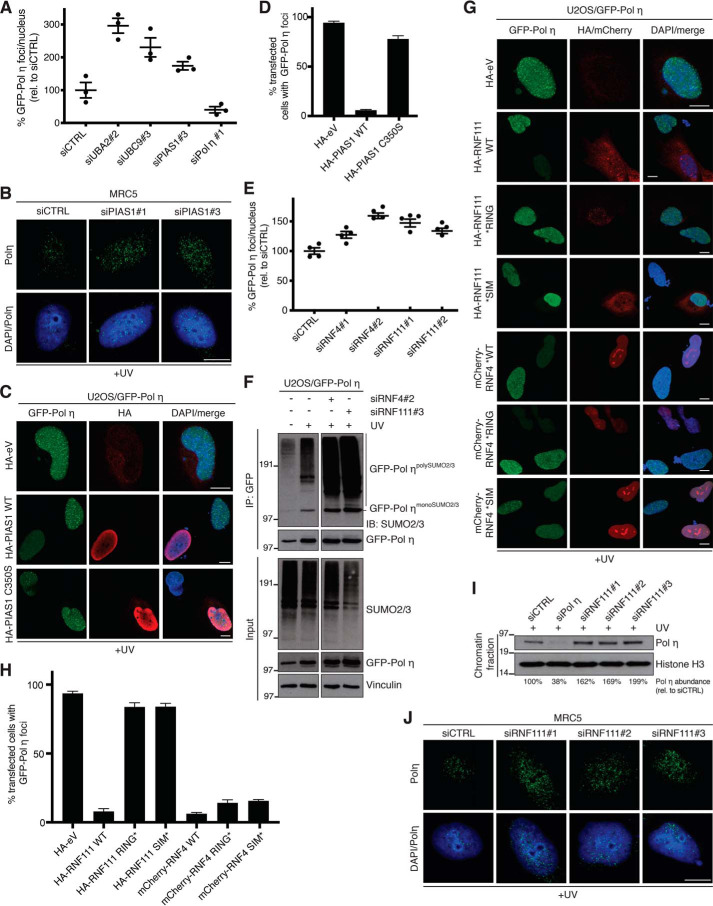
**PIAS1 and STUbL activity promotes Pol η displacement from DNA damage sites.** *A,* GFP-Pol η foci count in U2OS/GFP-Pol η cells transfected with the indicated siRNAs, exposed to UV, and fixed 6 h later was quantified using QIBC analysis (mean ± S.E.M.; *n* = 3 independent experiments; ≥1991 cells quantified per condition). *B,* representative images of endogenous Pol η foci formation in MRC5 cells transfected with the indicated siRNAs and exposed to UV. *Scale bar*, 10 μm. *C,* representative images of U2OS/GFP-Pol η cells transfected with the indicated HA-PIAS1 expression plasmids or empty vector (*eV*), exposed to UV, and immunostained with HA antibody. *Scale bar*, 10 μm. *D,* quantification of data in *C* (mean ± S.E.M.; *n* = 3 independent experiments; ≥50 cells analyzed per condition). *E,* GFP-Pol η foci count in U2OS/GFP-Pol η cells transfected with the indicated siRNAs analyzed as in *A* (mean ± S.E.M.; *n* = 4 independent experiments; ≥1254 cells quantified per condition). *F,* U2OS/GFP-Pol η cells treated with the indicated RNF4 or RNF111 siRNAs and exposed to UV were lysed and subjected to GFP IP under denaturing conditions followed by immunoblotting (*IB*) with the indicated antibodies. *G,* representative images of U2OS/GFP-Pol η cells transiently transfected with the indicated RNF4 or RNF111 expression constructs or empty vector (*eV*), and treated as in *C. Scale bar*, 10 μm. *H,* quantification of data in *G* (mean ± S.E.M.; *n* = 3 independent experiments; ≥50 cells analyzed per condition). *I,* immunoblot analysis of chromatin-enriched fractions of U2OS cells transfected with the indicated RNF111 siRNAs and exposed to UV. Association of endogenous Pol η with UV-damaged chromatin (normalized to Histone H3 abundance) was quantified using ImageJ. *J,* representative images of endogenous Pol η foci formation in MRC5 cells transfected with the indicated RNF111 siRNAs and exposed to UV. *Scale bar*, 10 μm.

We next analyzed whether and how PIAS1-dependent polySUMOylation of Pol η impacts its interaction with DNA damage sites. Consistent with a role of SUMOylation in limiting Pol η retention at damaged DNA, we found that like UBA2 or UBC9 knockdown, depletion of PIAS1 enhanced GFP-Pol η foci number and intensity in U2OS cells (Fig. 3*A*; Fig. S3*A*). Similar observations were made for endogenous Pol η in MRC5 cells (Fig. 3*B*). Moreover, overexpression of WT PIAS1 but not a catalytically inactive point mutant (C350S) led to quantitative removal of UV-induced Pol η foci (Fig. 3, *C* and *D*), suggesting that PIAS1-mediated polySUMOylation of Pol η promotes its dissociation from PCNA at DNA damage sites. SUMOylation of chromatin-bound proteins can trigger their subsequent extraction via SUMO-targeted ubiquitin ligases (STUbLs) (49–51), and we therefore asked whether the known human STUbLs, RNF4 and RNF111, might be effectors of PIAS1 SUMO E3 ligase-mediated dissociation of Pol η from DNA damage foci. In support of this possibility, knockdown of RNF4 or RNF111 using independent siRNAs enhanced GFP-Pol η foci counts and intensity (Fig. 3*E*; Fig. S3, *B–D*). Moreover, loss of RNF4 or RNF111 strongly enhanced UV-induced Pol η polySUMOylation accompanied by modestly elevated Pol η expression levels (Fig. 3*F*), further indicating that impaired STUbL functionality interferes with the proper processing of SUMO-modified Pol η. To determine whether the potential roles of RNF4 and RNF111 in regulating Pol η interactions with DNA damage sites require their STUbL activities, we analyzed GFP-Pol η foci formation in cells overexpressing RNF4 or RNF111 WT or mutant forms containing inactivating substitutions within their SUMO-interacting (*SIM) or catalytic RING (*RING) motifs. Supporting a direct involvement of RNF111 STUbL activity in promoting extraction of SUMO-modified Pol η from damaged chromatin, we found that overexpressed RNF111 WT but not the *SIM and *RING mutants suppressed GFP-Pol η localization to DNA damage sites (Fig. 3, *G* and *H*). By contrast, both WT and mutant alleles of overexpressed RNF4 impaired GFP-Pol η foci formation after UV (Fig. 3, *G* and *H*), suggesting that modulation of RNF4 functionality impacts Pol η association with damaged DNA via a mechanism that is independent of its STUbL activity and whose precise basis awaits to be established. Finally, using independent siRNAs we validated that depletion of RNF111 led to an increased association of endogenous Pol η with DNA damage sites and UV-damaged chromatin (Fig. 3, *I* and *J*). Collectively, the data suggest that PIAS1-dependent polySUMOylation of Pol η may trigger its STUbL-mediated ubiquitylation and concomitant dissociation from DNA damage sites.

### Multisite SUMOylation of Pol η underlies its PIAS1- and RNF111-dependent clearance from DNA damage sites

**Figure 4.**
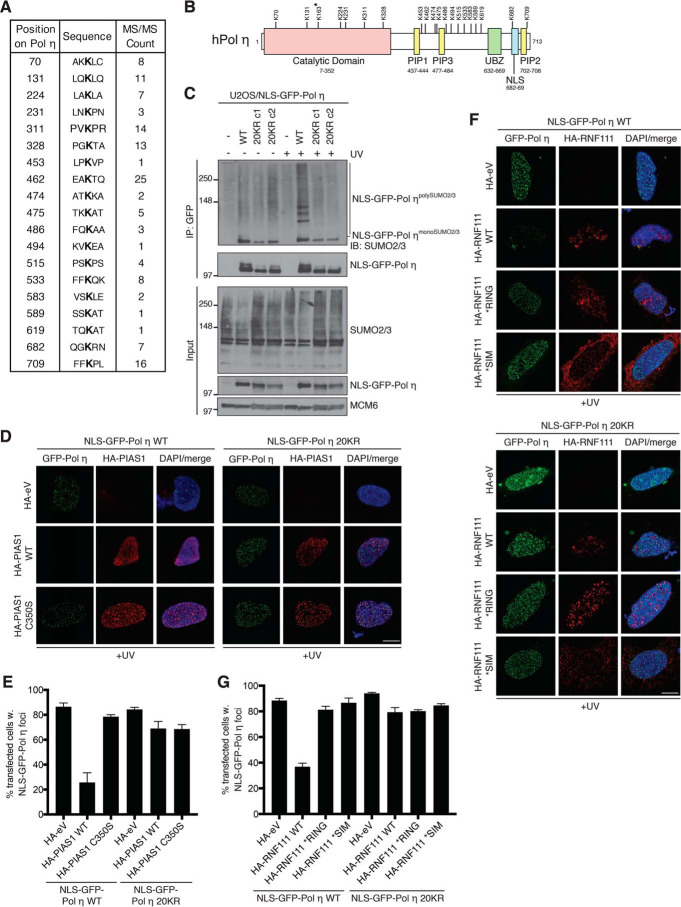
**Multisite SUMOylation of Pol η underlies its PIAS1- and RNF111-dependent extraction from DNA damage sites.** *A,* table showing identification of endogenous Pol η SUMOylation sites using an augmented K0-SUMO proteomic strategy coupled with deep MS data analysis. See also Table S3 and Fig. S4. *B,* schematic diagram of human Pol η, depicting the location of functional motifs and identified SUMOylation sites. A previously reported Pol η SUMOylation site, Lys-163 (37), which did not pass the significance threshold but was mutated along with the 19 high-confidence SUMOylation sites to generate the Pol η 20KR mutant, is indicated by the *asterisk. C,* U2OS cells or derivative clones stably expressing GFP-Pol η WT or 20KR mutant (clone 1 (c1) and clone 2 (c2)) were left untreated or exposed to UV, lysed, and subjected to GFP IP under denaturing conditions and immunoblotted (*IB*) with the indicated antibodies. *D,* representative images of U2OS cells co-transfected with the indicated GFP-Pol η and HA-PIAS1 expression constructs, exposed to UV and immunostained with HA antibody. *Scale bar*, 10 μm. *eV*, empty vector. *E,* quantification of data in *D* (mean ± S.E.M.; *n* = 3 independent experiments; ≥55 cells analyzed per condition). *F,* representative images of U2OS cells co-transfected with the indicated GFP-Pol η and HA-RNF111 expression constructs, exposed to UV, and immunostained with HA antibody. *Scale bar*, 10 μm. *G,* quantification of data in *F* (mean ± S.E.M.; *n* = 3 independent experiments; ≥100 cells analyzed per condition).

To directly analyze the role of SUMOylation in constraining Pol η association with damaged DNA, we sought to map the SUMO modification sites in Pol η. Using an augmented K0-SUMO proteomic strategy coupled with deep MS data analysis (52), we identified 19 SUMOylation sites in human Pol η (Fig. 4, *A* and *B*; Fig. S4; Table S3), which is consistent with its observed polySUMOylation in biochemical experiments (Fig. 2, *A–C*) and provides direct evidence that endogenous Pol η is modified by multisite SUMOylation. Although some of these sites are located within the polymerase domain, the majority of mapped Pol η SUMOylation sites cluster in its C-terminal half containing a NLS as well as PIP and UBZ motifs required for targeting Pol η to DNA damage sites (Fig. 4*B*). The identification of these SUMOylation sites provided an opportunity to directly address whether SUMOylation of Pol η promotes its turnover from DNA damage sites, and we therefore generated a Pol η mutant in which all of the 19 SUMOylated lysine residues were mutated to arginine, to prevent their SUMO-dependent modification. We additionally introduced an arginine substitution at Lys-163, the previously reported Pol η SUMOylation site (46) that did not, however, pass the significance threshold in our MS data analysis (Table S3). Substituting the 20 lysine residues with arginines (20KR) affected the subcellular localization of Pol η (data not shown), likely due to the K682R mutation located within its NLS that has previously been reported to impair nuclear localization (Fig. 4*B*) (25). To circumvent this issue, we introduced an NLS into the GFP-Pol η WT and 20KR constructs, resulting in their exclusively nuclear localization (see Fig. 4*D*). Pulldowns of the GFP-Pol η alleles under denaturing conditions confirmed that the 20KR mutations largely abolished UV-induced polySUMOylation of Pol η but not its functionality in DNA damage bypass *per se* (Fig. 4*C*; Fig. S3, *E* and *F*). Prompted by this observation, we then tested whether the lack of DNA damage-induced Pol η SUMOylation would render it resistant to PIAS1- and RNF111-mediated displacement from DNA damage sites. Importantly, the 20KR mutant fully retained the ability to accumulate in UV-induced nuclear foci (Fig. 4*D*). However, unlike WT Pol η the 20KR mutant was insensitive to the ability of elevated PIAS1 catalytic activity to suppress Pol η foci formation (Fig. 4, *D* and *E*). Similarly, we found that overexpression of WT RNF111 but not the *RING and *SIM mutants reduced GFP-Pol η WT association with DNA damage sites, whereas the 20KR mutant was refractory to the impact of elevated RNF111 STUbL activity (Fig. 4, *F* and *G*). Together, these data lend strong support to the notion that UV-induced, PIAS1-dependent multisite SUMOylation of Pol η promotes its STUbL-mediated turnover from DNA damage sites, providing a potential means to curb Pol η activity during DNA damage bypass and thereby mitigate the risk of unscheduled mutagenesis.

## Discussion

The access of Pol η and other low-fidelity TLS polymerases to the DNA synthesis machinery must be tightly regulated to avoid unscheduled mutagenesis, and PTMs have been shown to control TLS activity at several levels. Based on an unbiased screen for PTM-mediated regulators of Pol η interaction with DNA damage sites, we here provide evidence that one cellular mechanism for limiting Pol η-mediated TLS involves a built-in feedback inhibition circuit driven by SUMOylation. We show that Pol η undergoes robust polySUMOylation concomitant with, and dependent on, its recruitment to monoubiquitylated PCNA at DNA damage sites. These SUMO modifications in turn render Pol η susceptible to STUbL-mediated extraction from DNA damage sites, thereby helping to ensure that direct Pol η interactions with PCNA at damaged DNA are kept highly dynamic and transient. Indeed, it has previously been shown that the residence time of human Pol η in nuclear foci is less than 1 s (27). The notion that Pol η becomes SUMOylated upon its recruitment to DNA-loaded PCNA is well-aligned with recent work demonstrating that compared with mature chromatin, the protein landscape around newly replicated DNA is enriched in SUMO modifications (53, 54). Thus, high local SUMOylation activity in the context of the replisome may contribute to an environment in which the docking of TLS polymerases and other auxiliary DNA replication factors can be carefully controlled and dynamically regulated in accordance with the status and integrity of ongoing DNA synthesis. It is possible that such a SUMO-mediated regulatory scheme could operate more broadly within TLS, as we have observed that other Y-family polymerases can also be modified by polySUMOylation in cells.^3^ Notably, our augmented proteomic strategy for mapping SUMOylation sites revealed that Pol η SUMOylation impacts an extensive range of lysine residues distributed throughout its domains, suggesting a high degree of promiscuity in the modification of individual sites. This observation further supports a scenario in which the replisome harbors high SUMOylation activity toward solvent-exposed lysine residues in incoming factors that allows for their subsequent turnover in a manner that may be largely non-site specific. Consistent with this idea, we found that simultaneous mutation of all mapped SUMOylation sites in Pol η, but not subsets of SUMO-conjugation sites, is necessary to render Pol η refractory to the ability of elevated PIAS1 and STUbL activity to promote its extraction from DNA damage sites.^1^

A common mechanism by which SUMO-modified proteins are evicted from chromatin entails their STUbL-mediated ubiquitylation. Previous work by others and us (49, 55–58) demonstrated important regulatory roles of the human STUbLs RNF4 and RNF111 in promoting extraction of a range of genome stability maintenance factors, including XPC, FANCD2-FANCI, RPA, and MDC1, from DNA damage sites. The findings reported here suggest that by driving the turnover of SUMOylated Pol η from DNA damage sites the involvement of STUbL activity in the DNA damage response extends to TLS, although the depletion of RNF4 or RNF111 enhanced Pol η foci number and intensity to a milder extent than knockdown of SUMO E1 and E2 enzymes. This raises the possibility that SUMOylated Pol η may also be susceptible to displacement from monoubiquitylated PCNA via other mechanisms. We found that whereas the association of Pol η with DNA damage foci was responsive to both manipulation of RNF4 and RNF111 status, only RNF111 promoted Pol η dissociation from these locales in a manner that depended on the integrity of its RING and SIM domains, as expected from the *bona fide* action of a STUbL. It is possible that altering RNF4 functionality could impact Pol η retention at damaged DNA via a more general, indirect effect on SUMO pathway homeostasis. In line with this notion, both UBC9 and a number of E3 SUMO ligases have been reported to be direct substrates of RNF4 ubiquitin ligase activity (59). Interestingly, recent work suggested that RNF111 shows a preference for polySUMO2/3 chains capped by SUMO1 (60), in good agreement with our finding that Pol η is modified by both SUMO2/3 and SUMO1 in response to UV.

Our observation that DNA damage-induced Pol η polySUMOylation requires RAD18 and the PIAS1 SUMO E3 ligase is consistent with recent findings reporting a specific role of Pol η SUMOylation at Lys-163 in promoting its interaction with replication forks (46). However, whereas our biochemical data support monoSUMOylation of Pol η at Lys-163, our deep MS-based profiling of SUMOylation sites on endogenous proteins and biochemical assays using ectopic Pol η expressed at low levels in cells clearly show that the pattern of Pol η SUMO modifications in cells is considerably more complex, involving multisite SUMOylation that impacts at least 19 lysine residues and is highly responsive to DNA damage. Interestingly, mutations disrupting two of these SUMO modification sites (Lys-224 and Lys-589) have been identified in XPV patients (61–63). Together with previous data (46), these findings suggest that at least two modes of SUMO-mediated regulation of Pol η are operational in cells. First, monoSUMOylation of Pol η on Lys-163 may promote its association with the replication machinery in undamaged cells (46). Second, our data show that Pol η undergoes DNA damage-dependent polySUMOylation on multiple lysine residues in a manner that is independent of Lys-163 and has an important role in preventing excessive Pol η interactions with DNA damage sites. Future work should address in more detail the mechanisms and significance of the complex and multifaceted roles of SUMOylation in controlling the access of Pol η, and possibly other TLS polymerases, to different DNA structures. In summary, our findings suggest that stimulation of Pol η-dependent DNA damage bypass triggers its own inactivation via a SUMO-mediated feedback inhibition mechanism, offering a simple yet efficient means of restraining TLS activity to avoid the harmful consequences of excessive mutagenesis.

## Experimental procedures

### Plasmids and siRNAs

Plasmid expressing eGFP-tagged human Pol η WT was a kind gift from A. Lehmann (University of Sussex, UK). Plasmids encoding Strep/HA-RNF111 WT, *RING and *SIM, and mCherry-RNF4 WT, *RING and *SIM were described previously (49, 55). A cDNA encoding Pol η 20KR (K70R, K131R, K163R, K224R, K231R, K311R, K328R, K453R, K462R, K474R, K475R, K486R, K499R, K515R, K533R, K583R, K589R, K619R, K682R, K709R) was produced as a synthetic gene (Thermo Fisher, United States) and cloned into pEGFP-C3. Generation of Pol η point mutants (K86R, K163R, K261R, K637R, K661R, K683R, K686R, K694R, K709R) and the insertion of an extra NLS sequence from SV40 large T antigen into pEGFP-C3 were performed using site-directed mutagenesis (Q5 Site-directed Mutagenesis Kit, New England Biolabs, United States). All constructs were verified by sequencing. Plasmid transfections were done with FuGENE 6 (Roche Applied Science) or Genejuice (Novagene) according to the manufacturers' instructions. siRNA transfections were performed using Lipofectamine RNAiMAX (Invitrogen) according to the manufacturer's protocol. siRNA target sequences used in this study were: siCTRL, 5′-GGGAUACCUAGACGUUCUA-3′; siMMS21, 5′-CUCUGGUAUGGACACAGCU-3′; siPC2, 5′-CGUGGGAACCGGAGGAGAA-3′; siPIAS1#1, 5′-UAAAGCUCUAGAAUGAUCC-3′; siPIAS1#2, 5′-UAGCUAUUUCACUGUCUGGAUCCGC-3′; siPIAS1#3, 5′-UAUUAAUGUAGCUUGUGUCUACAGC-3′; siPIAS1#4, 5′-CGAAUGAACUUGGCAGAAA-3′; siPIAS2, 5′-CUUGAAUAUUACAUCUUUA-3′; siPIAS3, 5′-CCCUGAUGUCACCAUGAAA-3′; siPIAS4#1, 5′-CAAGACAGGUGGAGUUGAU-3′; siPolη#1, 5′-UAAACCUUGUGCAGUUGUA-3′; siPolη#2 (targeting the 3′UTR), 5′-GAGAAAGGGAAUUAUGAAA-3′; siRAD6A, 5′-GAACAAGCUGGCGUGAUU-3′; siRAD6B, 5′-CAAACGAGAAUAUGAGAAA-3′; siRAD18, 5′-ACUCAGUGUCCAACUUGCU-3′; siRanBP2, 5′-GGACAGUGGGAUUGUAGUG-3′; siRNF4#1, 5′-GAAUGGACGUCUCAUCGUU-3′; siRNF4#2, 5′-GACAGAGACGUAUAUCUGA-3′; siRNF111#1, 5′-AGAAGGAAAUGAAUGGUAA-3′; siRNF111#2, HS S182646 (Invitrogen, Thermo Scientific); siRNF111#3, GGAUAUUAAUGCAGAGGAA; siTopors, 5′-CAAGGAGCCUGUCUAGUAA-3′; siSAE1#1, 5′-AGAAGAAACCAGAGUCAUU-3′; siSAE1#2, 5′-CAAAGUUAGCCAAGGAGUA-3′; siUBA2#1, 5′-GGAAAUUAGAUGAGAAAGA-3′; siUBA2#2, 5′-CCAAAUUGAAGAUGGGAAA-3′; siUBC9#1, 5′-GUAGCUGUCCCAACAAAGA-3′; siUBC9#2, 5′-UCGAACCACCAUUAUUUCACCCGAA-3′; and siUBC9#3, 5′-GCUCAAGCAGAGGCCUACACGAUUU-3′.

### Cell culture

Human U2OS osteosarcoma cells, hTert RPE-1 retinal pigment epithelial cells, and MRC5 fibroblast were obtained from ATCC. U2OS and MRC5 were cultured in Dulbecco's modified Eagle's medium and hTert RPE-1 in F-12/Dulbecco's modified Eagle's medium containing 10% fetal bovine serum and 5% penicillin-streptomycin. All cell lines were regularly tested negative for mycoplasma infection. The U2OS cell line stably expressing GFP-Pol η at a low and homogenous level was described previously (40). To generate cell lines stably expressing NLS-GFP-Pol η WT or 20KR mutant, U2OS cells were co-transfected with pBabe-Puro plasmid and expression constructs encoding NLS-GFP-Pol η WT or 20KR, and subsequently selected with Puromycin. Individual clones were screened for GFP-Pol η positivity by microscopy. Unless otherwise indicated, the following concentrations of drugs and genotoxic agents were used: Cisplatin (30 μm for 6 h; Merck), UV radiation (UV; 20 J/m^2^), and ML-792 (SUMO-E1i) (l μm; MedKoo Biosciences).

### Immunofluorescence, microscopy, and QIBC

Cells were pre-extracted in PBS containing 0.2% Triton X-100 for 2 min on ice and fixed in 4% formaldehyde. For visualization of endogenous Pol η, pre-extraction was done in pre-extraction buffer (10 mm Tris-HCl, pH 7.4, 2.5 mm MgCl_2_, 0,5% Nonidet P-40, 1 mm phenylmethylsulfonyl fluoride) for 8 min on ice, after which the cells were washed with PBS for 2 min on ice and fixed in 4% formaldehyde. Immunofluorescence staining was performed by incubating fixed cells with primary or secondary antibodies (Alexa Fluor; ThermoFisher Scientific) diluted in PBS containing 10% BSA for 1 h at room temperature. Cell cycle profiles were determined by pulse-labeling cells for 20 min with 10 μm 5-ethynyl-2′-deoxyuridine (EdU) immediately before pre-extraction and fixation. Staining was done using the Click-iT Plus EdU Alexa Fluor 647 Imaging kit (ThermoFisher Scientific) according to the manufacturer's instructions. Representative images were acquired with a confocal microscope (LSM 880; Carl Zeiss), mounted on a confocal laser-scanning microscope (Zeiss AxioObserver.Z1; Carl Zeiss) equipped with a Plan Apochromat ×40/1.3 NA oil immersion objective. Image acquisition was performed with ZEN 2.1 software (Carl Zeiss). QIBC was performed as described previously (42). Images were acquired using a wide-field microscope (IX-81; Olympus) equipped with an MT20 Illumination system and a digital monochrome charge-coupled device camera (C9100; Hamamatsu Photonics). Olympus UPLSAPO ×10/0.4 NA, ×20/0.75 NA, and ×40/0.95 NA objectives were used. Images were acquired automatically and analyzed by the ScanR acquisition and analysis software (Olympus). Data were exported and analyzed further using Spotfire (TIBCO Software Inc.).

### siRNA screens

siRNA screens for regulators of Pol η foci formation were performed by transfecting U2OS/GFP-Pol η WT cells with siRNAs (10 nm final concentration) directly in 96-well SCREENSTAR Microplates (Greiner Bio-one), using HiPerFect transfection reagent (Qiagen) and an siRNA library targeting ubiquitin signaling factors (41) kindly provided by Claudia Lukas (Novo Nordisk Foundation Center for Protein Research, University of Copenhagen). After 48 h, cells were treated with 30 μm cisplatin for 6 h and subsequently grown overnight in fresh medium. Cells were then pre-extracted, fixed, and stained with 4′,6-diamidino-2-phenylindole. GFP-Pol η foci counts were determined using QIBC, and ScanR analysis software was used to calculate modified Z-scores, where the mean ± S.D. is replaced by the median and median absolute deviation, as previously described (64).

### Immunochemical methods

Immunoblotting and immunoprecipitation were done as previously described (65). To obtain chromatin-enriched fractions, cells were lysed in low-salt buffer (10 mm HEPES, pH 7.5, 10 mm KCl, 0.05% Nonidet P-40) and chromatin-associated proteins were extracted from the pellet by treatment with micrococcal nuclease in nuclease buffer (150 mm NaCl, 5 mm CaCl_2_). To prepare whole cell extracts, cells were lysed in EBC buffer (50 mm Tris, pH 7.5, 150 mm NaCl, 1 mm EDTA, 1 mm DTT, 0.5% Nonidet P-40, 1 mm DTT) supplemented with protease and phosphatase inhibitors. For detection of SUMOylated proteins, cells were lysed in denaturing buffer (20 mm Tris, pH 7.5, 50 mm NaCl, 1 mm EDTA, 1 mm DTT, 0.5% Nonidet P-40, 0.5% sodium deoxycholate, 0.5% SDS) containing protease and phosphatase inhibitors and sonicated. GFP immunoprecipitation was done using GFP-Trap-agarose beads (Chromotek). Antibodies used in this study included: Actin (M1501; Millipore), CPD (D194-1; MBL International), HA (F-7, sc-7392; Santa Cruz Biotechnology), MCM6 (C-20, sc-9843; Santa Cruz Biotechnology), PCNA (PC-10, sc-56; Santa Cruz Biotechnology), Pol η (A301–231A; Bethyl Laboratories), PIAS1 (ab77231; Abcam), PIAS4 (D2F12, 4392S; Cell Signaling Technology), RAD18 (ab57447; Abcam), SAE1 (A302-923A; Bethyl Laboratories), SUMO1 (ab32058; Abcam), SUMO2/3 (ab3742; Abcam), UBA2 (SAE2) (A302–925A; Bethyl Laboratories), and UBC9 (ab21193; Abcam). Rabbit polyclonal RNF4 antibody was a kind gift from Alfred Vertegaal (Leiden University Medical Center, Netherlands).

### Quantification of mRNA levels by RT-qPCR

RNA was isolated from cells (RNeasy kit, Qiagen) and cDNA was generated by PCR with reverse transcription (iScript cDNA Synthesis Kit, Bio-Rad) according to the manufacturer's instructions. Real-time quantitative PCR was performed using the Stratagene Mx3005P System and Brilliant III Ultra-Fast SYBR Green QPCR Master Mix (Agilent). GAPDH mRNA levels were used as a control for normalization. The following primers were used for amplification of the indicated cDNAs: GAPDH (forward), 5′-CAGAACATCATCCCTGCCTCTAC-3′; GADPH (reverse), 5′-TTGAAGTCAGAGGAGACCACCTG-3′; RNF111 (forward), 5′-TGCATCCTCACTTGGCCCAT-3′; RNF111 (reverse): 5′-TCAGTTCCTCAAAATTGCCCCTG-3′.

### Mass spectrometry

Analysis of Pol η SUMOylation sites was performed using the enhanced K0-SUMO strategy, as described (52). The Pol η SUMOylation sites reported here represent a subset of a larger overarching dataset, which was independently published previously (52). MS proteomics RAW data are available at the ProteomeXchange Consortium database via the Proteomics Identifications (PRIDE) partner repository (ID PXD004927). Briefly, HeLa and U2OS cell lines stably expressing K0-SUMO (His10-SUMO2–8KR-Q87R-IRES-GFP) were cultured and either mock treated or subjected to heat shock (43 °C for 1 h), proteasome inhibition by MG132 (10 μm, 8 h), or proteasome inhibition by Bortezomib (100 nm, 24 h). Experiments were performed in cell culture quadruplicates. Cells were lysed, after which SUMOylated peptides were purified as described (52), and trypsin-digested to yield peptides modified on lysine residues with the QQTGG SUMO2/3 mass remnant. Peptides were purified and fractionated into 6 fractions at high-pH on StageTips, prior to MS analysis.

All samples were analyzed on 15-cm long 75-μm internal diameter columns, packed in-house with ReproSil-Pur 120 C18-AQ 1.9-μm beads (Dr. Maisch), connected to an EASY-nLC 1200 system (Thermo). Fractionated samples were eluted over 70-min analytical gradients increasing from 2 to 35% acetonitrile in 0.1% fatty acid. The column was heated to 40 °C, and samples were ionized using a Nanospray Flex Ion Source (Thermo) and analyzed using a Q-Exactive HF mass spectrometer (Thermo). Spray voltage was set to 2 kV, capillary temperature set to 275 °C, and S-Lens RF level set to 50%. For full scans, a resolution setting of 60,000, an AGC target of 3,000,000, a maximum injection time of 50 ms, and a scan range of 300–1,750 *m*/*z* was used. Precursors were fragmented using higher-energy collision disassociation using a normalized collision energy of 25, with the AGC target set at 100,000, and a precursor isolation width of 1.3 *m*/*z*. Unassigned charges, and charges 1 and 7+, were excluded. A dynamic exclusion of 30 s was used. MS2-level settings included a loop count of 7, resolution of 60,000, maximum injection time of 120 ms, and an underfill ratio of 6% (50,000 intensity threshold).

All RAW files were analyzed in a single computational run using MaxQuant software version 1.5.3.30. Default MaxQuant settings were used for data analysis, with exceptions and noteworthy default settings described below. For generation of the theoretical peptide library, a FASTA database containing all human proteins was downloaded from UniProt on 28 July 2016, which contained 92,607 unique protein entries. Trypsin was defined as the proteolytic enzyme (default), maximum missed cleavages were set to 4, and maximum number of variable modifications per peptide was set to 4, with the following variable modifications: protein N-terminal acetylation (default), methionine oxidation (default), QQTGG on lysine (52), pyroQQTGG on lysine (52), and phosphorylation on serine, threonine, and tyrosine. Minimum peptide length was set to 6, maximum peptide mass was set to 6,000. Data were automatically filtered at the peptide-spectrum-match, protein, and site-identification levels by posterior error probability to yield a false discovery rate of <1% (default), an MS1-level (precursor) mass tolerance of 4.5 ppm was applied (default), an MS2-level (fragment) mass tolerance of 20 ppm was applied (default), and modified peptides were filtered for an Andromeda score of >40 (default) and a delta score of >6 (default). In addition, the SUMO site output of MaxQuant was manually filtered for a localization delta score of >6, the presence of QQTGG mass remnant fragments (diagnostic peaks) in the MS/MS spectra, and the absence of a reversed database hit.

### Clonogenic survival assays

For colony formation assays, cells were transfected with siRNAs, plated at low densities, and treated with UV at the indicated doses, and subsequently incubated for 9 days in medium supplemented with 0.4 mm caffeine. Cells were then fixed and stained with crystal violet (0.5% (w/v) crystal violet, 25% (v/v) methanol). Colonies were counted using an automated colony counter (GelCount; Oxford Optronix). The surviving fraction was calculated as number of colonies/(number of seeded cells × plating efficiency) and normalized to the mock control.

## Data availability

Mass spectrometry proteomics RAW data are available at the ProteomeXchange Consortium database via the Proteomics Identifications (PRIDE) partner repository under dataset ID PXD004927. All other data supporting the findings of this study are available within the article and supporting information.

## Supplementary Material

Supporting Information

## Footnotes

3 C. Guerillon, S. Smedegaard, I. A. Hendriks, M. L. Nielsen, and N. Mailand, unpublished observations.
