# Supplementary material for: Multisite SUMOylation restrains DNA polymerase η interactions with DNA damage sites
Source: J Biol Chem. 2020 Apr 29;295(25):8350–62. doi: 10.1074/jbc.RA120.013780 (PMC7307195; doi:10.1074/jbc.RA120.013780)
Supplement: Supporting Information [file supp_295_25_8350__index.html]

Multisite SUMOylation restrains DNA polymerase η interactions with DNA damage sites — SUMOylation limits Pol η interaction with DNA damage sites — Supporting Information 

# Multisite SUMOylation restrains DNA polymerase η interactions with DNA damage sites

## Supporting Information

- Supporting Information - Table S1
- Supporting Information - Table S2
- Supporting Information - Table S3
- Supporting Information - Figure S1-S4
