# Supplementary material for: Multisite SUMOylation restrains DNA polymerase η interactions with DNA damage sites
Source: J Biol Chem. 2020 Apr 29;295(25):8350–62. doi: 10.1074/jbc.RA120.013780 (PMC7307195; doi:10.1074/jbc.RA120.013780)
Supplement: Supporting Information [file supp_RA120.013780_160008_1_supp_518709_q9cls7.pdf]

Supporting Information for

Multi-site SUMOylation restrains DNA Polymerase η interactions with DNA damage sites

Claire Guérillon[1], Stine Smedegaard[1], Ivo A. Hendriks[2], Michael L. Nielsen[2] and Niels Mailand[1,*]

[1]Ubiquitin Signaling Group, Protein Signaling Program, Novo Nordisk Foundation Center for Protein Research, Faculty of Health and Medical Sciences, University of Copenhagen, Blegdamsvej 3B, DK-2200 Copenhagen, Denmark.

[2]Proteomics Program, Novo Nordisk Foundation Center for Protein Research, Faculty of Health and Medical Sciences, University of Copenhagen, Blegdamsvej 3B, DK-2200 Copenhagen, Denmark

Table S1.
Results of high-throughput microscopy screen for regulators of Pol η interaction with DNA damage sites
U2OS/GFP-Pol η cells were transfected with an siRNA library targeting ubiquitin and UBL signaling factors (1251 genes, two siRNAs per gene). After 72 h, cells were treated with 30 μM Cisplatin for 6 h and subsequently grown overnight in fresh media. Cells were then pre-extracted, fixed and stained with DAPI. GFP-Pol η foci counts were determined using QIBC. Table shows modified Z-scores for all siRNAs in the library.

Table S2.
List of validated Ub/UBL signaling factors whose knockdown leads to enhanced formation of DNA damage-induced Pol η foci

Table S3.
Overview of endogenous Pol η SUMOylation sites identified by mass spectrometry

Figure S1.
SUMO-dependent regulation of Pol η association with DNA damage sites.
A. Immunoblot analysis of whole-cell extracts (WCE) of U2OS cells transfected with non-targeting control (CTRL) siRNA or siRNAs targeting indicated SUMO enzymes. Expression levels of SAE1, UBA2 and UBC9 (normalized to MCM6 expression) were quantified using ImageJ. **B**. Results of validation screen. The sum of GFP-Pol η foci intensity in U2OS/GFP-Pol η cells transfected with indicated siRNAs, exposed to cisplatin for 6 h and fixed 16 h later was quantified using QIBC analysis (mean±SD; ≥294 cells quantified per condition). **C**. Results of validation screen. The sum of GFP-Pol η foci intensity at different time points after exposure to cisplatin in cells treated as in (B) was quantified using QIBC analysis (mean±SD; ≥3,000 cells quantified per condition). **D**. U2OS/GFP-Pol η cells were treated or not with SUMO E1 inhibitor (SUMOi) for 30 min, exposed to UV and fixed 6 h later. GFP-Pol η foci count per nucleus was quantified by QIBC analysis (mean±SEM; *n*=3 independent experiments; ≥7,482 cells quantified per condition).

Guérillon et al., Figure S1

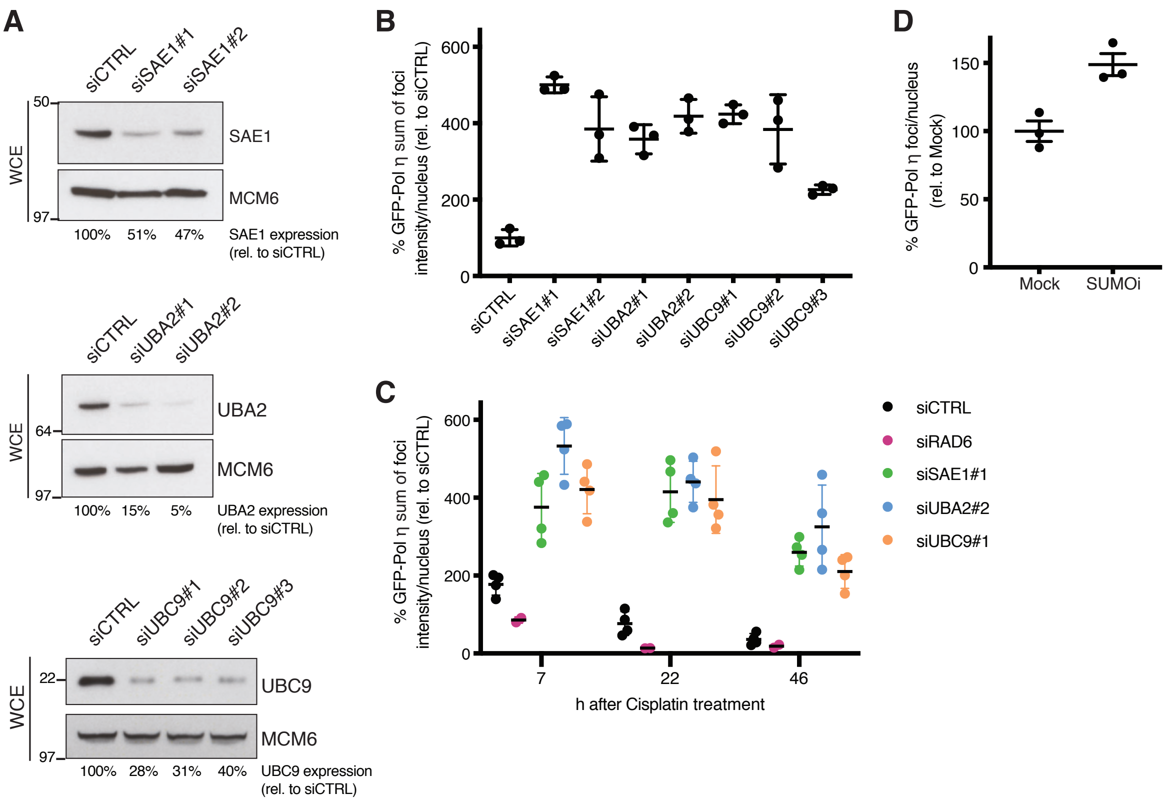

Figure S2.

Impact of removing SUMO modification components on cell cycle distribution, PCNA monoubiquitylation and Pol η SUMOylation

A. U2OS cells transfected with indicated siRNAs were pulse-labeled with EdU, fixed and processed for EdU labeling and staining of total DNA with DAPI. Cell cycle profiles were analyzed using QIBC (≥1869 cells quantified per condition). Percentage of cells in different cell cycle stages (G1 phase, blue; S phase, pink; G2/M phase, green) is indicated. **B**. Immunoblot analysis of chromatin-enriched fractions of U2OS cells transfected with indicated siRNAs and exposed to UV. **C**. U2OS left untreated or transfected with plasmids encoding the indicated GFP-Pol η alleles and exposed to UV were lysed and subjected to GFP immunoprecipitation (IP) under denaturing conditions, followed by immunoblotting with SUMO2/3, GFP and MCM6 (loading control) antibodies. **D**. Quantification of GFP-Pol η foci formation in U2OS/GFP-Pol η collected at indicated times after exposure to UV (mean±SEM; *n*=2 independent experiments; ≥508 cells quantified per condition). **E**. U2OS/GFP-Pol η cells transfected with indicated siRNAs and exposed to UV were lysed and subjected to GFP immunoprecipitation (IP) under denaturing conditions, followed by immunoblotting with SUMO2/3, GFP and MCM6 (loading control) antibodies. **F**. Cell cycle profiles of cells transfected with indicated PIAS1 siRNAs or non-targeting control (CTRL) siRNA were determined as in (A) (≥2936 cells quantified per condition).

Guérillon et al., Figure S2

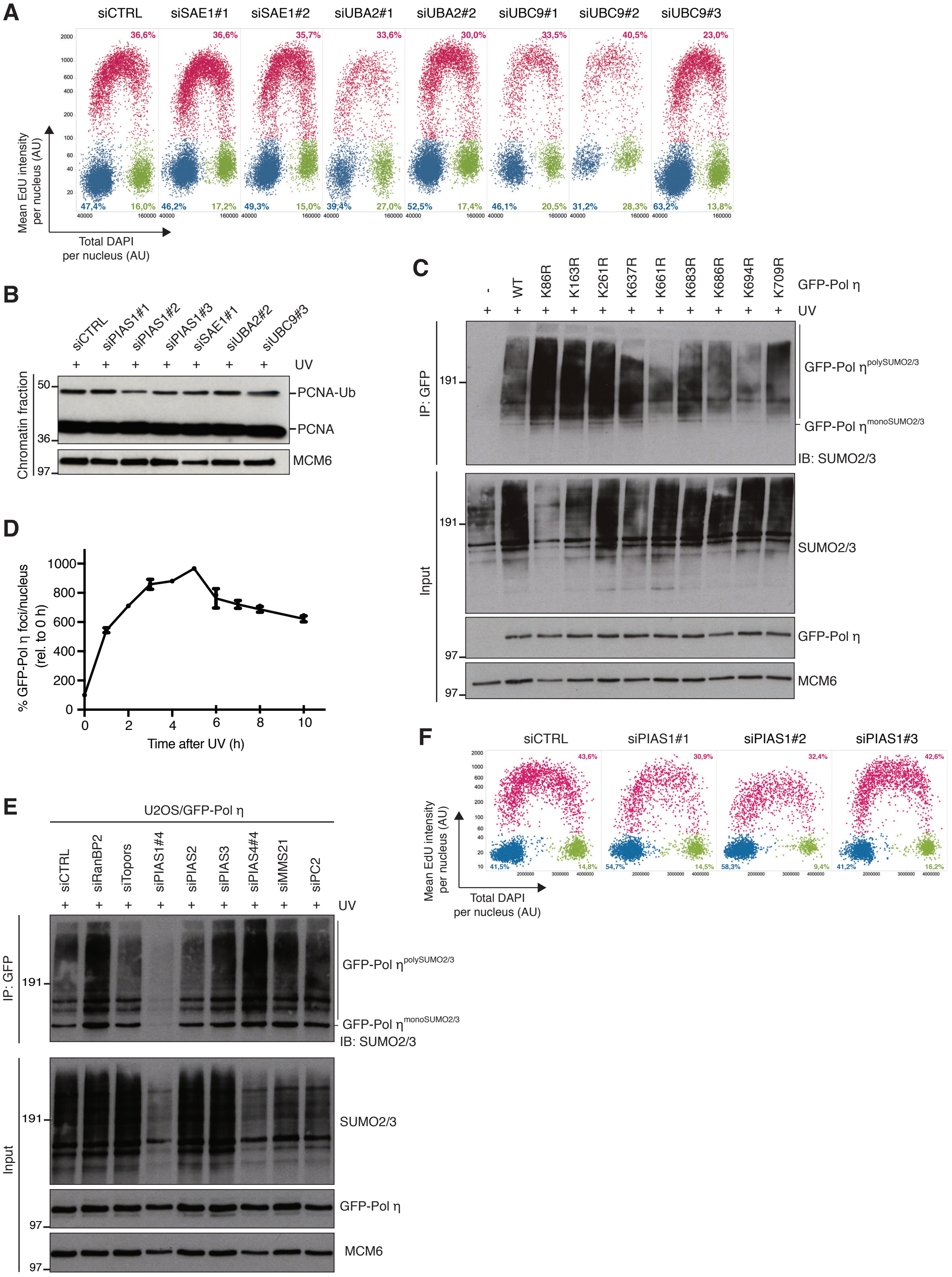

Figure S3.
Impact of impaired SUMO-dependent processing of Pol η on its association with DNA damage sites and TLS functionality

A. Quantification of the sum of GFP-Pol η foci intensity using QIBC analysis in U2OS/GFP-Pol η cells transfected with indicated siRNAs, exposed to UV and fixed 6 h later (mean±SEM; *n*=2 independent experiments; ≥1991 cells quantified per condition). **B**. Immunoblot analysis of U2OS cells transfected with non-targeting control (CTRL) or RNF4 siRNAs using indicated antibodies. **C**. qPCR analysis of RNF111 mRNA levels in U2OS cells transfected with indicated siRNAs relative to siCTRL. Primers to GAPDH were used as a normalization control (mean±SEM; *n*=2 independent experiments). **D**. Quantification of the sum of GFP-Pol η foci intensity using QIBC analysis in U2OS/GFP-Pol η cells transfected with indicated siRNAs and treated as in (A) (mean±SEM; *n*=4 independent experiments; ≥1254 cells quantified per condition). **E**. U2OS cell lines stably expressing GFP-Pol η WT or 20KR mutant (clone 1 (c1) and clone 2 (c2)) were transfected or not with siRNA targeting the 3'UTR of endogenous Pol η (siPol η#2), exposed to UV (5 J/m^2) and fixed 6 h later. Cells were then stained with antibody specific to cyclobutane pyrimidine dimers (CPDs) under native conditions, where only CPDs in single-stranded DNA (ssDNA) are exposed, and CPD intensity per nucleus was quantified by QIBC analysis (mean±SEM; *n*=2 independent experiments; ≥10,086 cells quantified per condition). Both GFP-Pol η WT or 20KR are able to rescue the increased occurrence of CPDs in ssDNA resulting from knockdown of endogenous Pol η, indicating that both alleles are proficient for TLS-mediated bypass of these lesions. **F**. Clonogenic survival of U2OS cells and derivative stable U2OS/NLS-GFP-Pol η WT and U2OS/NLS-GFP-Pol η 20KR mutant (c1) cell lines transfected with siCTRL or siPol η#2 and exposed to the indicated doses of UV. After UV radiation the medium was supplemented with 0,4 mM caffeine (mean±SEM; *n*=2 experiments).

Guérillon et al., Figure S3

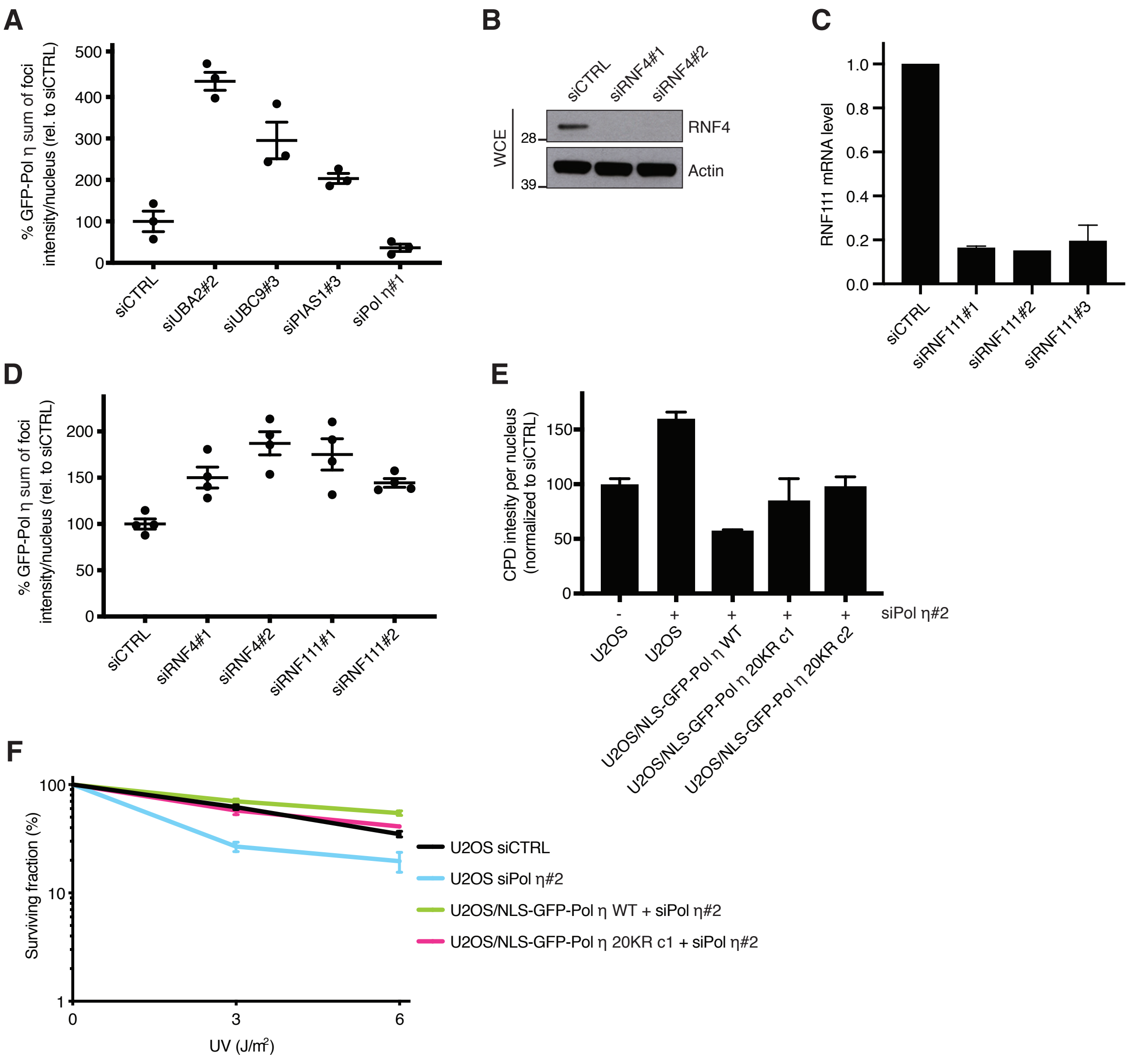

Figure S4.
MS spectra for identified SUMO sites in human Pol η

A collection of 19 fully annotated MS/MS spectra corresponding to all POLH SUMO sites reported in this work. Both “Standard” and “Advanced” annotations via the built-in MaxQuant Viewer software are available for each spectrum. Note that “qq” in the peptide sequence corresponds to attachment of the SUMO C-terminal mass remnant, QQTGG, covalently linked to the lysine residue via its C-terminal glycine residue.

Raw file	Scan	Method	Score	m/z	Gene names
QE6_LC6_IAH_SUMO_HeLa_M_3_F5	25646	FTMS; HCD	208.28	633.02	POLH

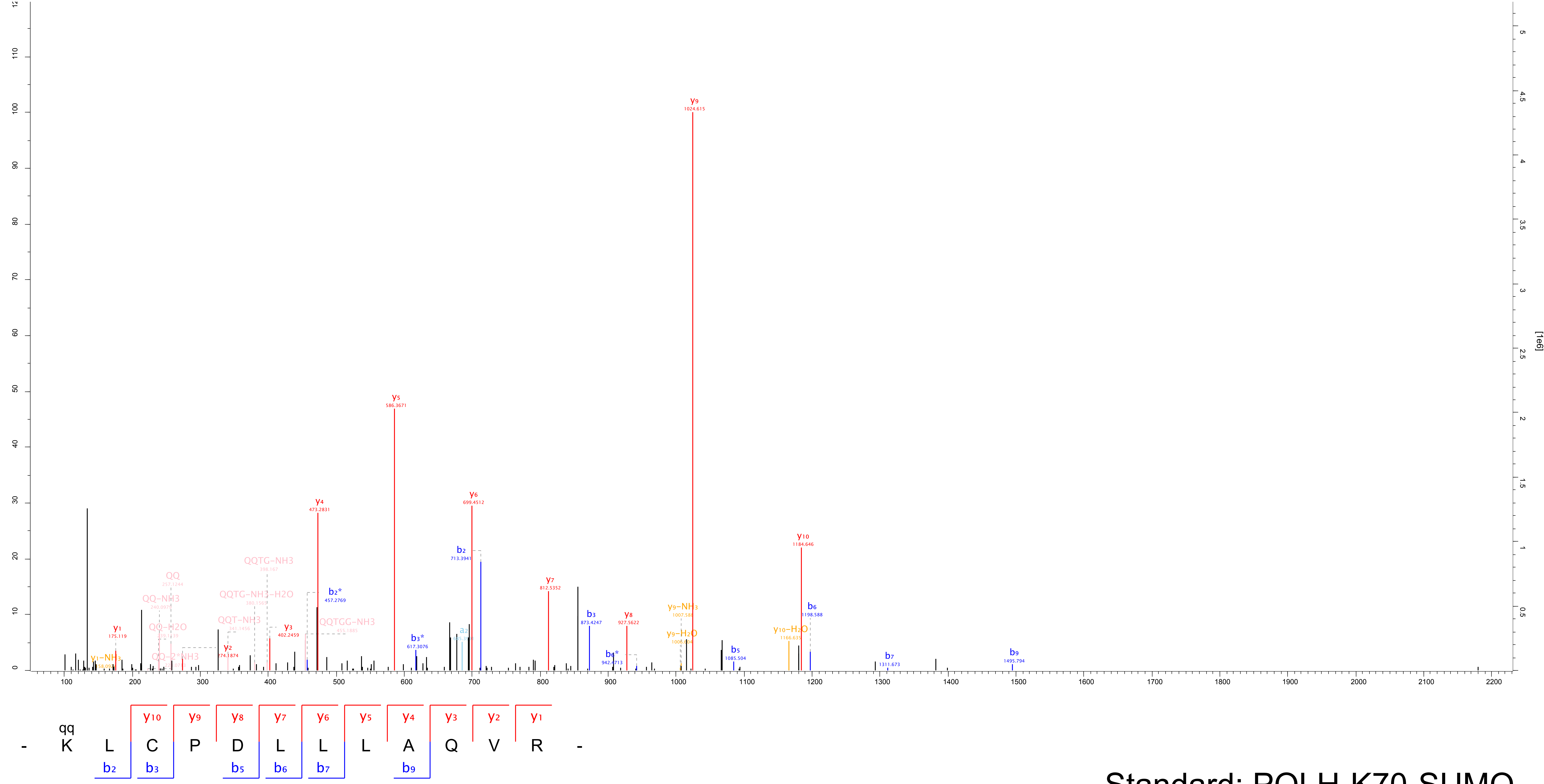

Standard: POLH-K70-SUMO

Raw file	Scan	Method	Score	m/z	Gene names
QE6_LC6_IAH_SUMO_HeLa_M_3_F5	25646	FTMS; HCD	208.28	633.02	POLH

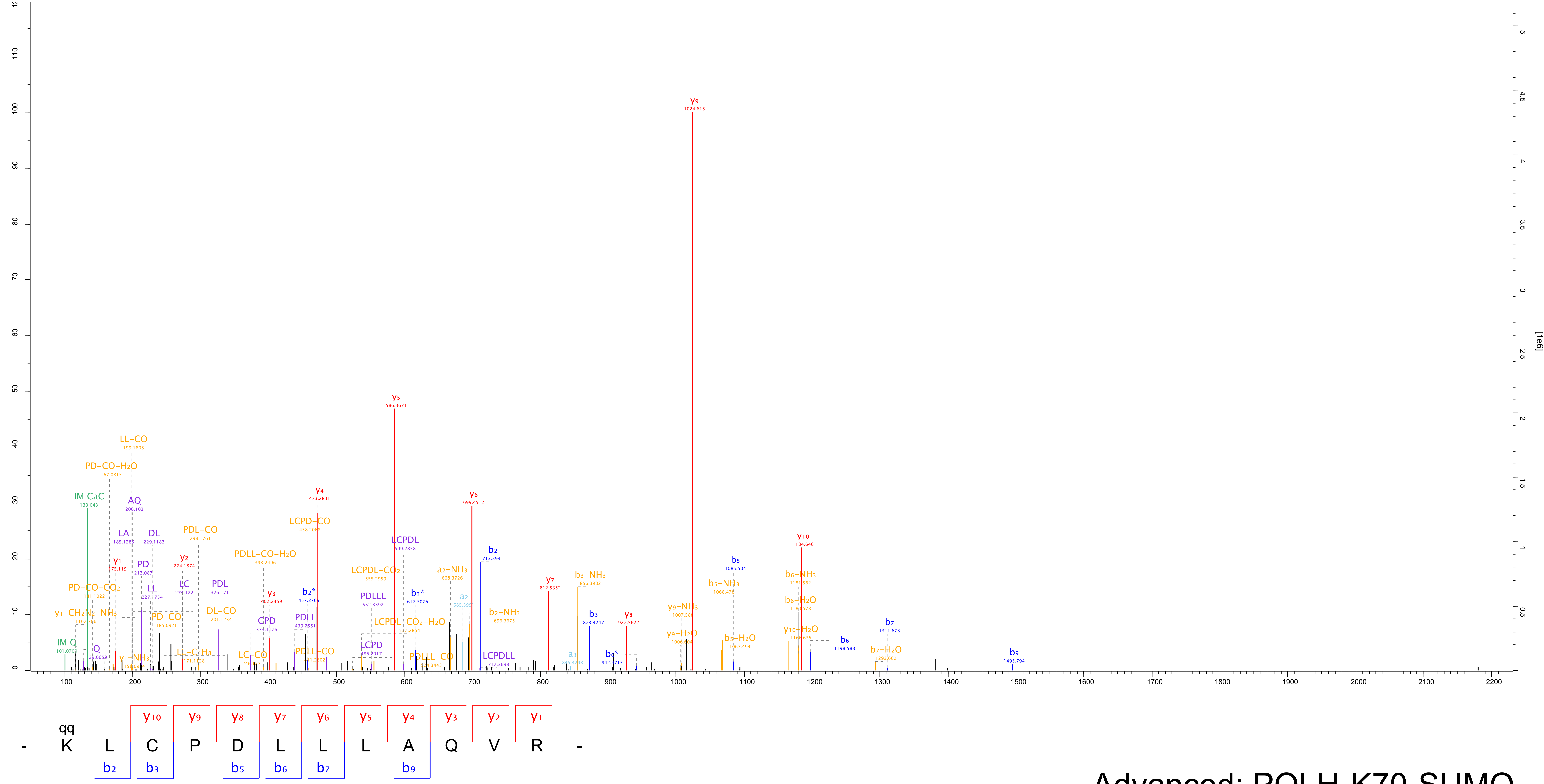

Advanced: POLH-K70-SUMO

Raw file	Scan	Method	Score	m/z	Gene names
QE6_LC6_IAH_SUMO_HeLa_M_2_F5	21588	FTMS; HCD	237.01	1408.74	POLH

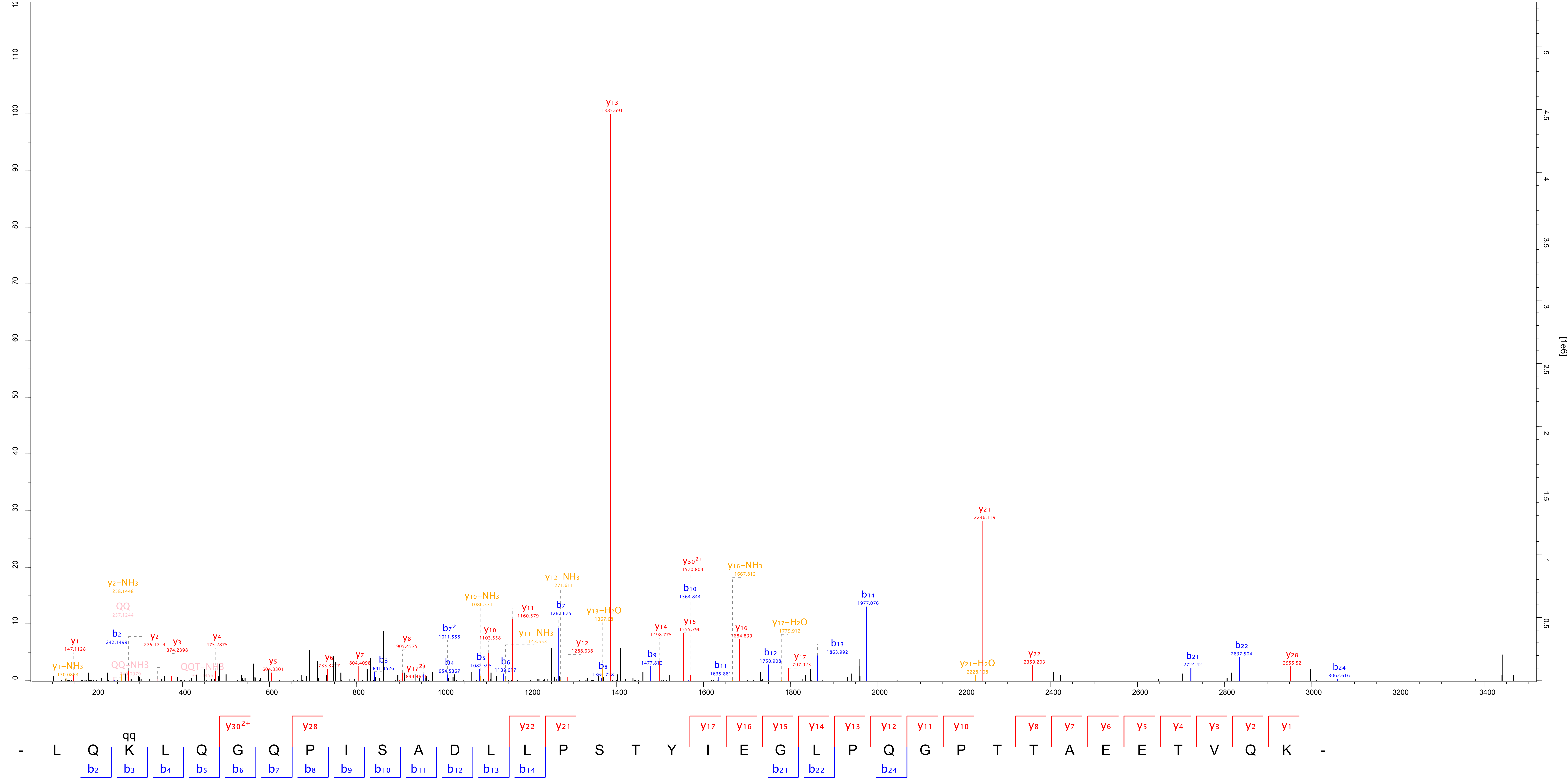

Standard: POLH-K131-SUMO

Raw file	Scan	Method	Score	m/z	Gene names
QE6_LC6_IAH_SUMO_HeLa_M_2_F5	21588	FTMS; HCD	237.01	1408.74	POLH

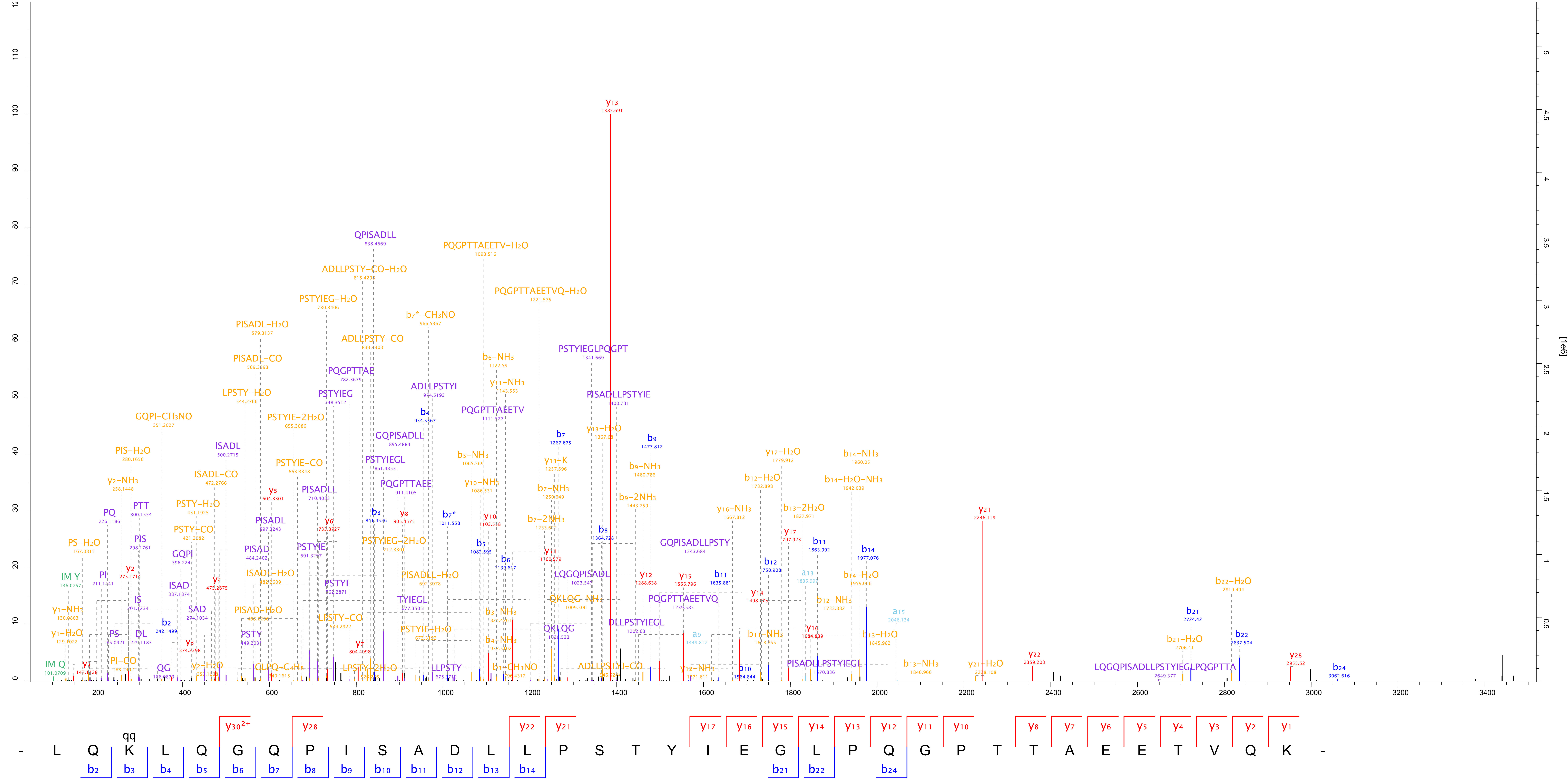

Advanced: POLH-K131-SUMO

Raw file	Scan	Method	Score	m/z	Gene names
QE6_LC6_IAH_SUMO_HeLa_M_1_F6	7344	FTMS; HCD	112.03	507.03	POLH

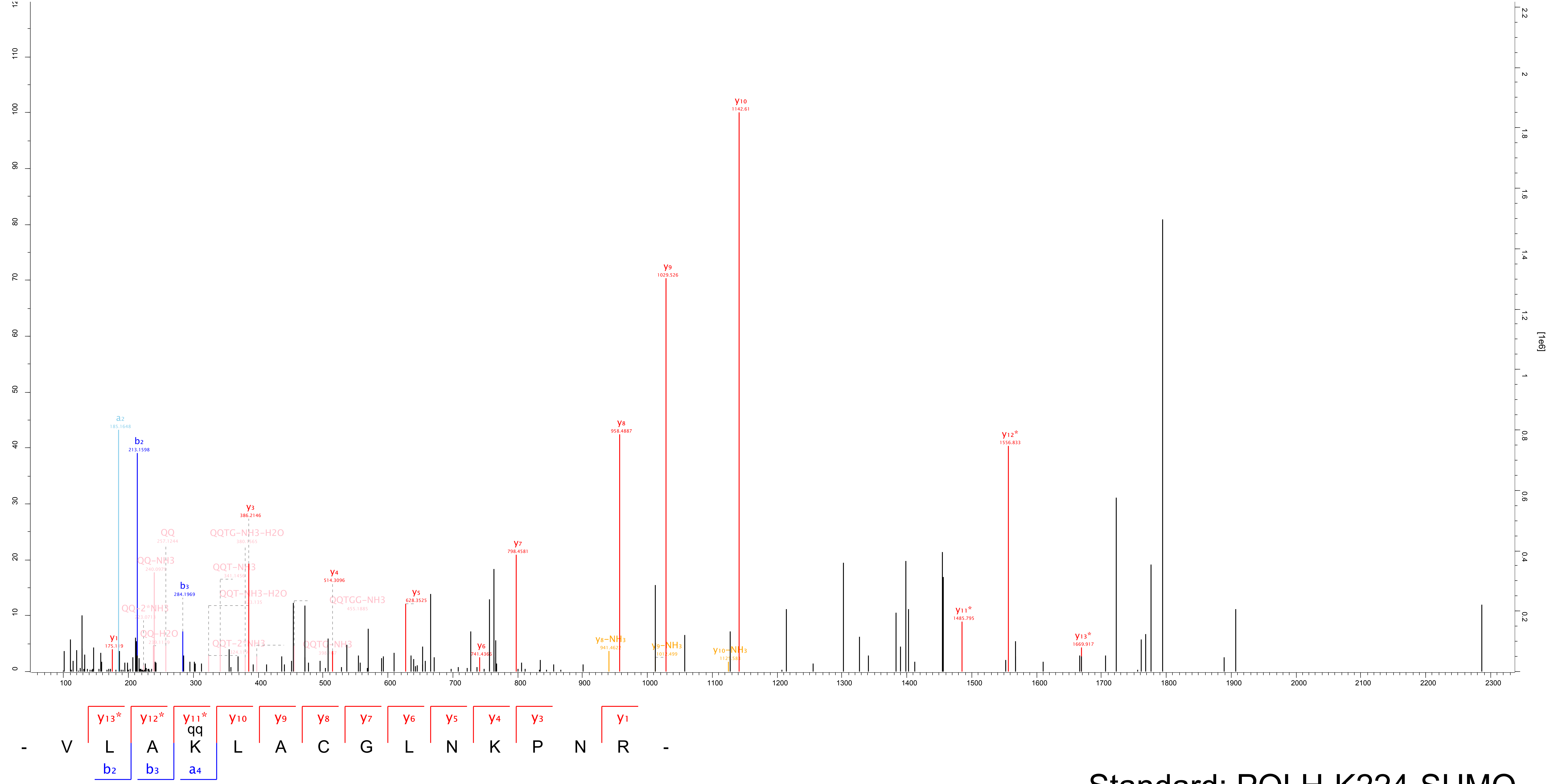

Standard: POLH-K224-SUMO

Raw file	Scan	Method	Score	m/z	Gene names
QE6_LC6_IAH_SUMO_HeLa_M_1_F6	7344	FTMS; HCD	112.03	507.03	POLH

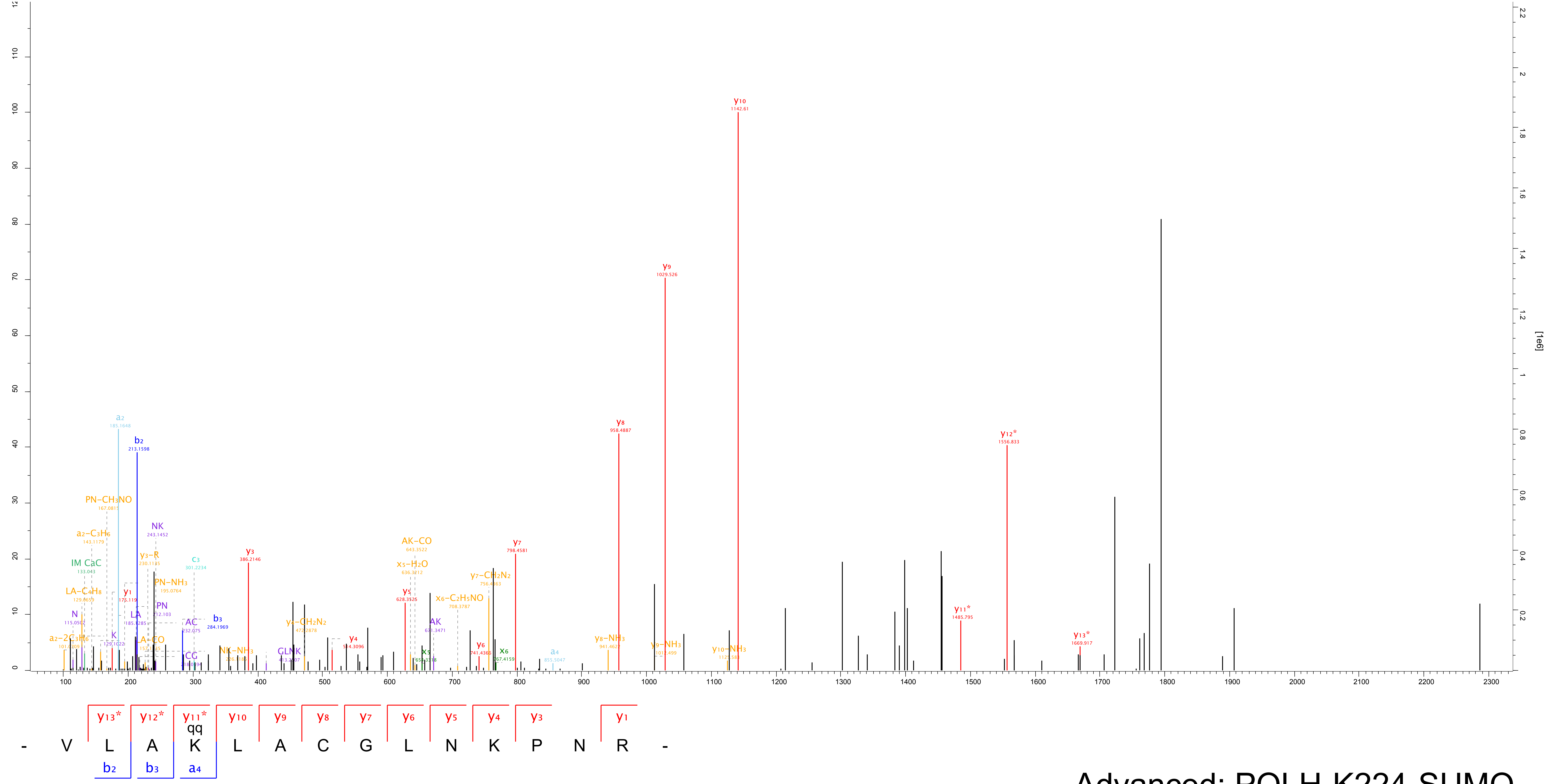

Advanced: POLH-K224-SUMO

Raw file	Scan	Method	Score	m/z	Gene names
QE6_LC6_IAH_SUMO_HeLa_M_2_F5	4216	FTMS; HCD	105.38	538.61	POLH

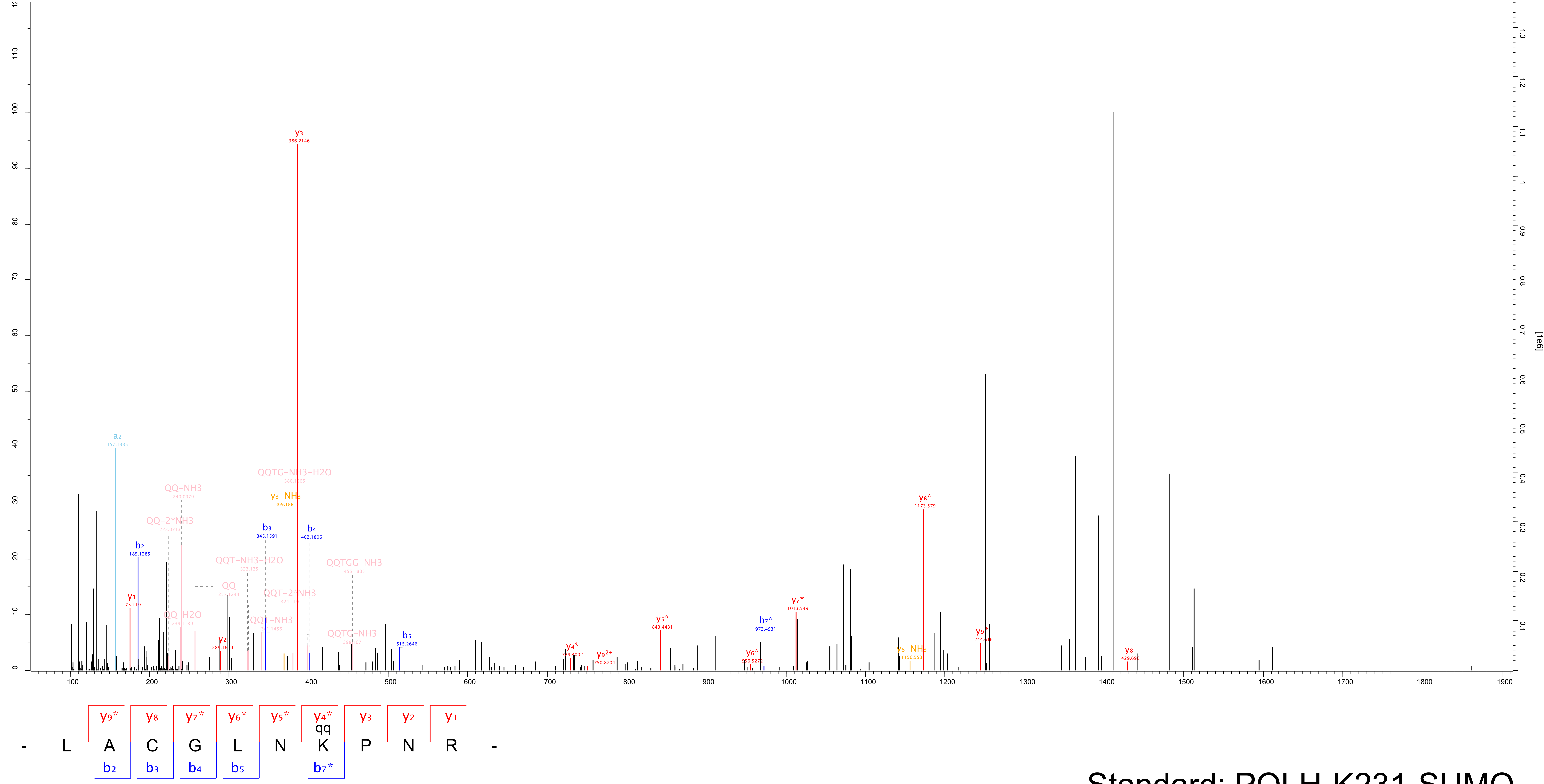

Standard: POLH-K231-SUMO

Raw file	Scan	Method	Score	m/z	Gene names
QE6_LC6_IAH_SUMO_HeLa_M_2_F5	4216	FTMS; HCD	105.38	538.61	POLH

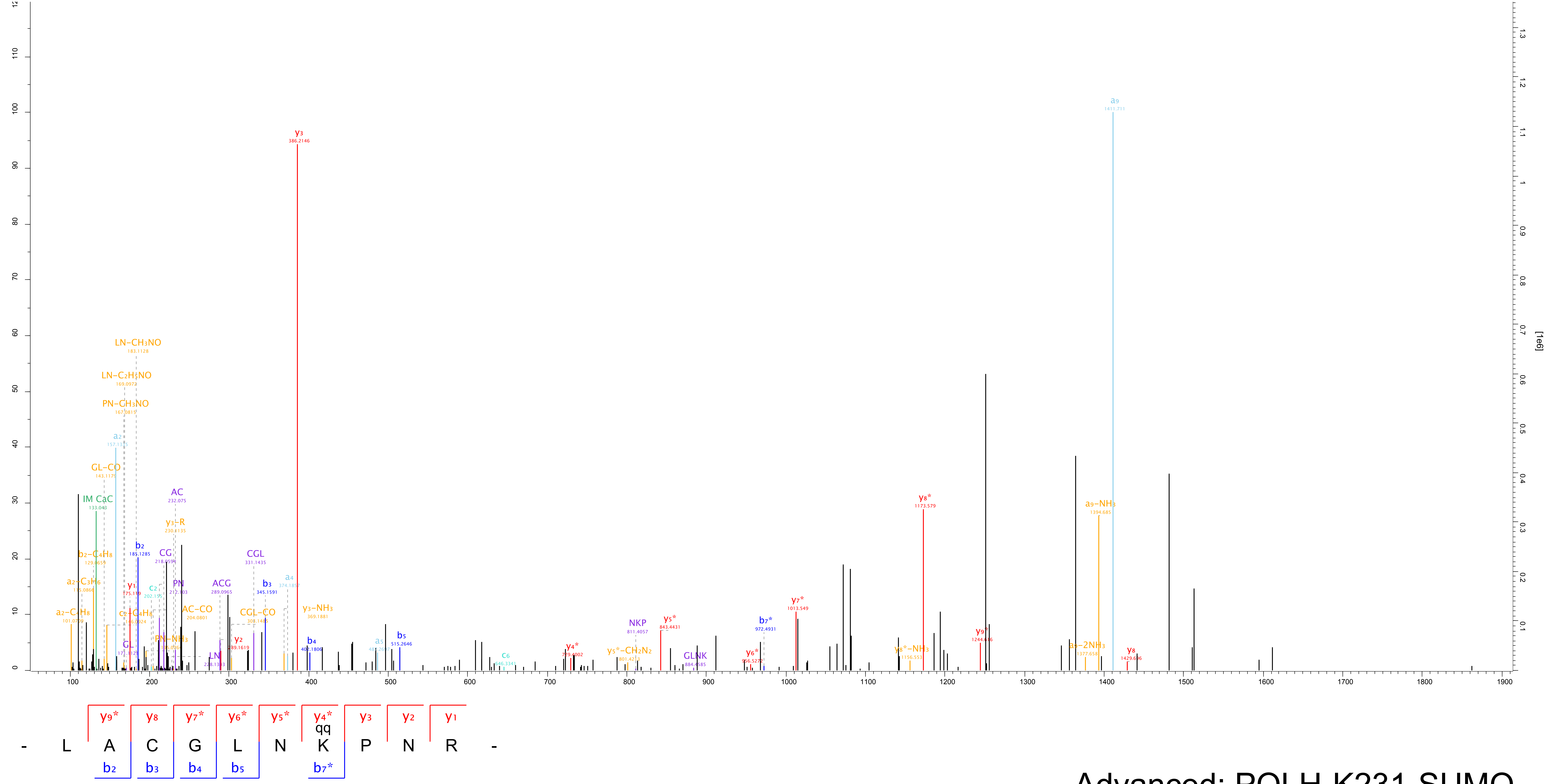

Advanced: POLH-K231-SUMO

Raw file	Scan	Method	Score	m/z	Gene names
QE6_LC6_IAH_SUMO_U2OS_M_2_F1	7391	FTMS; HCD	114.79	540.28	POLH

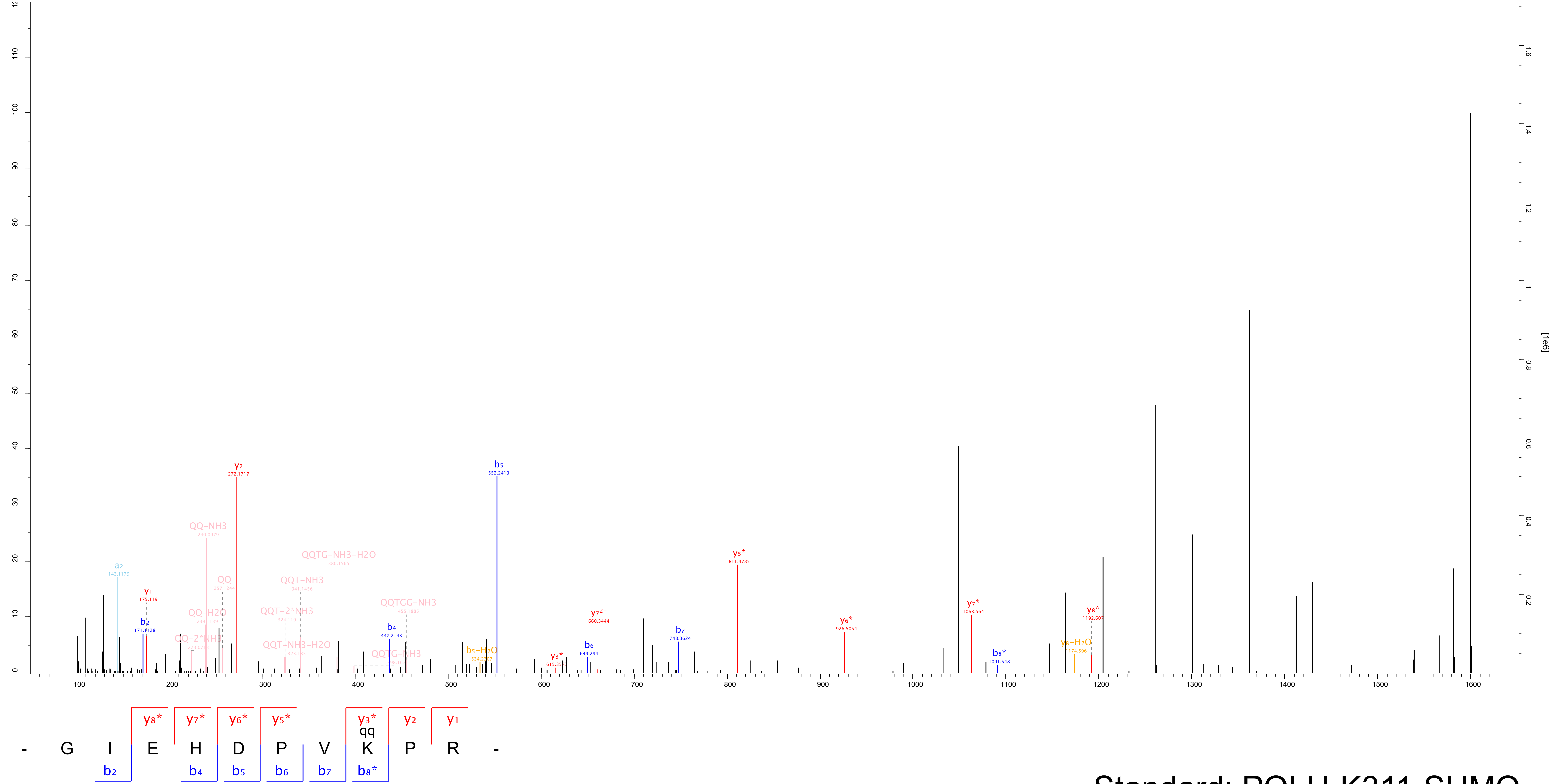

Standard: POLH-K311-SUMO

Raw file	Scan	Method	Score	m/z	Gene names
QE6_LC6_IAH_SUMO_U2OS_M_2_F1	7391	FTMS; HCD	114.79	540.28	POLH

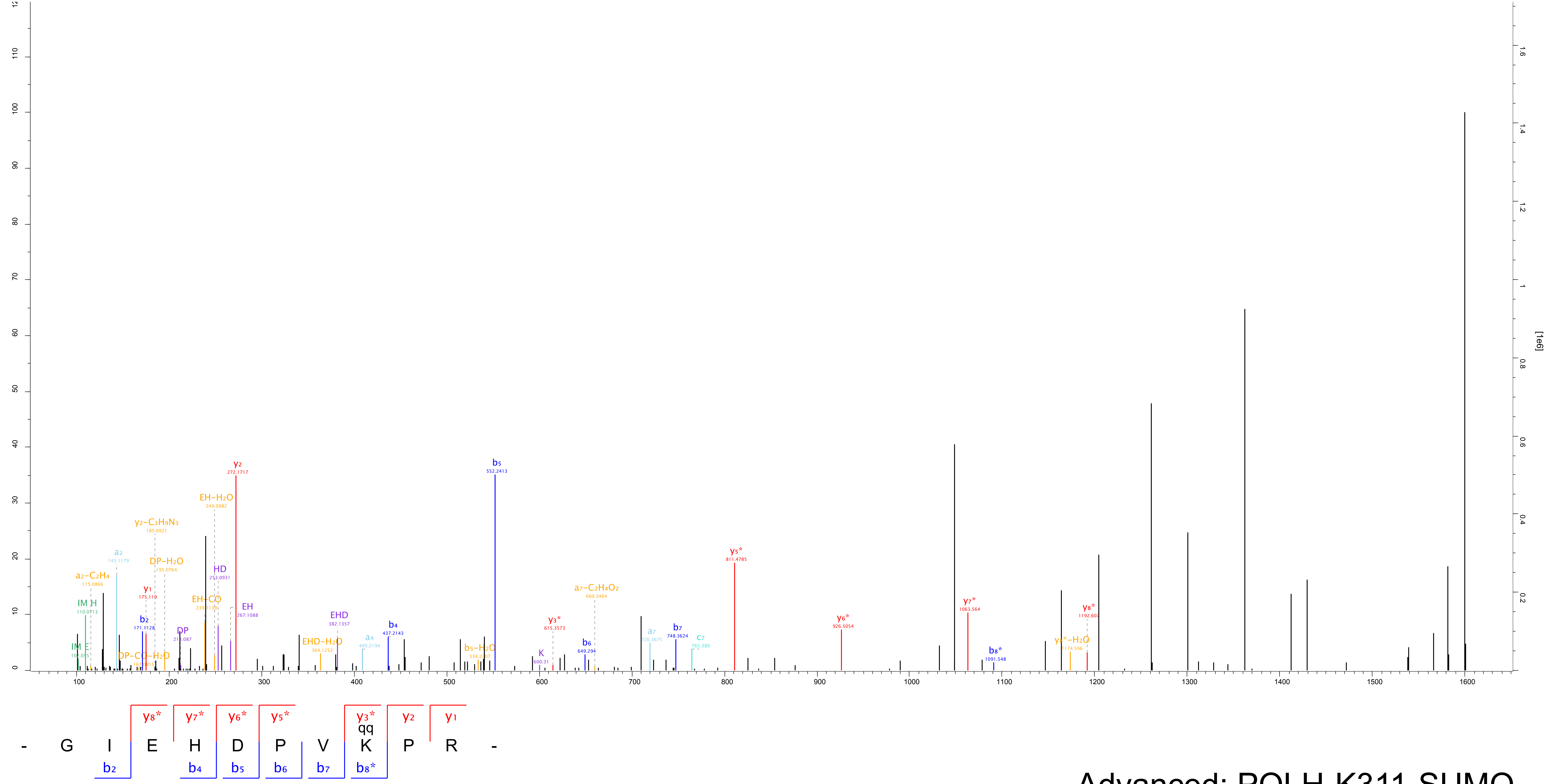

Advanced: POLH-K311-SUMO

Raw file	Scan	Method	Score	m/z	Gene names
QE6_LC6_IAH_SUMO_U2OS_M_1_F5	7353	FTMS; HCD	93.48	549.63	POLH

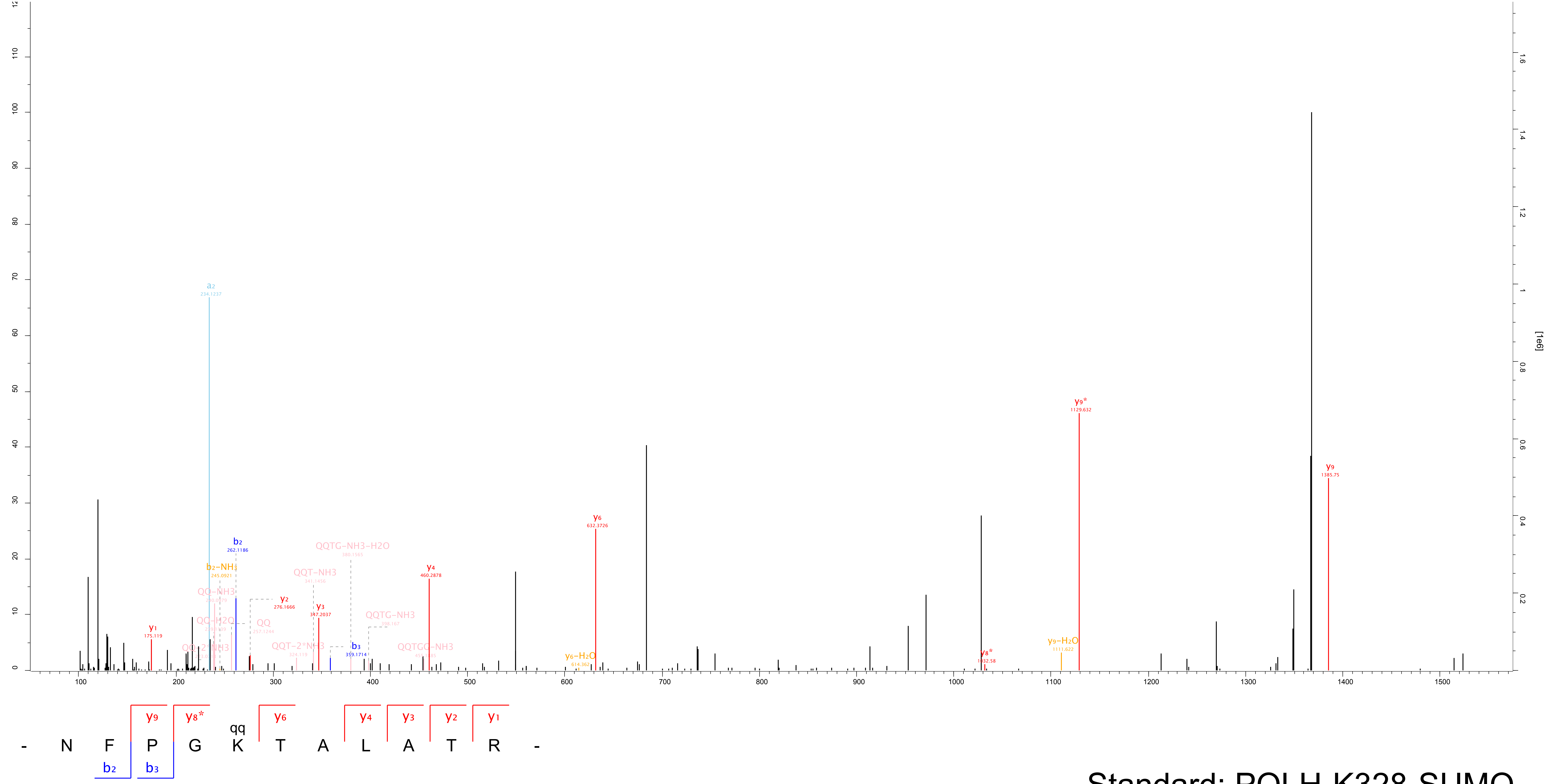

Standard: POLH-K328-SUMO

Raw file	Scan	Method	Score	m/z	Gene names
QE6_LC6_IAH_SUMO_U2OS_M_1_F5	7353	FTMS; HCD	93.48	549.63	POLH

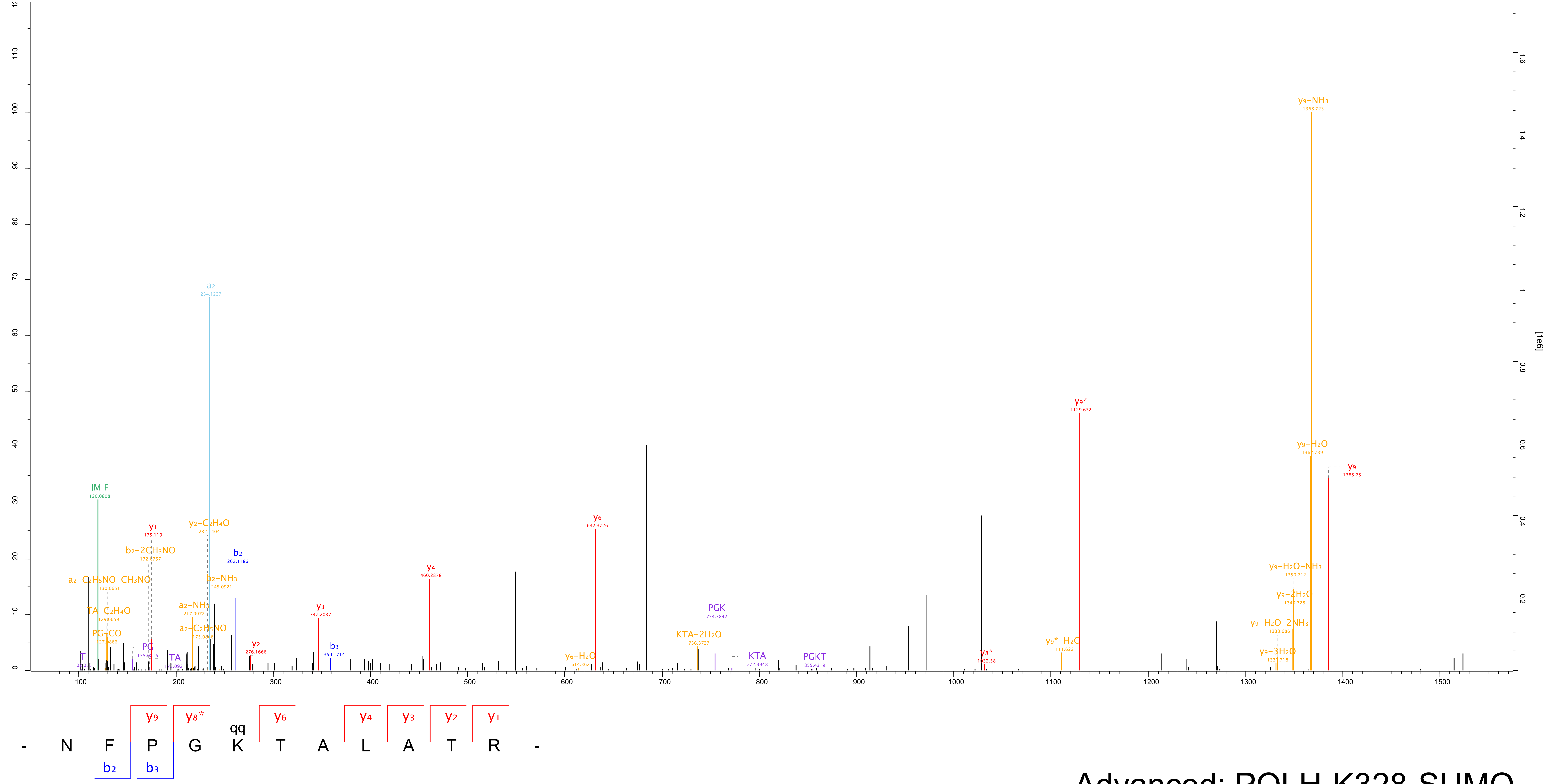

Advanced: POLH-K328-SUMO

Raw file	Scan	Method	Score	m/z	Gene names
QE6_LC6_IAH_SUMO_HeLa_M_3_F5	31361	FTMS; HCD	47.99	1296.64	POLH

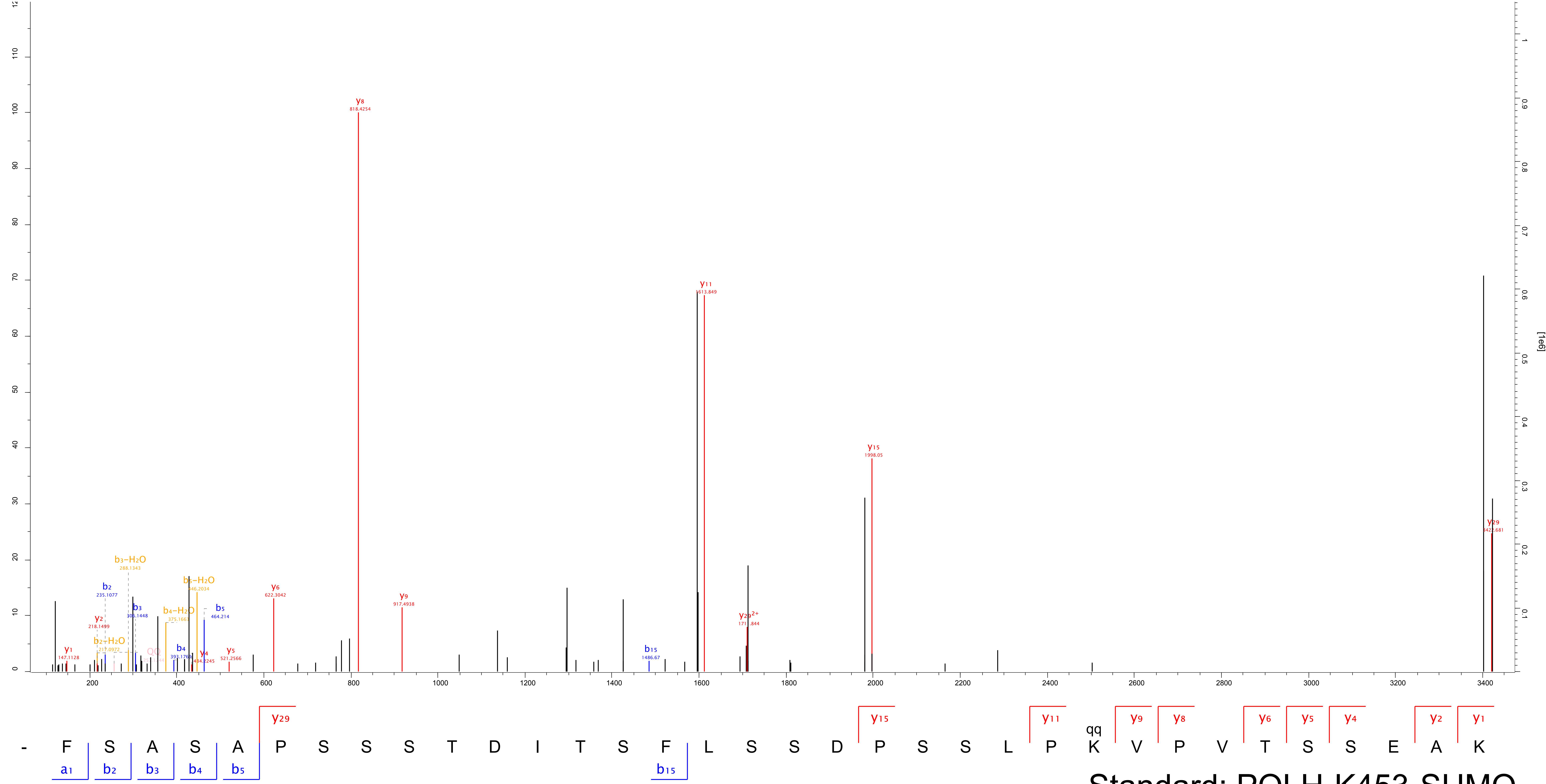

Standard: POLH-K453-SUMO

Raw file	Scan	Method	Score	m/z	Gene names
QE6_LC6_IAH_SUMO_HeLa_M_3_F5	31361	FTMS; HCD	47.99	1296.64	POLH

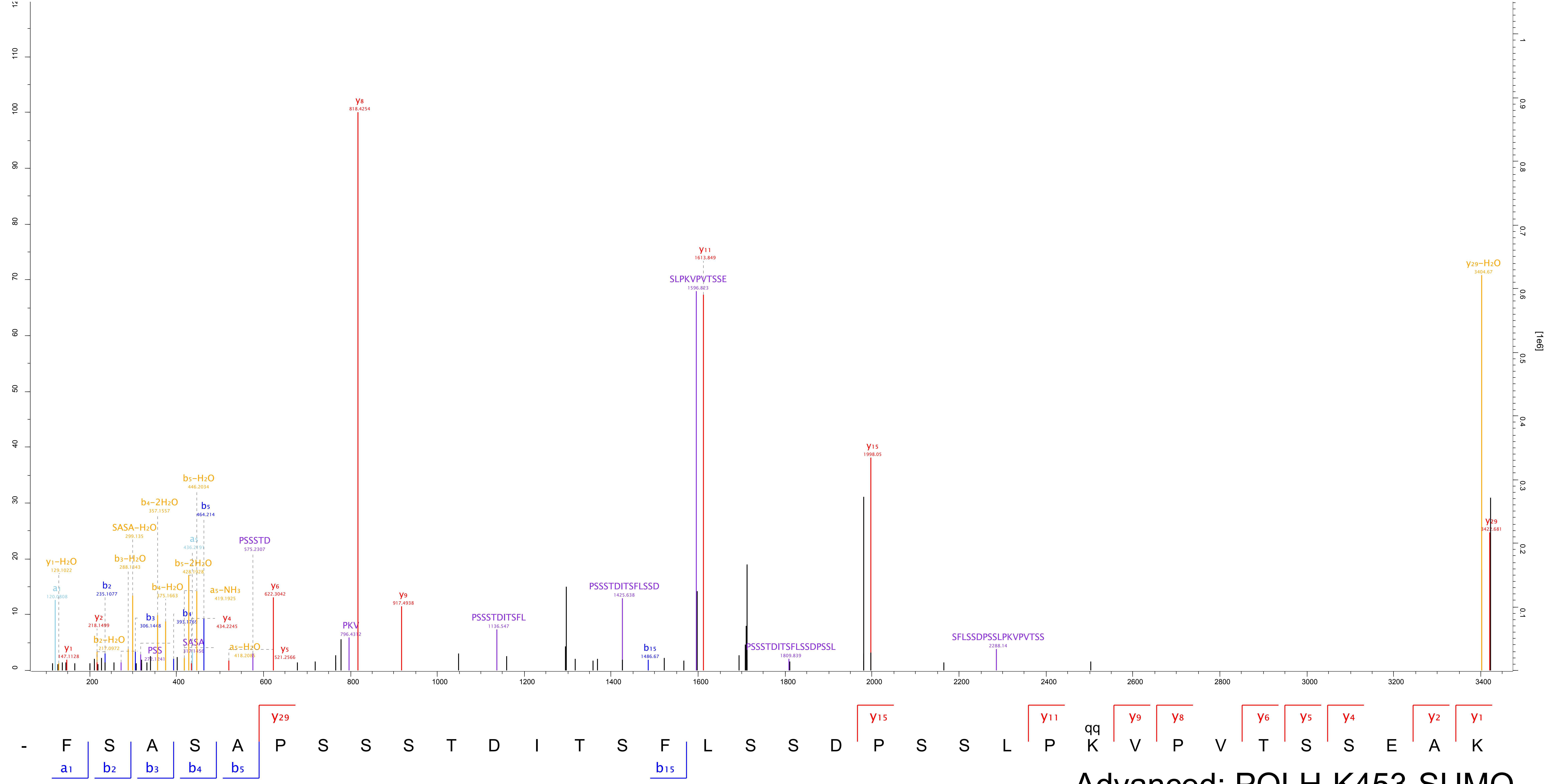

Advanced: POLH-K453-SUMO

Raw file	Scan	Method	Score	m/z	Gene names
QE6_LC6_IAH_SUMO_HeLa_M_1_F3	8492	FTMS; HCD	324.12	830.1	POLH

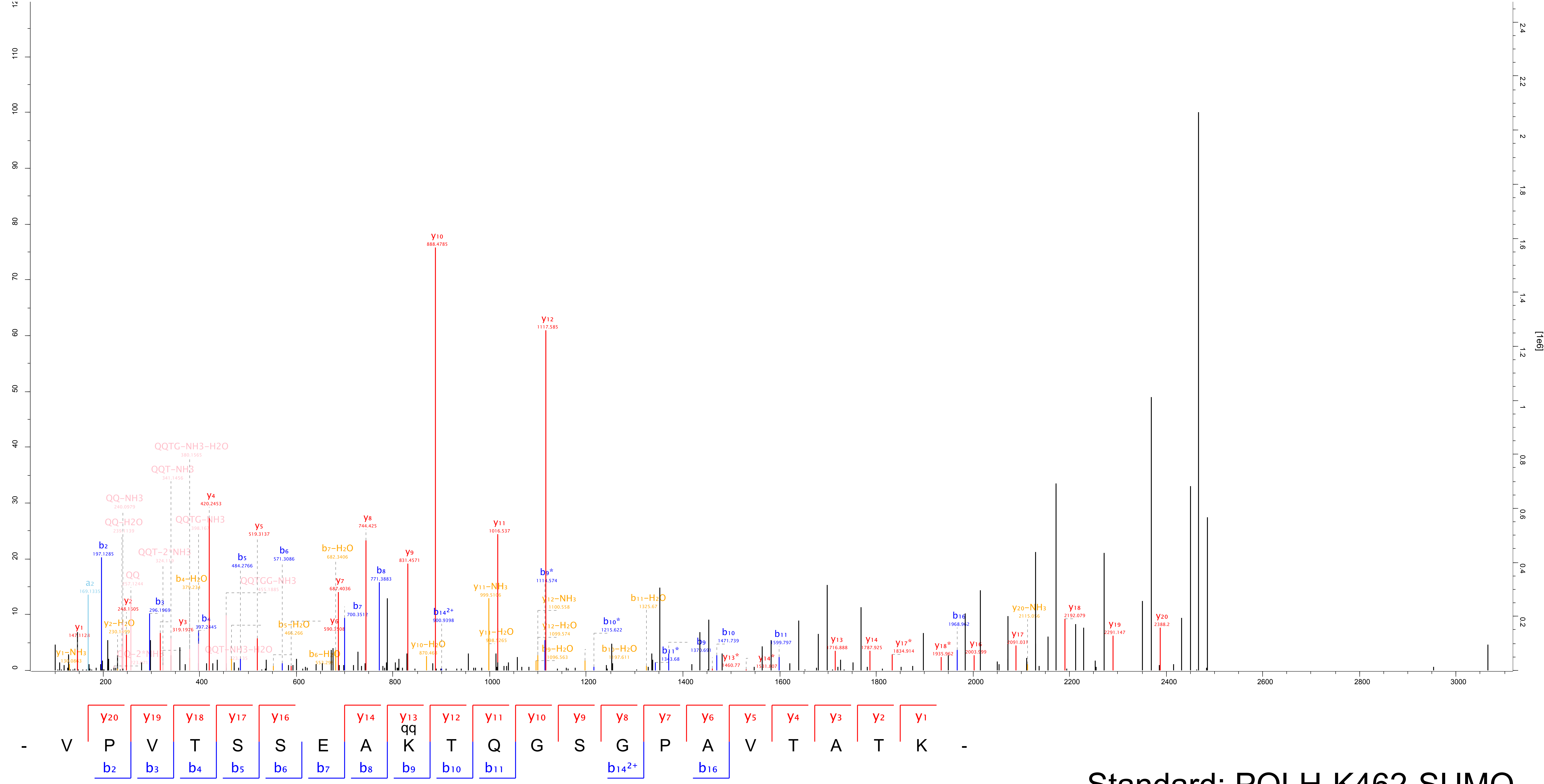

Standard: POLH-K462-SUMO

Raw file	Scan	Method	Score	m/z	Gene names
QE6_LC6_IAH_SUMO_HeLa_M_1_F3	8492	FTMS; HCD	324.12	830.1	POLH

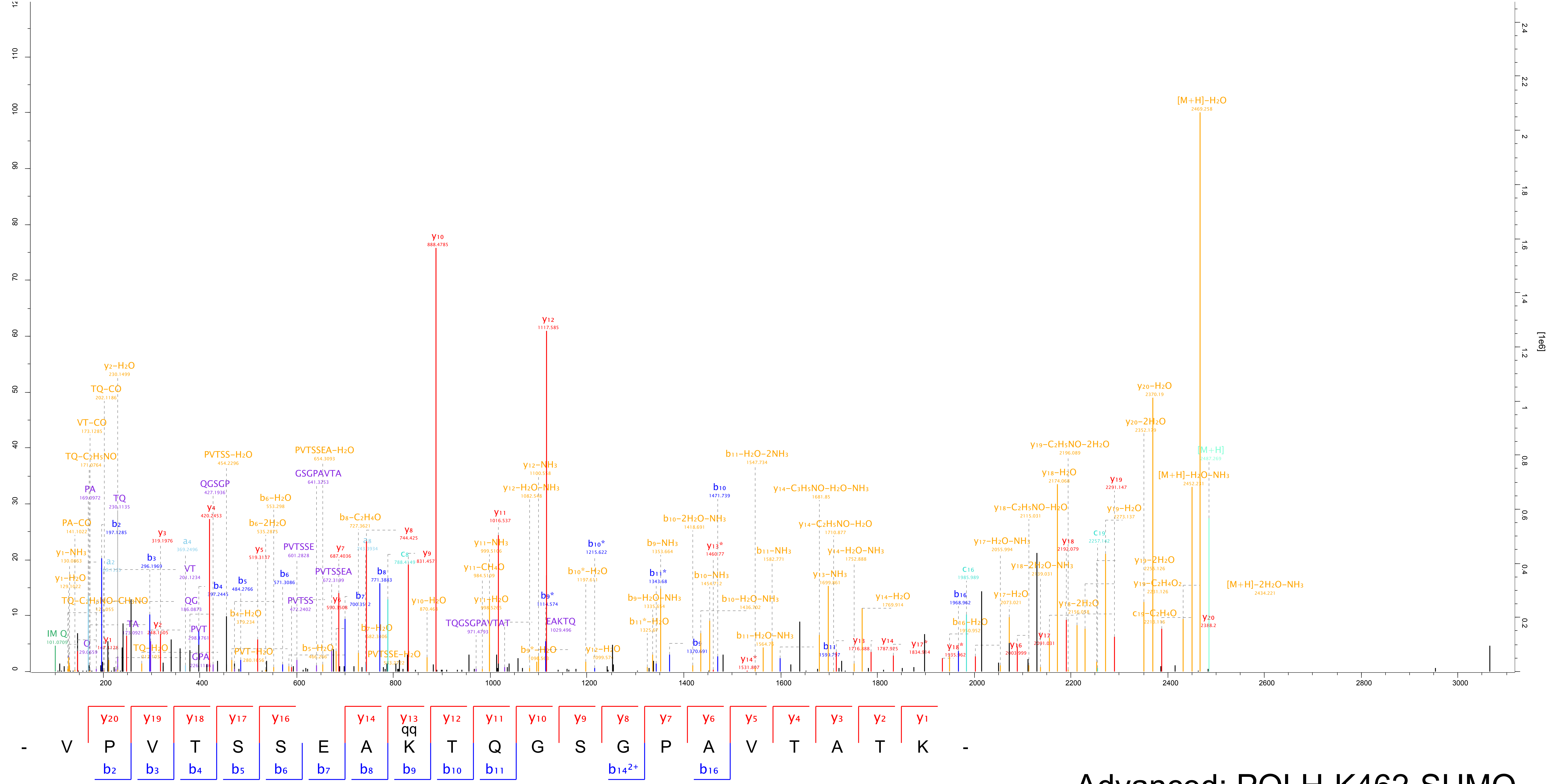

Advanced: POLH-K462-SUMO

Raw file	Scan	Method	Score	m/z	Gene names
QE6_LC6_IAH_SUMO_HeLa_M_4_F3	4129	FTMS; HCD	154.49	572.97	POLH

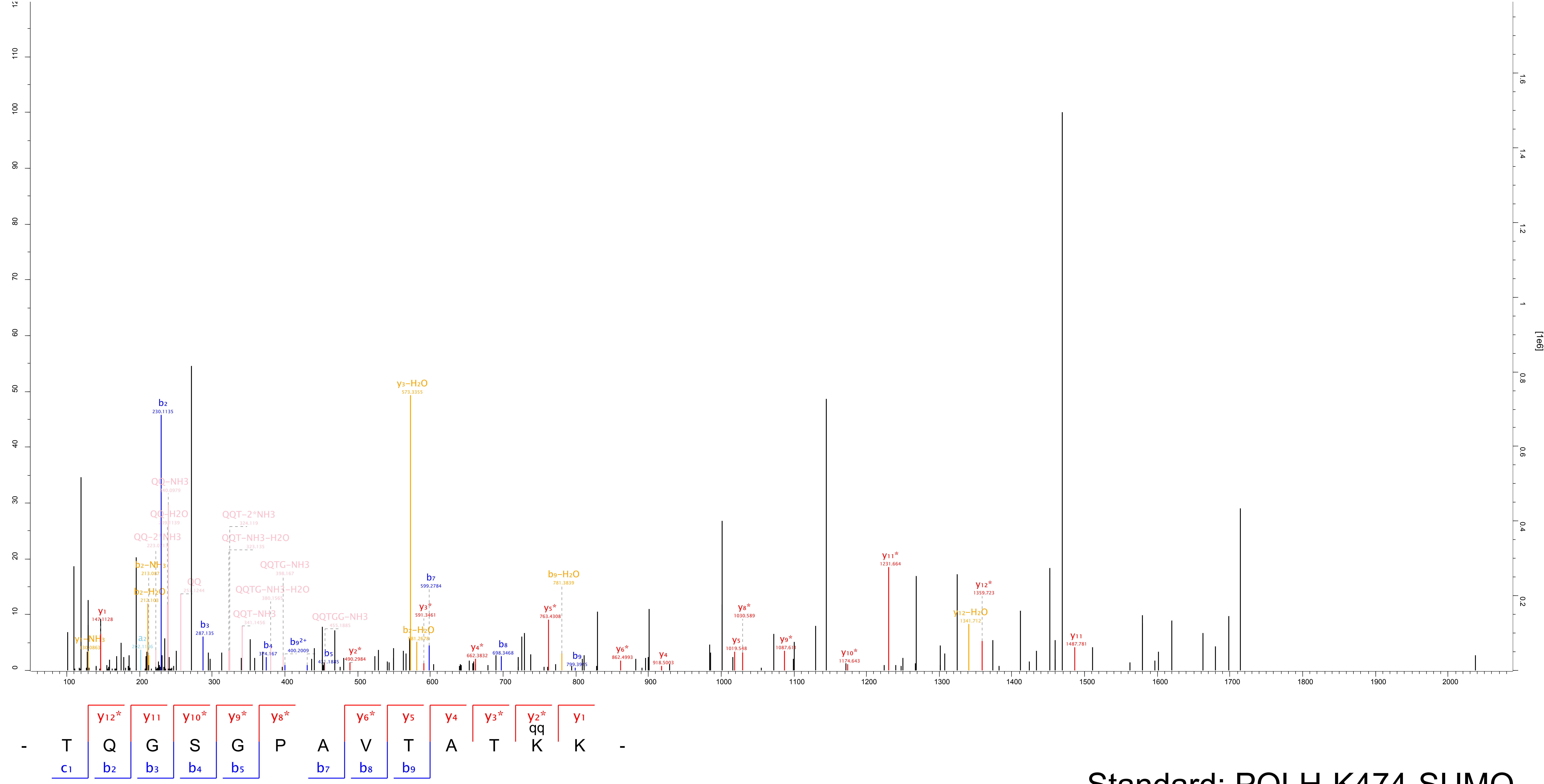

Standard: POLH-K474-SUMO

Raw file	Scan	Method	Score	m/z	Gene names
QE6_LC6_IAH_SUMO_HeLa_M_4_F3	4129	FTMS; HCD	154.49	572.97	POLH

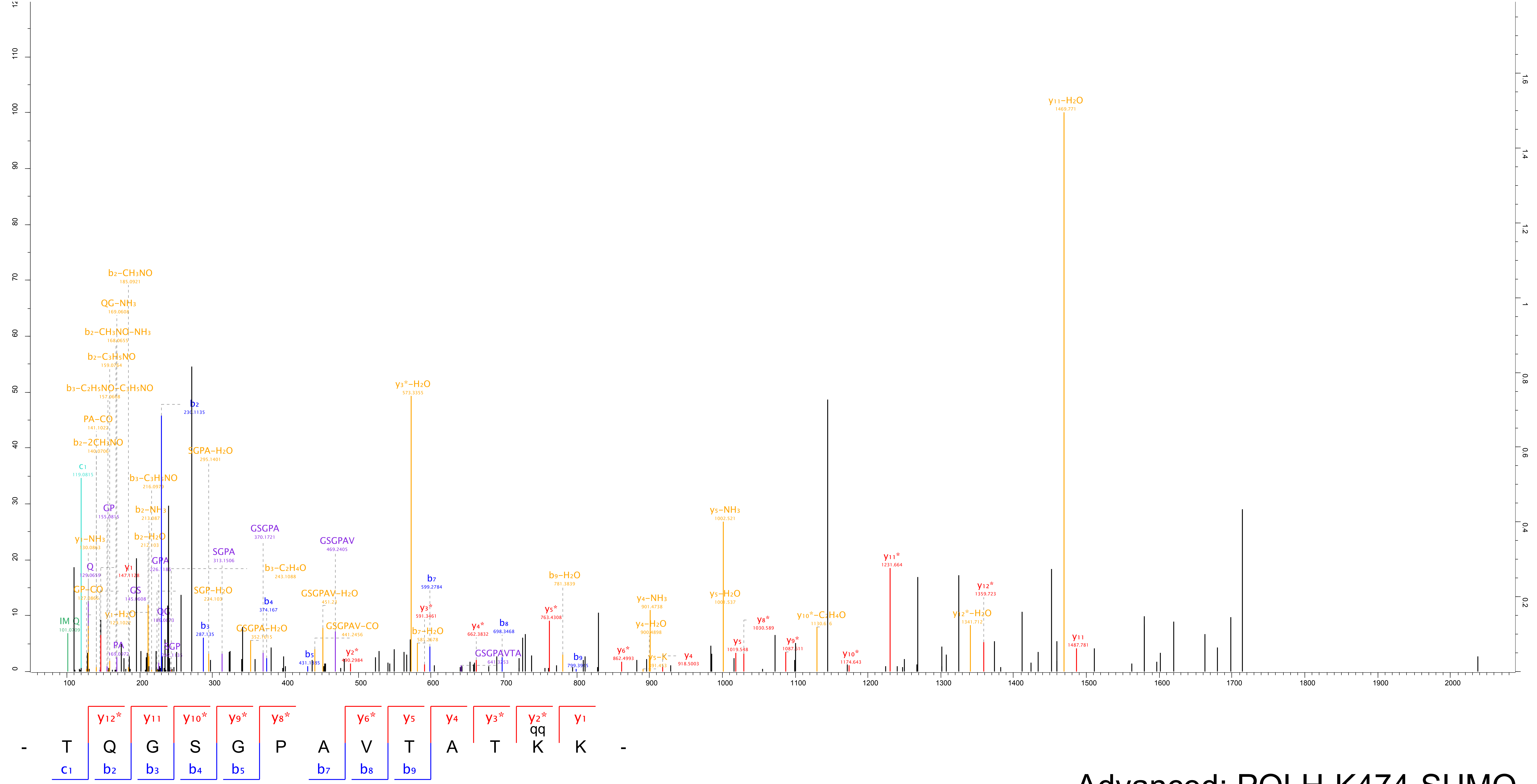

Advanced: POLH-K474-SUMO

Raw file	Scan	Method	Score	m/z	Gene names
QE6_LC6_IAH_SUMO_HeLa_M_2_F4	18596	FTMS; HCD	152	619.99	POLH

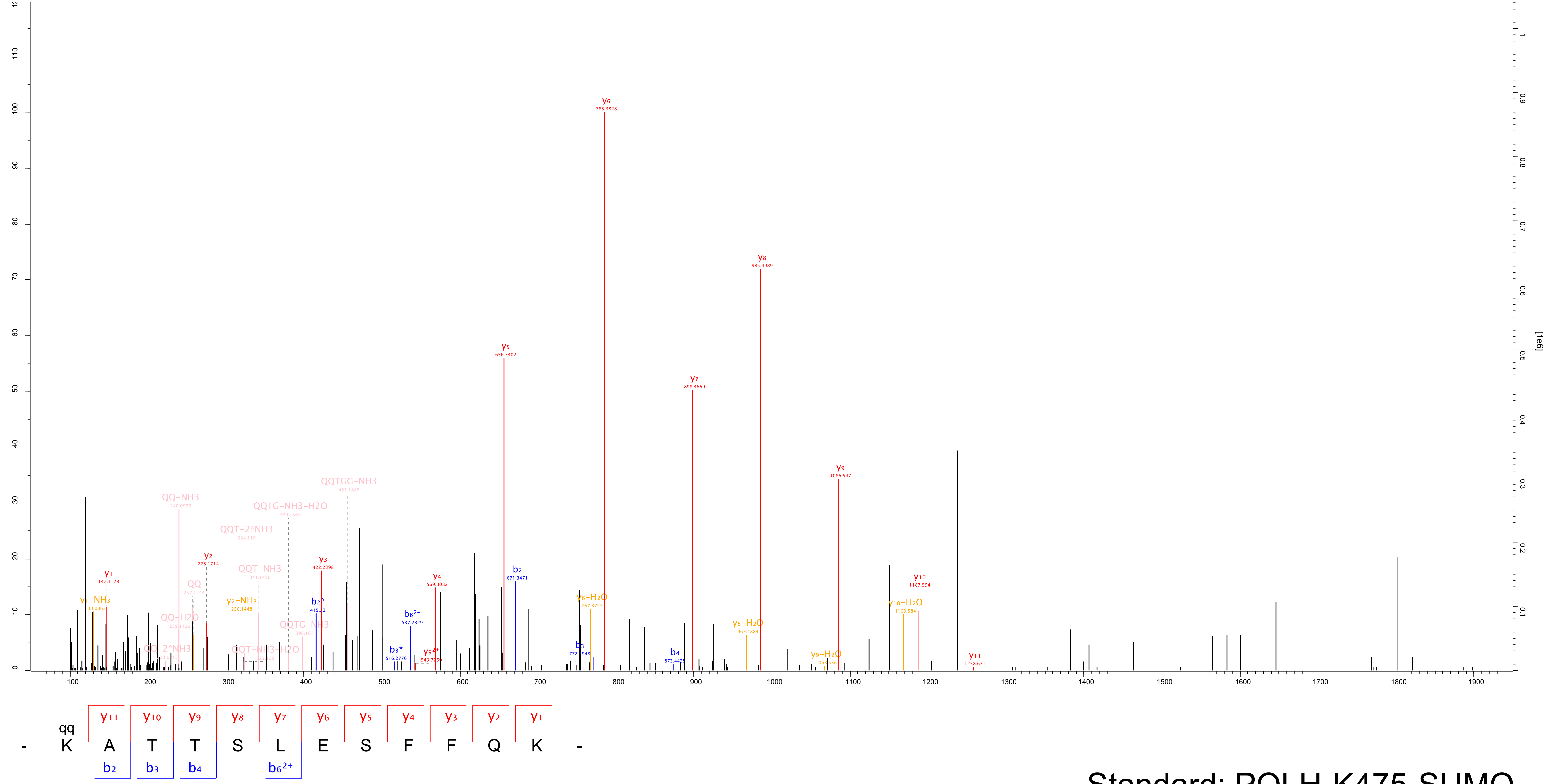

Standard: POLH-K475-SUMO

Raw file	Scan	Method	Score	m/z	Gene names
QE6_LC6_IAH_SUMO_HeLa_M_2_F4	18596	FTMS; HCD	152	619.99	POLH

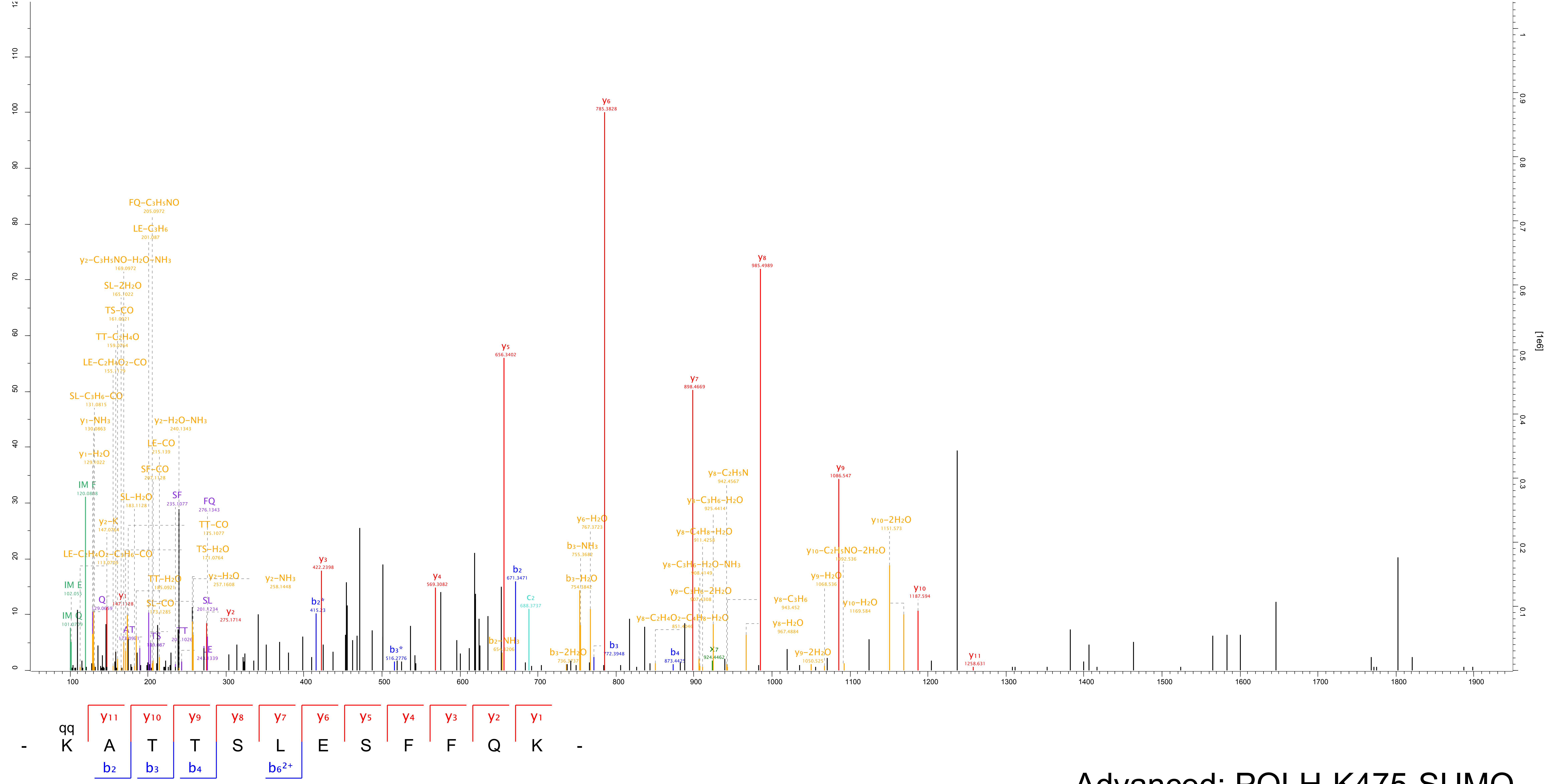

Advanced: POLH-K475-SUMO

Raw file	Scan	Method	Score	m/z	Gene names
QE6_LC6_IAH_SUMO_HeLa_M_2_F4	22242	FTMS; HCD	142.07	720.03	POLH

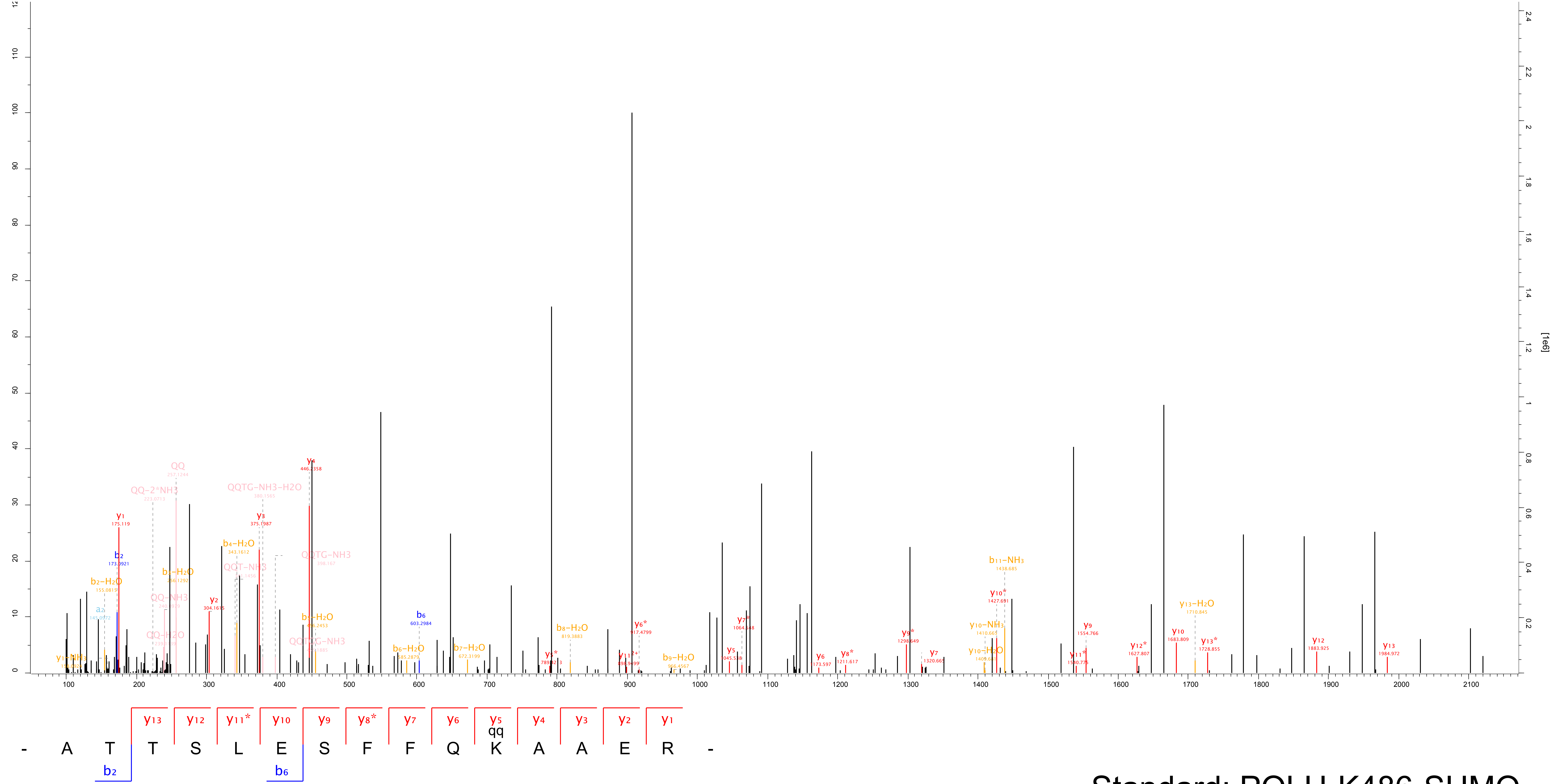

Standard: POLH-K486-SUMO

Raw file	Scan	Method	Score	m/z	Gene names
QE6_LC6_IAH_SUMO_HeLa_M_2_F4	22242	FTMS; HCD	142.07	720.03	POLH

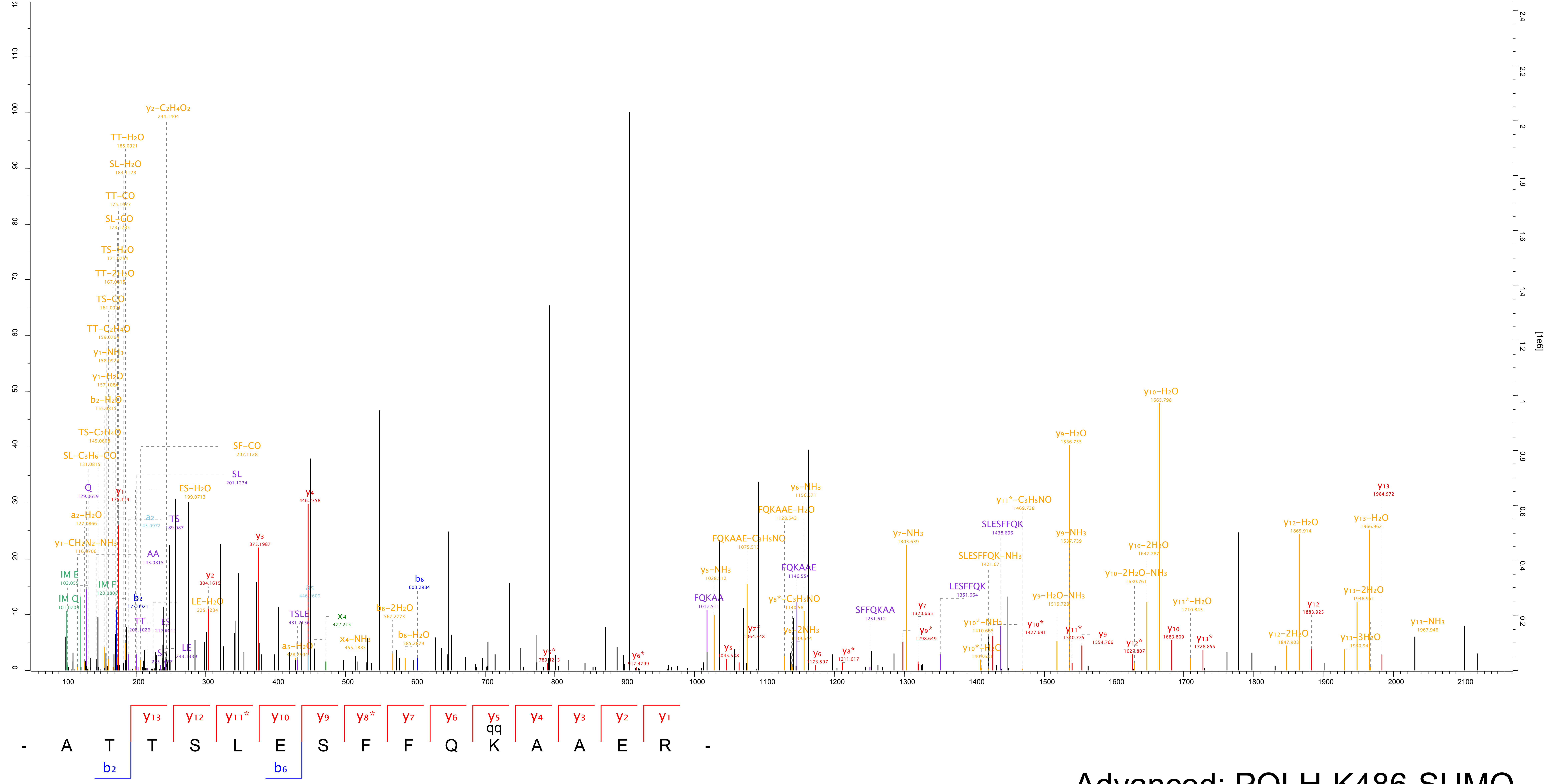

Advanced: POLH-K486-SUMO

Raw file	Scan	Method	Score	m/z	Gene names
QE6_LC6_IAH_SUMO_HeLa_M_4_F5	16043	FTMS; HCD	60.47	935.14	POLH

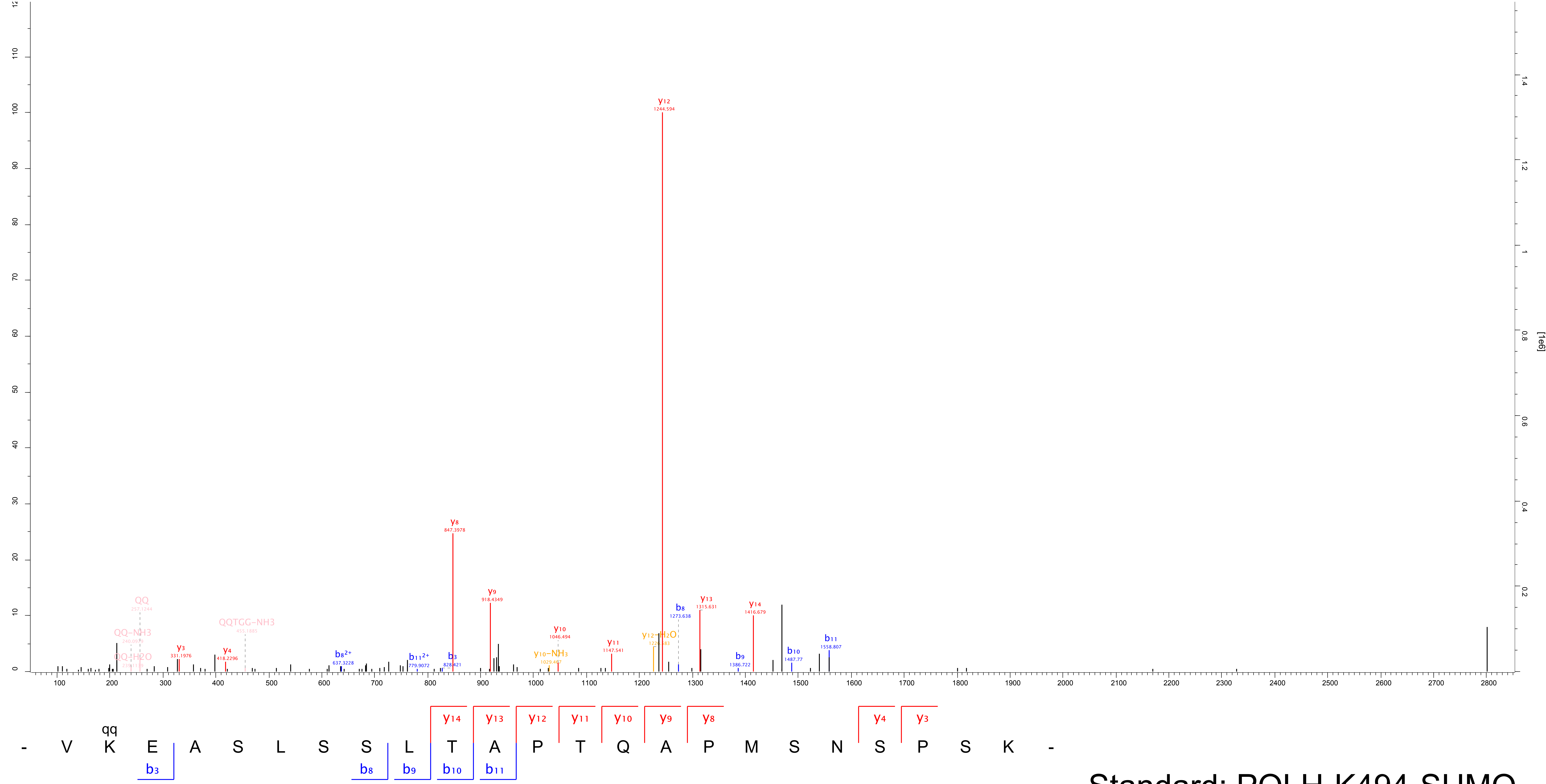

Standard: POLH-K494-SUMO

Raw file	Scan	Method	Score	m/z	Gene names
QE6_LC6_IAH_SUMO_HeLa_M_4_F5	16043	FTMS; HCD	60.47	935.14	POLH

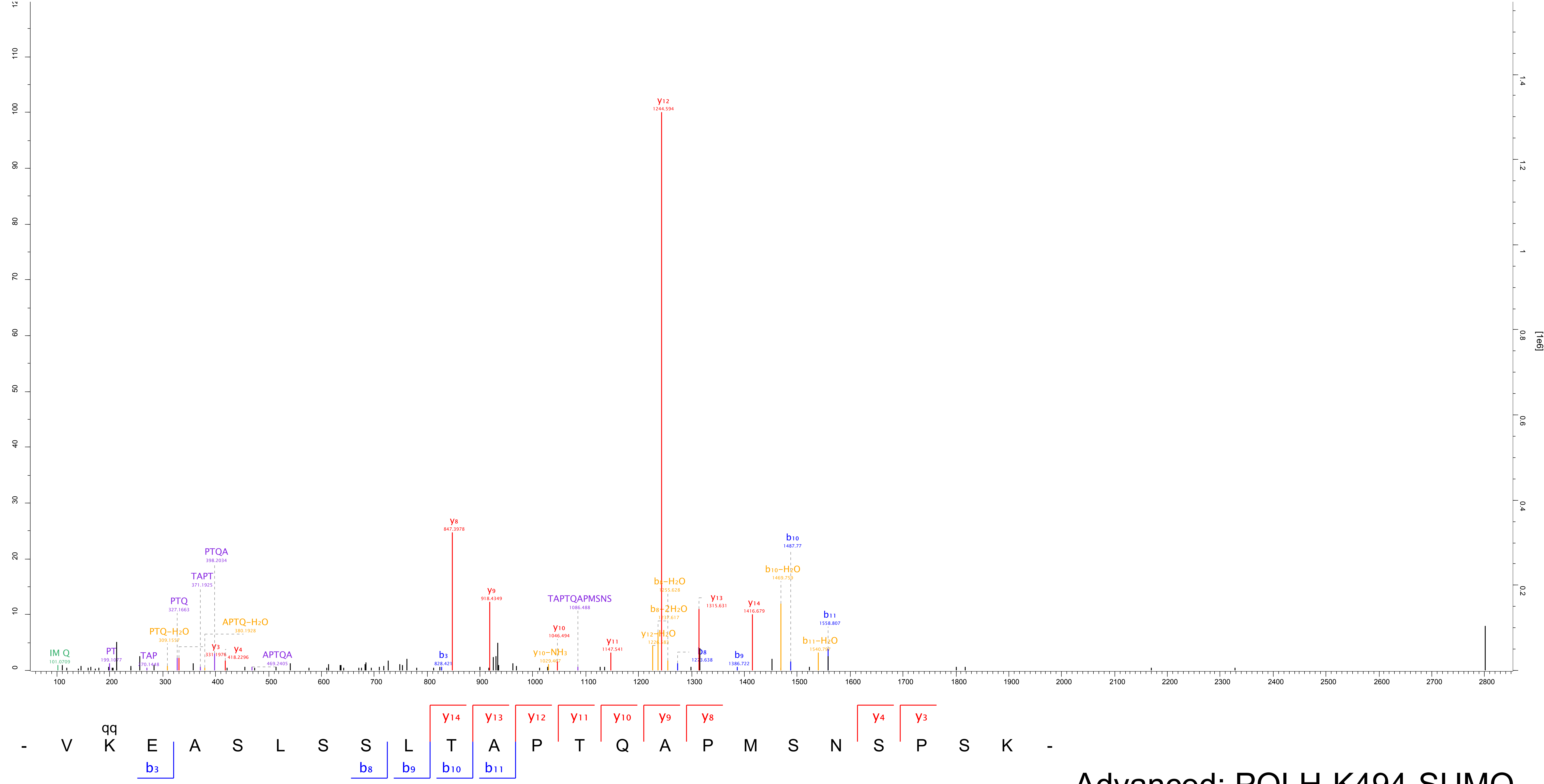

Advanced: POLH-K494-SUMO

Raw file	Scan	Method	Score	m/z	Gene names
QE6_LC6_IAH_SUMO_HeLa_M_4_F5	27746	FTMS; HCD	68.16	1140.06	POLH

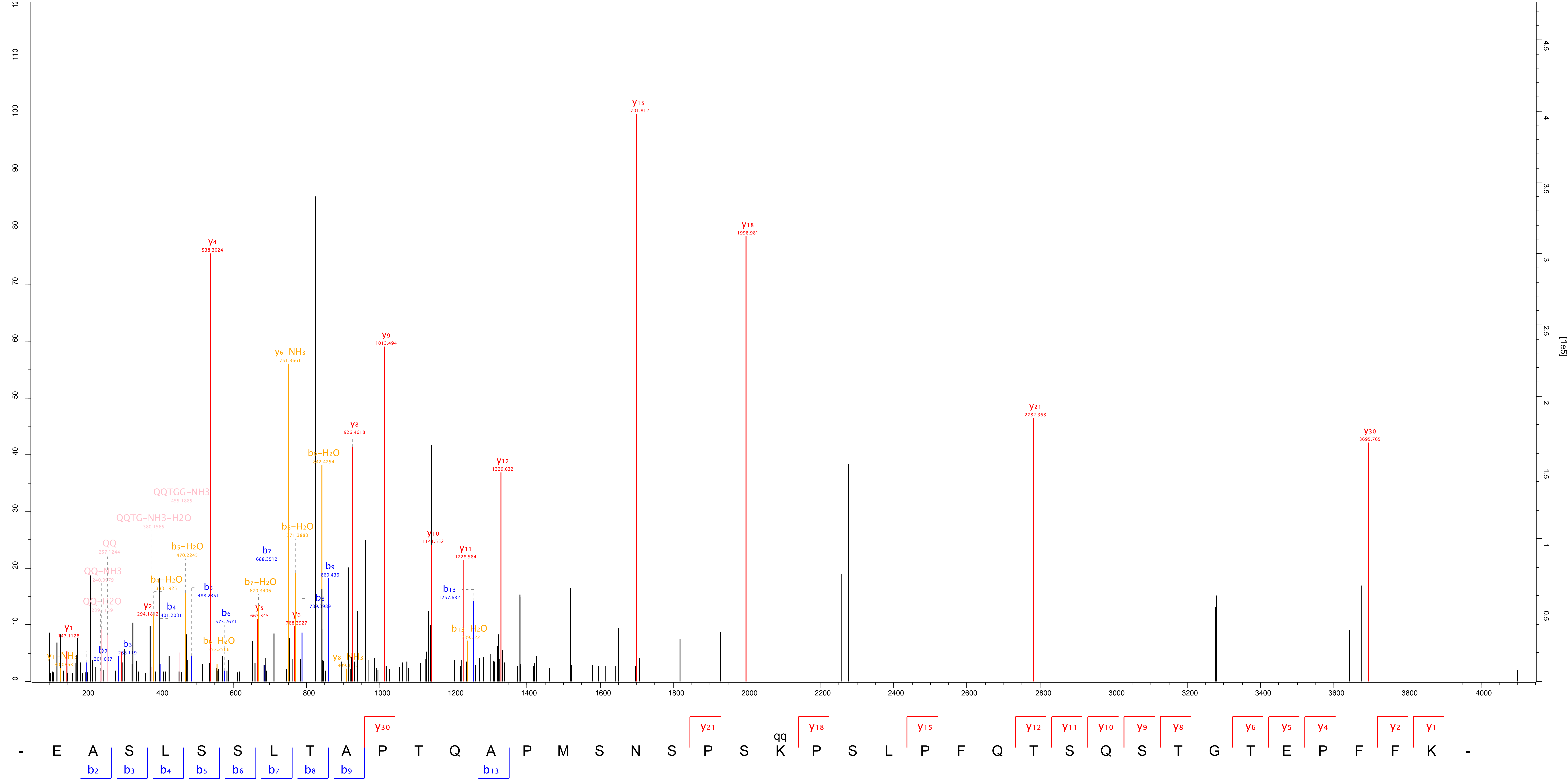

Standard: POLH-K515-SUMO

Raw file	Scan	Method	Score	m/z	Gene names
QE6_LC6_IAH_SUMO_HeLa_M_4_F5	27746	FTMS; HCD	68.16	1140.06	POLH

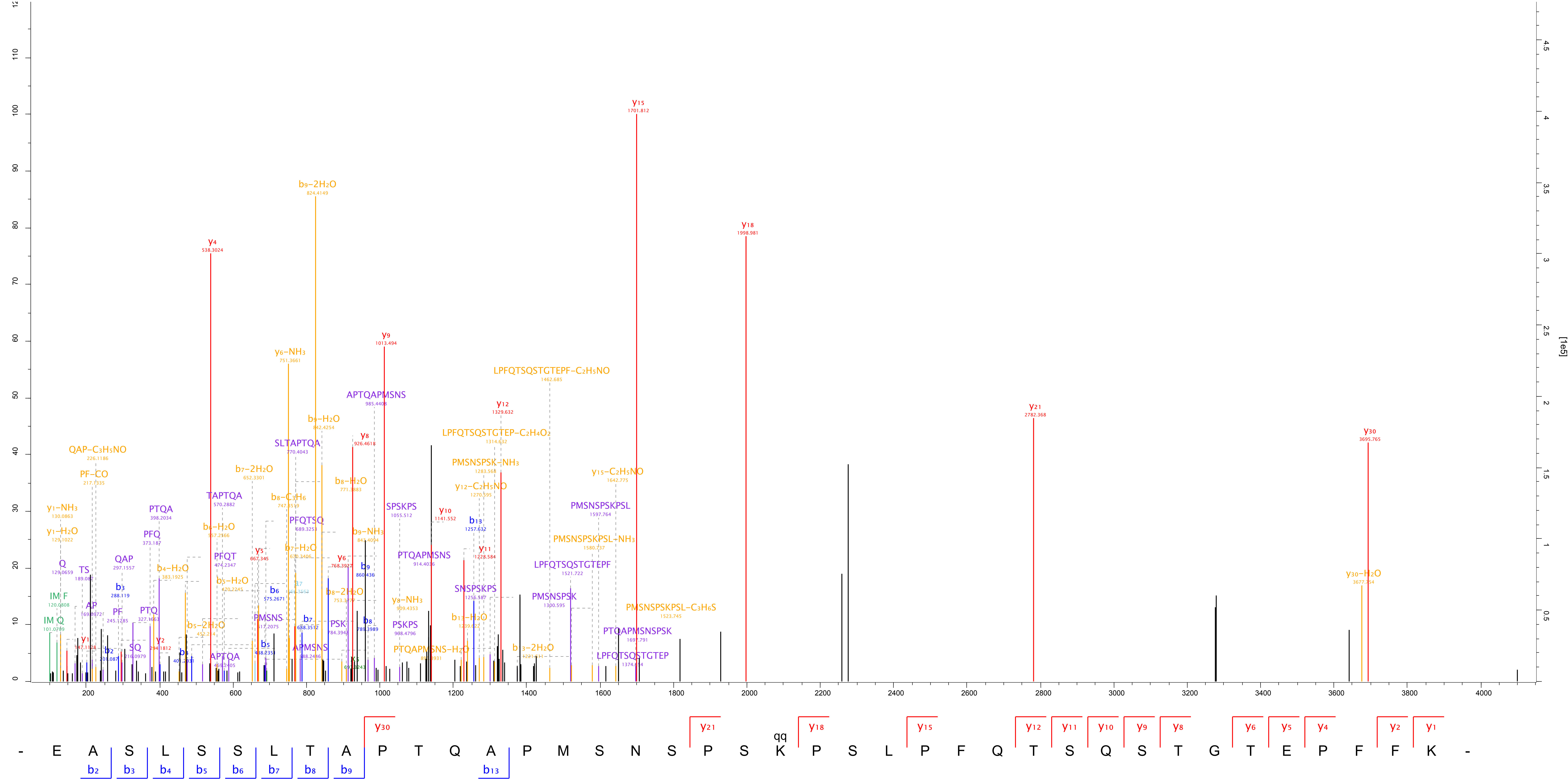

Raw file	Scan	Method	Score	m/z	Gene names
QE6_LC6_IAH_SUMO_HeLa_M_1_F5	16055	FTMS; HCD	184.83	909.79	POLH

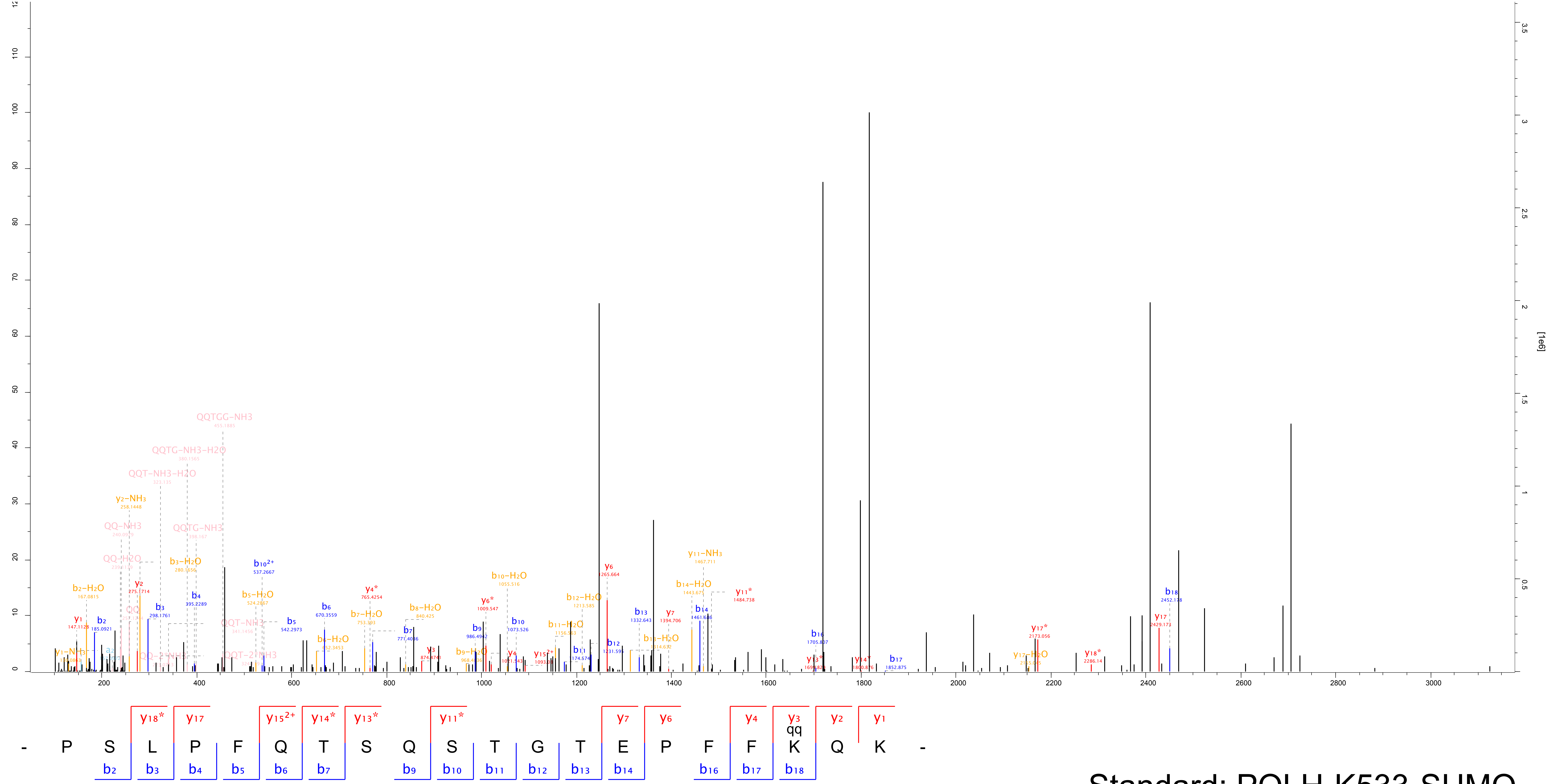

Standard: POLH-K533-SUMO

Raw file	Scan	Method	Score	m/z	Gene names
QE6_LC6_IAH_SUMO_HeLa_M_1_F5	16055	FTMS; HCD	184.83	909.79	POLH

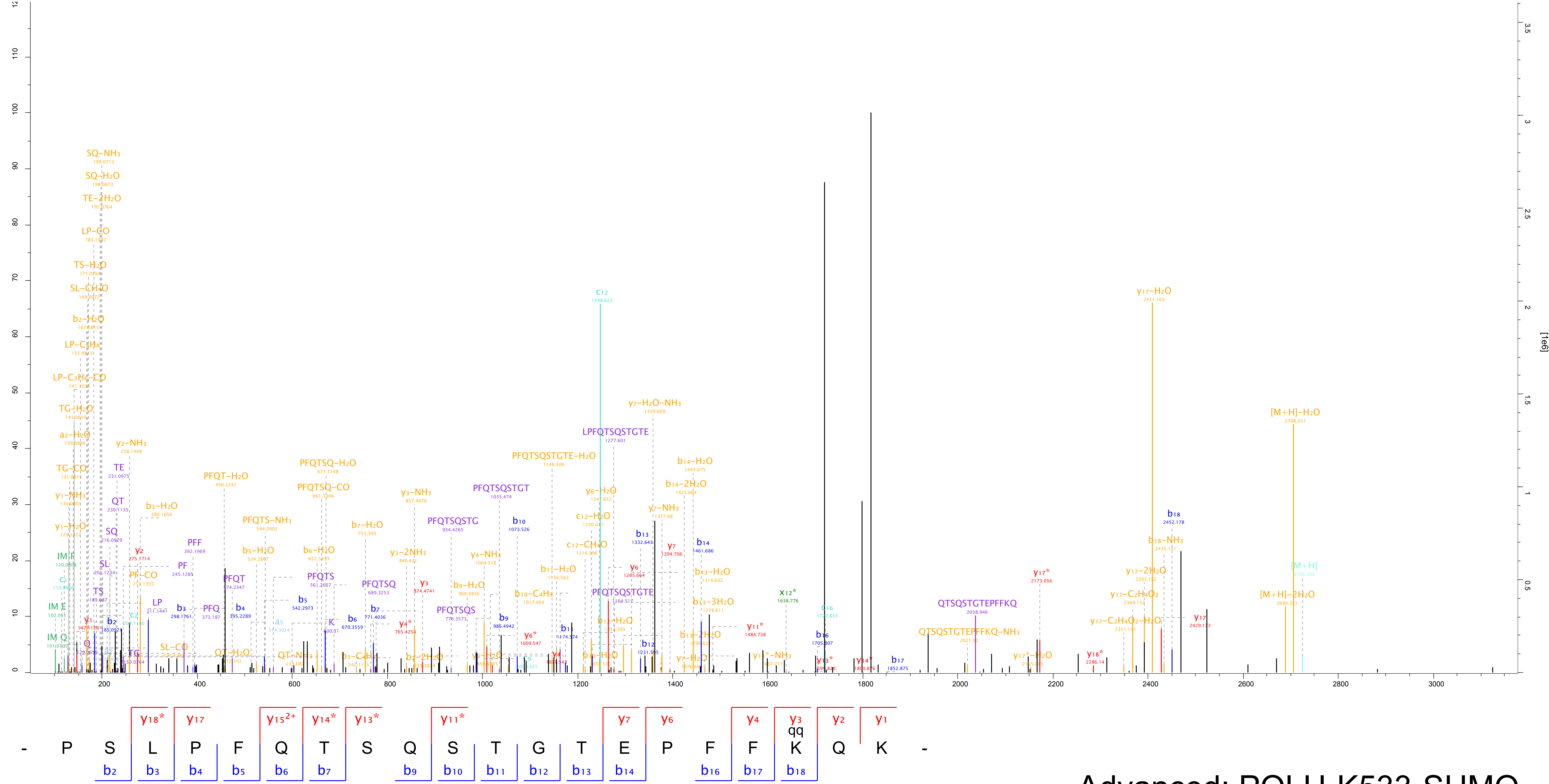

Advanced: POLH-K533-SUMO

Raw file	Scan	Method	Score	m/z	Gene names
QE6_LC6_IAH_SUMO_HeLa_M_1_F4	23233	FTMS; HCD	127.24	1174.57	POLH

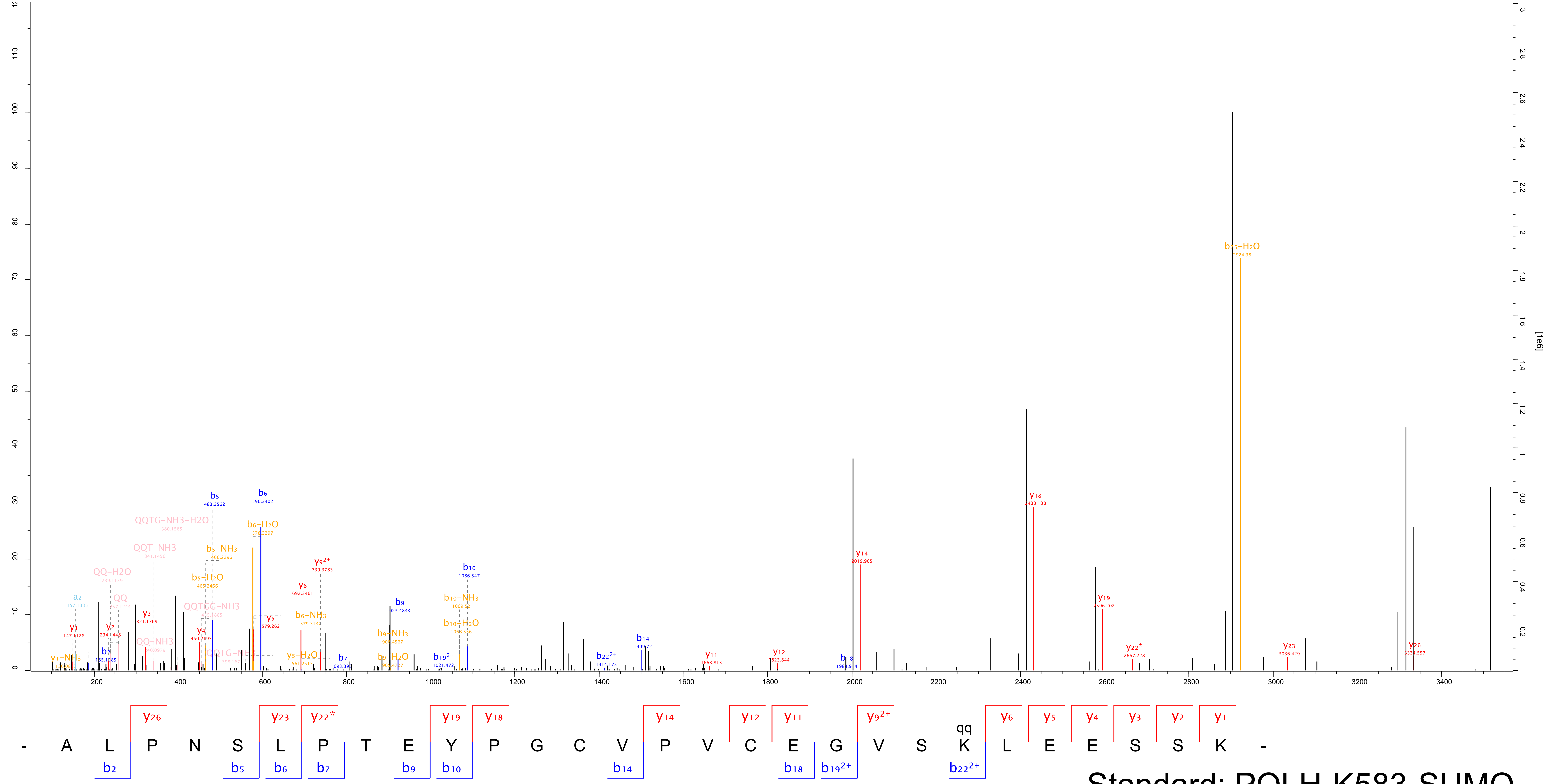

Standard: POLH-K583-SUMO

Raw file	Scan	Method	Score	m/z	Gene names
QE6_LC6_IAH_SUMO_HeLa_M_1_F4	23233	FTMS; HCD	127.24	1174.57	POLH

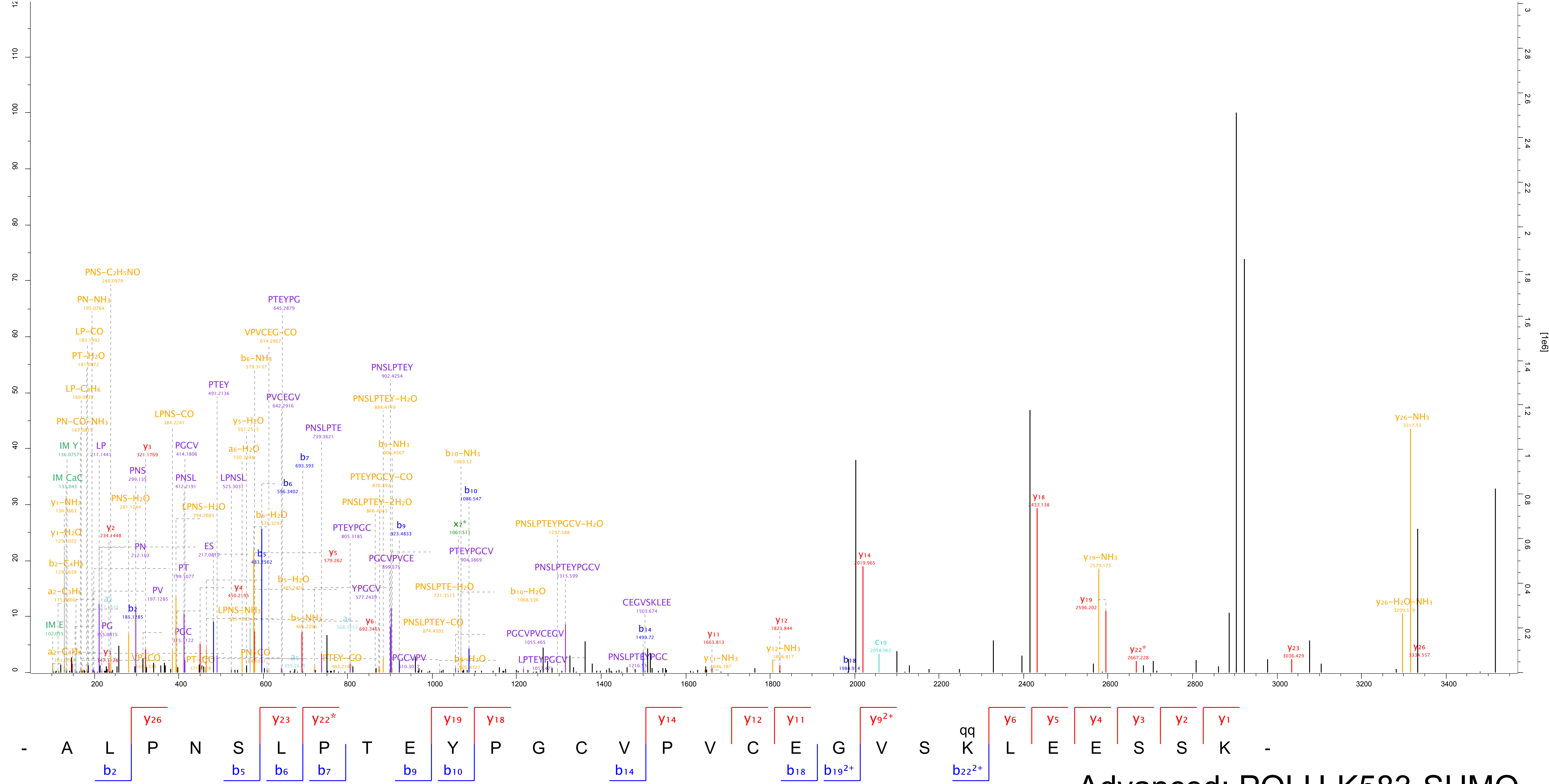

Advanced: POLH-K583-SUMO

Raw file	Scan	Method	Score	m/z	Gene names
QE6_LC6_IAH_SUMO_HeLa_M_3_F2	16707	FTMS; HCD	58.35	684.32	POLH

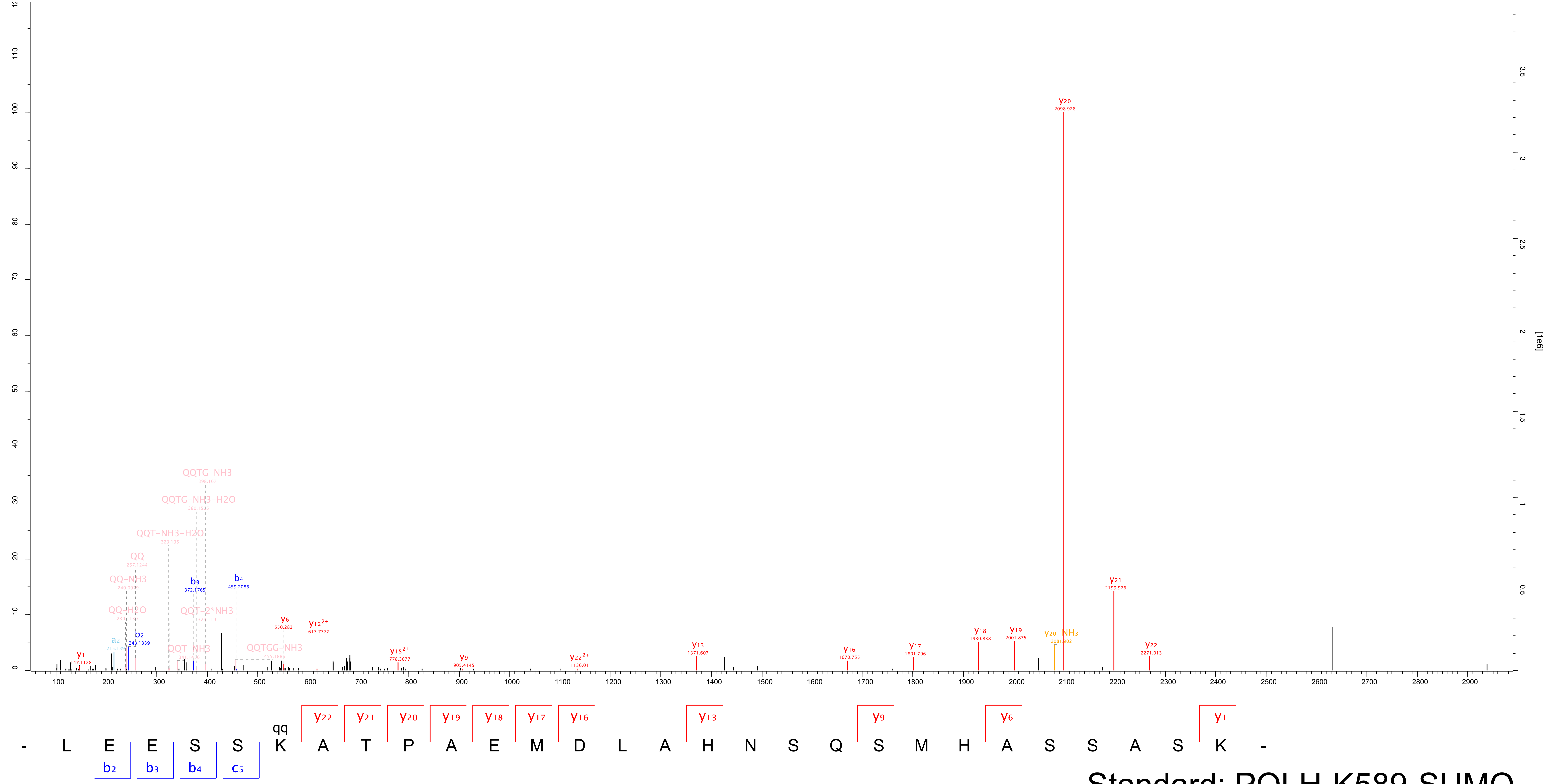

Standard: POLH-K589-SUMO

Raw file	Scan	Method	Score	m/z	Gene names
QE6_LC6_IAH_SUMO_HeLa_M_3_F2	16707	FTMS; HCD	58.35	684.32	POLH

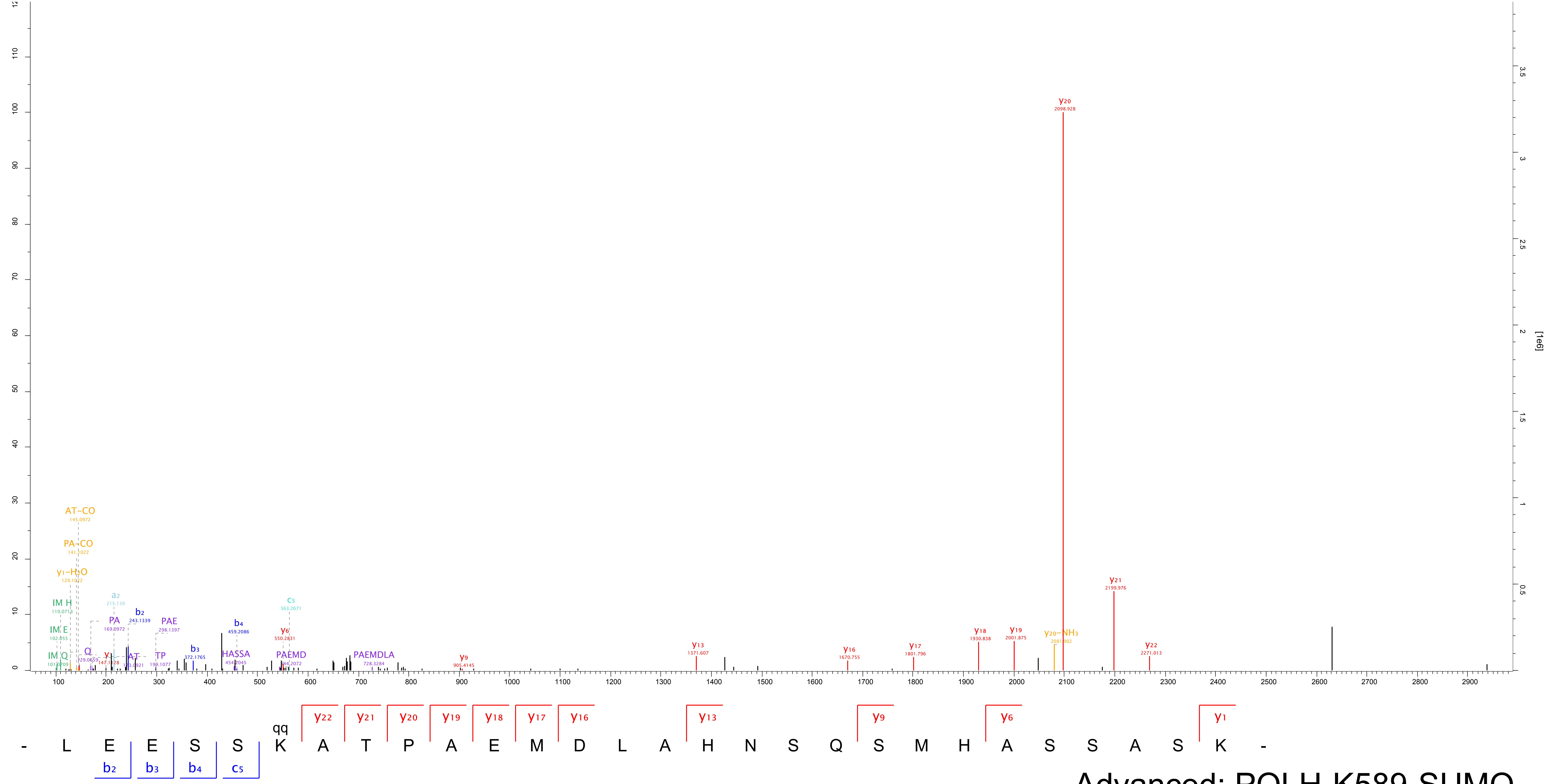

Advanced: POLH-K589-SUMO

Raw file	Scan	Method	Score	m/z	Gene names
QE6_LC6_IAH_SUMO_HeLa_M_4_F3	27256	FTMS; HCD	103.85	1099.56	POLH

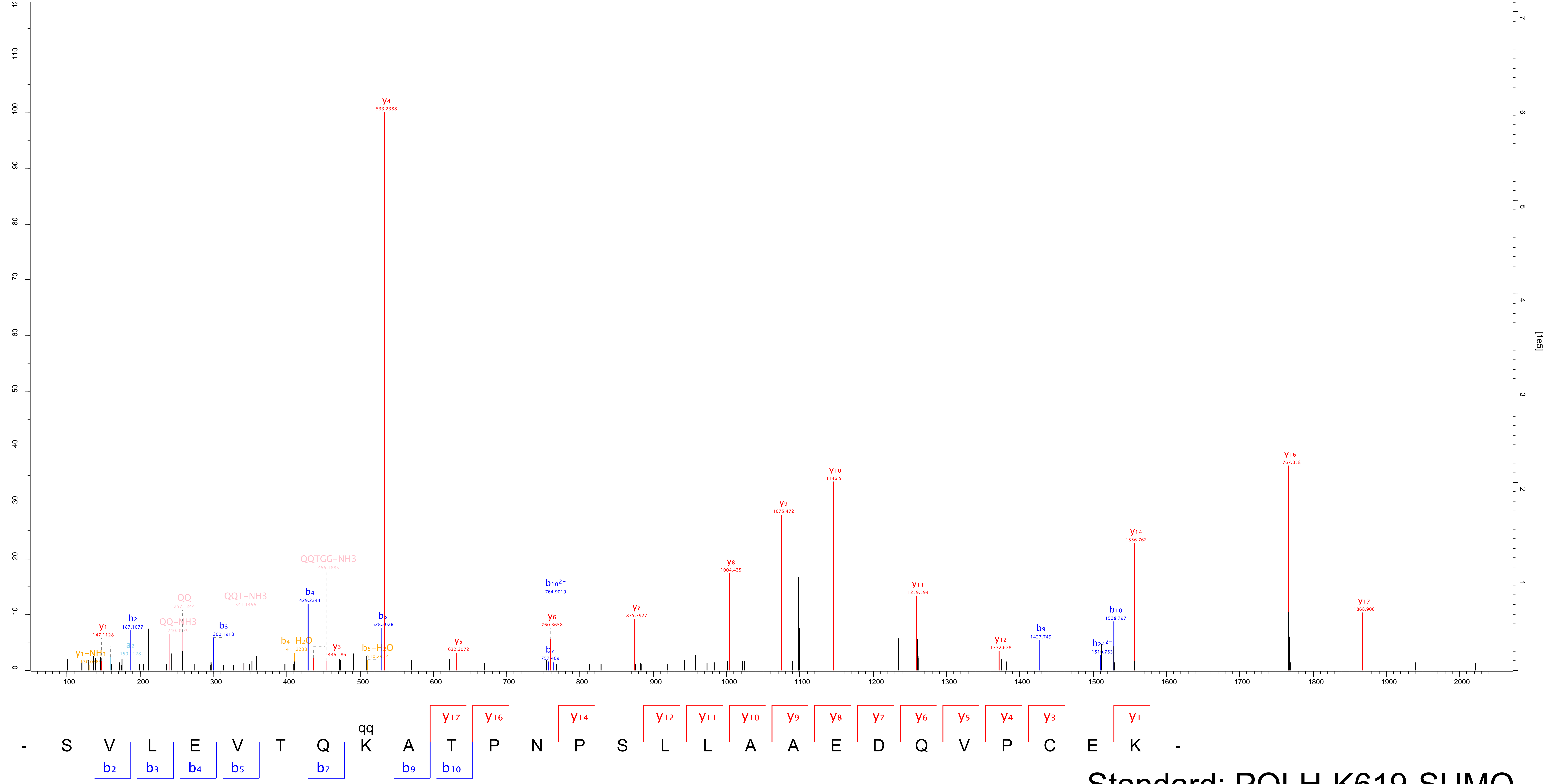

Standard: POLH-K619-SUMO

Raw file	Scan	Method	Score	m/z	Gene names
QE6_LC6_IAH_SUMO_HeLa_M_4_F3	27256	FTMS; HCD	103.85	1099.56	POLH

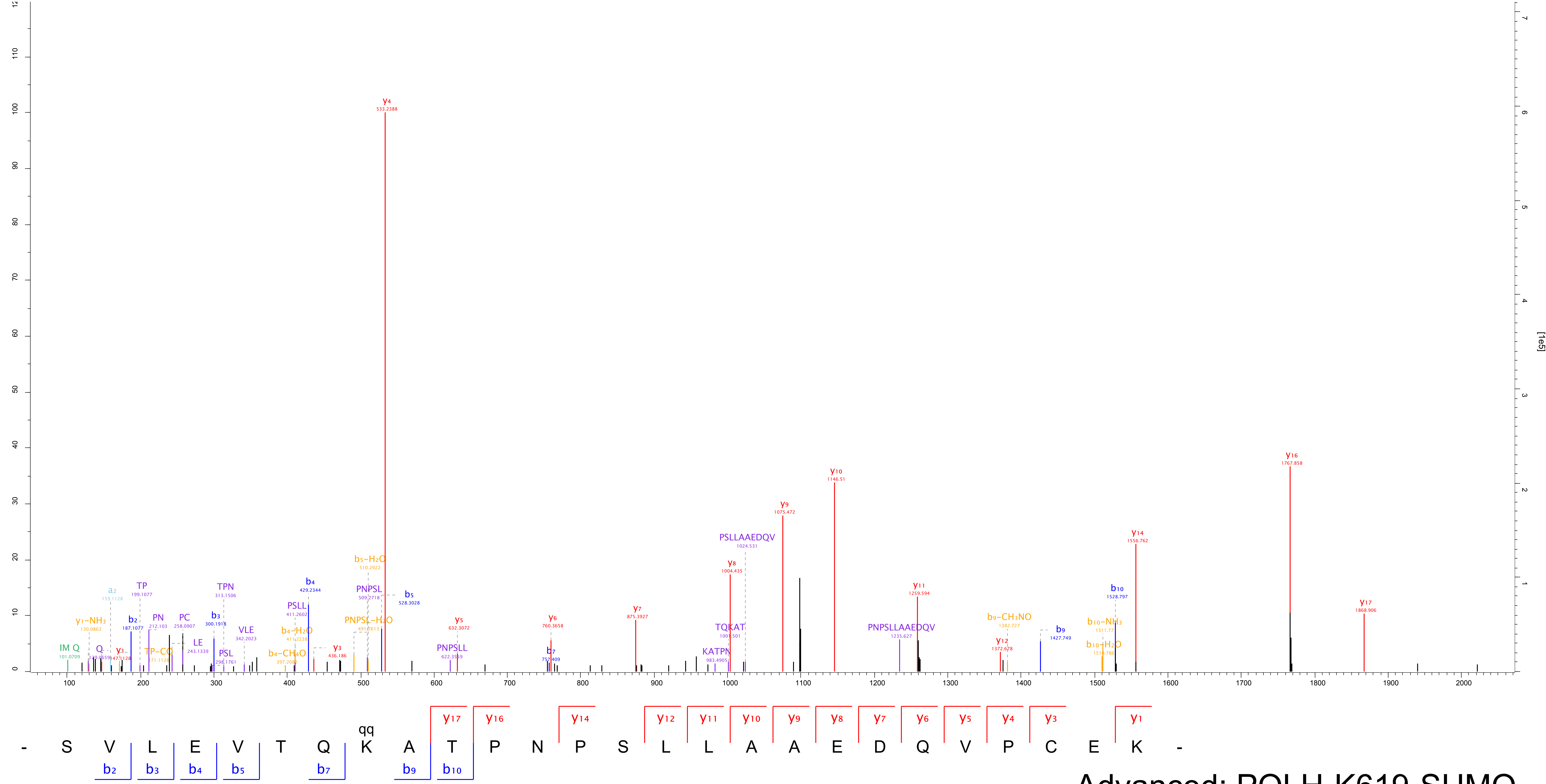

Advanced: POLH-K619-SUMO

Raw file	Scan	Method	Score	m/z	Gene names
QE6_LC6_IAH_SUMO_HeLa_M_1_F6	7778	FTMS; HCD	142.27	716.37	POLH

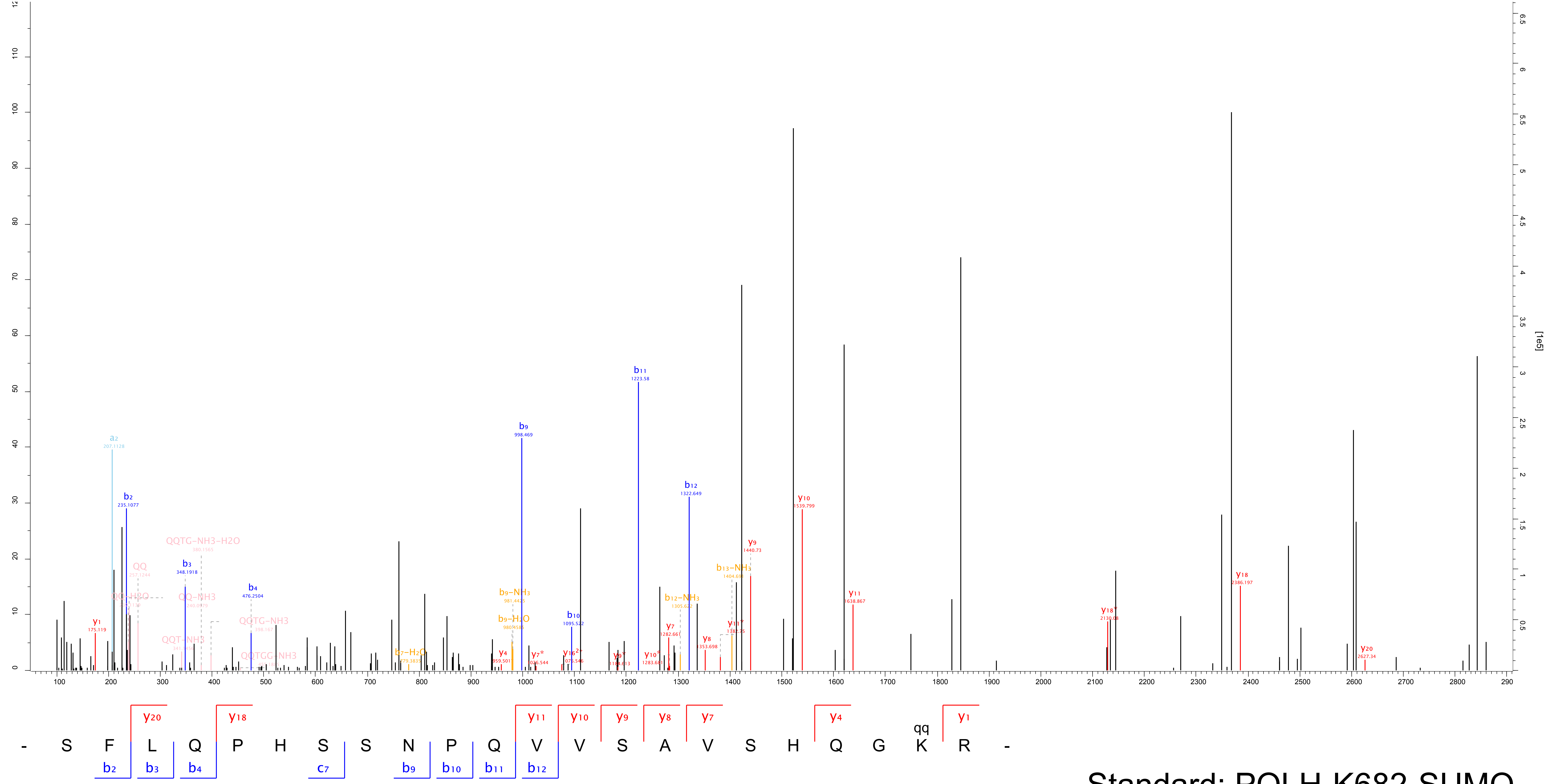

Standard: POLH-K682-SUMO

Raw file	Scan	Method	Score	m/z	Gene names
QE6_LC6_IAH_SUMO_HeLa_M_1_F6	7778	FTMS; HCD	142.27	716.37	POLH

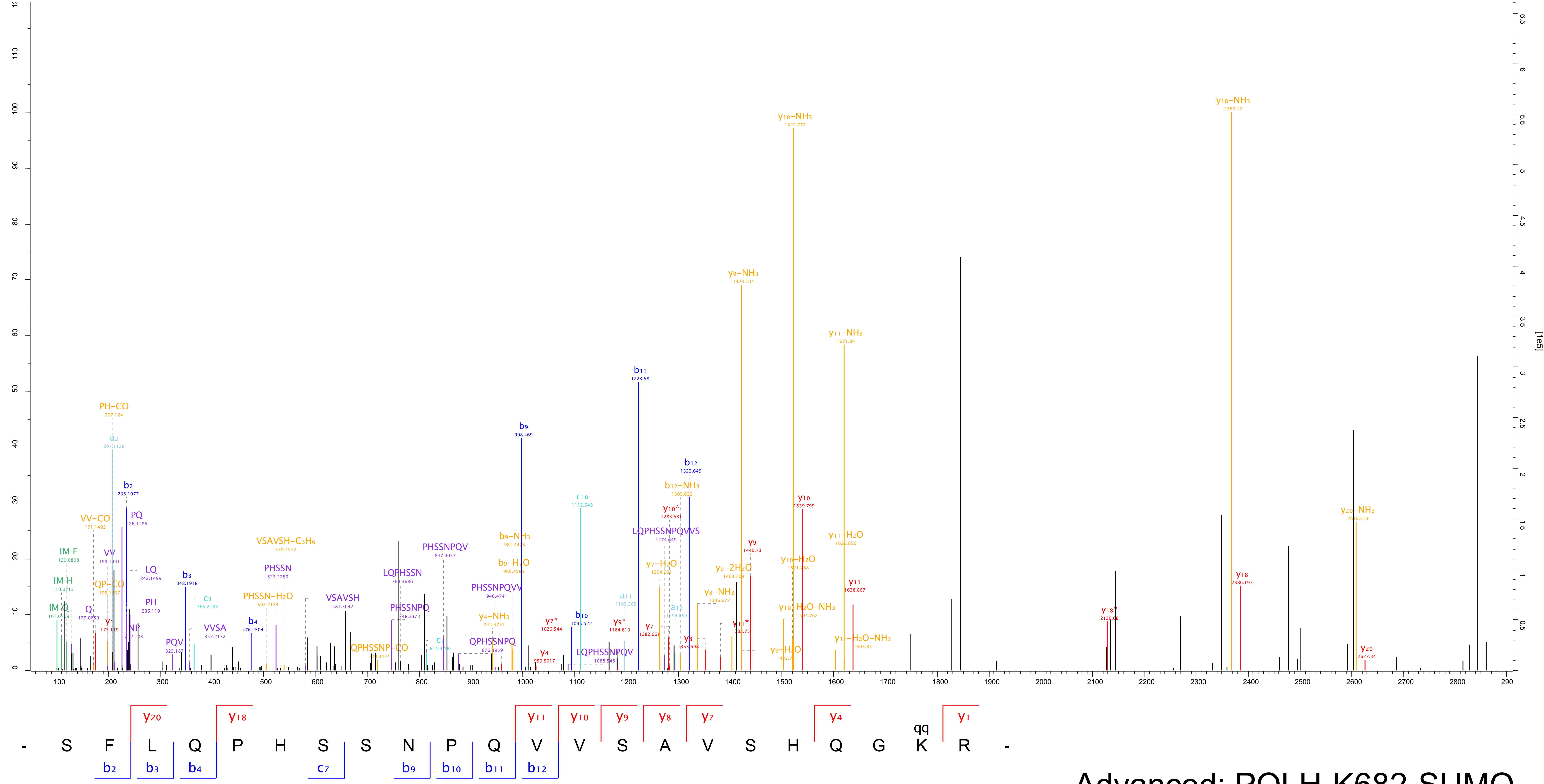

Advanced: POLH-K682-SUMO

Raw file	Scan	Method	Score	m/z	Gene names
QE6_LC6_IAH_SUMO_HeLa_M_1_F6	17905	FTMS; HCD	141.6	549.48	POLH

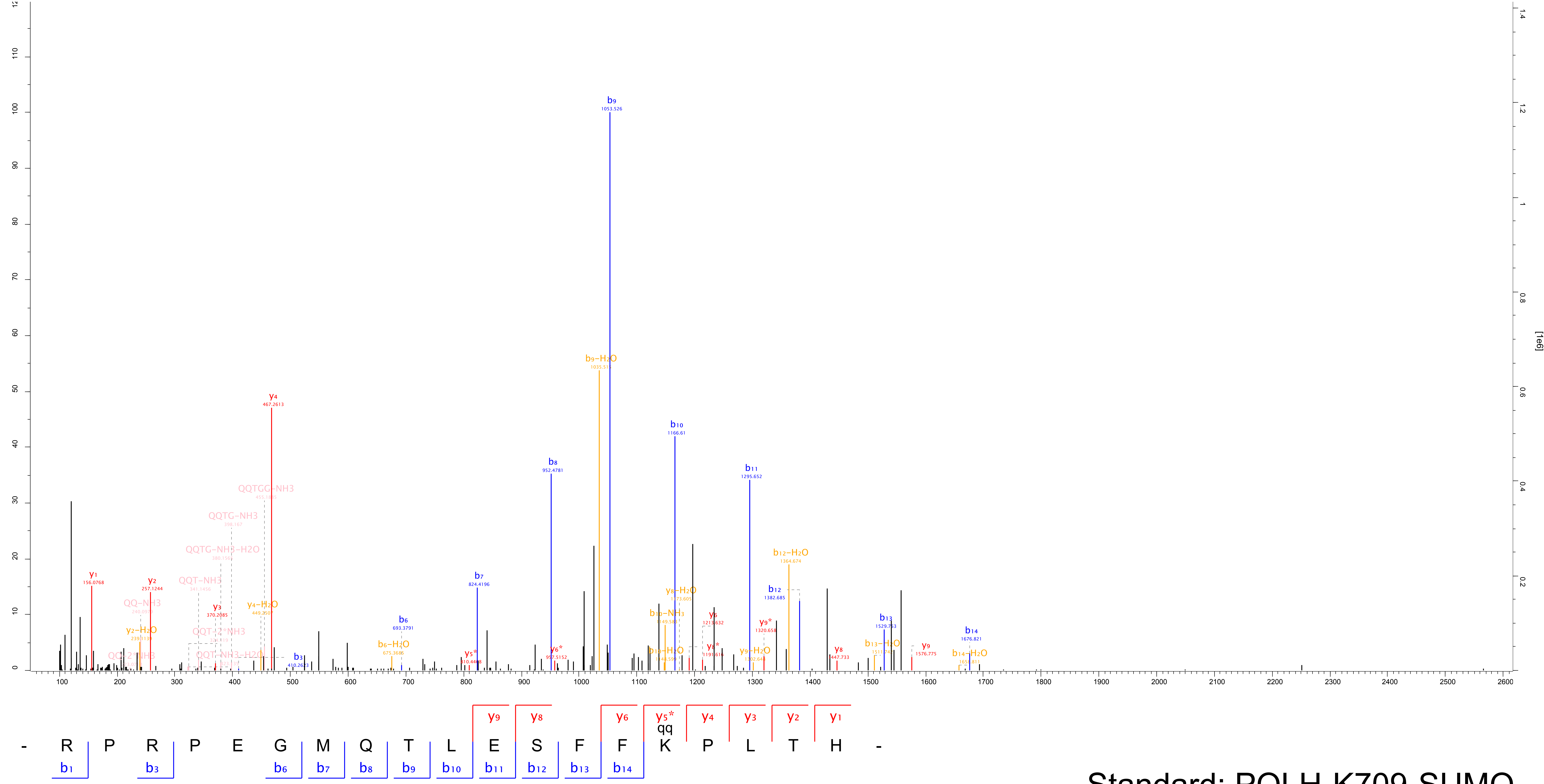

Standard: POLH-K709-SUMO

Raw file	Scan	Method	Score	m/z	Gene names
QE6_LC6_IAH_SUMO_HeLa_M_1_F6	17905	FTMS; HCD	141.6	549.48	POLH

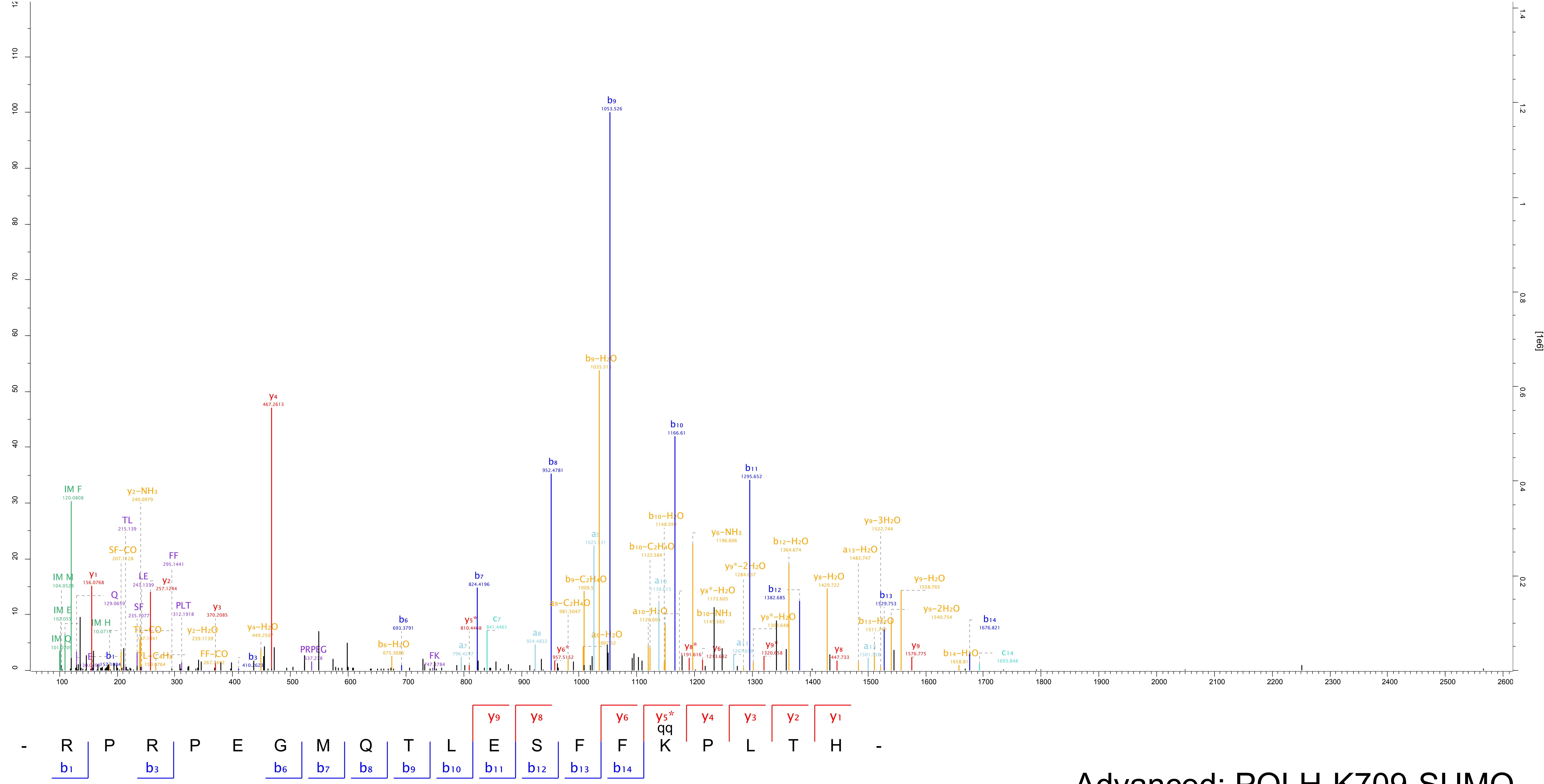

Advanced: POLH-K709-SUMO
